# Molecular Engineering to Tune Functionality: The Case of Cl-Substituted [Fe(terpy)_2_]^2+^

**DOI:** 10.1021/acs.inorgchem.3c00271

**Published:** 2023-04-11

**Authors:** Mariann Papp, Tamás Keszthelyi, Andor Vancza, Éva G. Bajnóczi, Éva Kováts, Zoltán Németh, Csilla Bogdán, Gábor Bazsó, Tamás Rozgonyi, György Vankó

**Affiliations:** † Wigner Research Centre for Physics, P.O. Box 49, H-1525 Budapest, Hungary; ‡ Hevesy György PhD School of Chemistry, Eötvös Loránd University, Pázmány Péter sétány 1/A, H-1117 Budapest, Hungary; § Department of Physical Chemistry and Materials Science, Faculty of Chemical Technology and Biotechnology, Budapest University of Technology and Economics, Műegyetem rkp. 3, H-1111 Budapest, Hungary

## Abstract

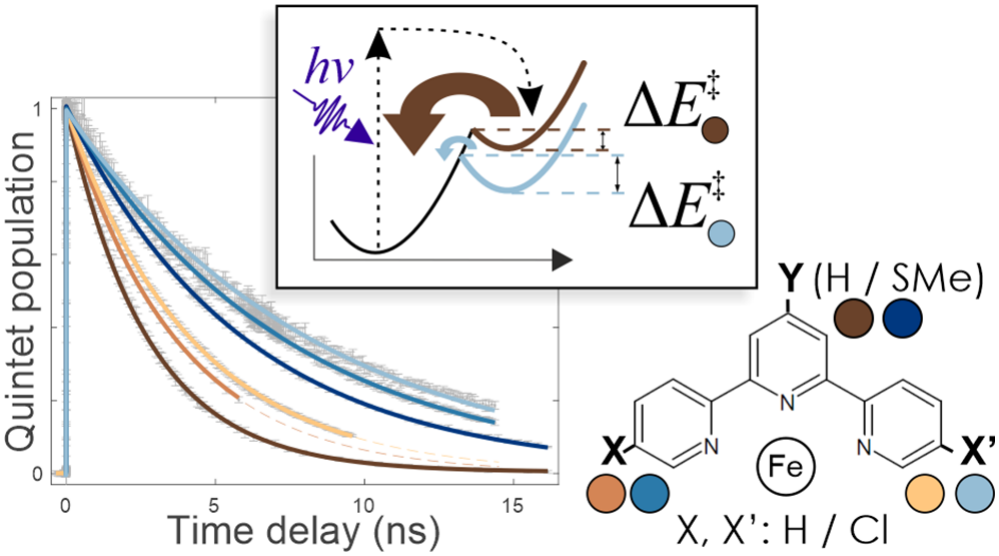

The properties of transition-metal complexes and their chemical dynamics can be effectively modified with ligand substitutions, and theory can be a great aid to such molecular engineering. In this paper, we first theoretically explored how substitution with a Cl atom at different positions of the terpyridine ligand affects the electronic structure of the [Fe(terpy)_2_]^2+^ complex. We found that besides the substitution at position 4′, the next most promising candidate to cause substantial electronic effects is that where the side pyridine ring is substituted at position 5 (β). Therefore, next, we examined in detail the Fe(II) complexes of the 5-chloro and 5,5″-dichloro derivatives of terpy, theoretically and experimentally, to reveal how these substitutions modify the ground state properties and the lifetime of the excited quintet state in such complexes. In addition, we extend the investigation to the complexes of the analogously substituted derivatives of 4′-SMe-terpy. The substitution at position(s) 5 (and 5″) with Cl lowers the energy of the quintet state and increases its lifetime; the results on the 4′-SMe-substituted complexes show similar changes with these two substitutions, verifying that these effects are more or less additive. This study contributes to the enhancement of our molecular engineering toolset for modifying the potential energy landscape of similar complexes.

## Abstract 2

Theory-based ligand engineering is applied to the [Fe(terpy)_2_]^2+^ complex with success: Cl substitution favorably modifies the electronic structure and potential energy landscape, resulting in increased excited quintet state lifetimes, thereby taking us closer to their application in molecular devices.

## Introduction

Photoactivated transition-metal-based functional molecules have long been studied due to their possible applications in molecular devices, e.g., molecular switches or data storing devices,^1−3^ as well as in light-harvesting systems and catalysts.^4,5^ Among them, low-spin Fe(II) complexes are considered promising candidates for molecular magnetic switches since many of them can be switched with light or other stimuli between their low-spin (singlet) ground state and a high-spin (quintet) excited state, and the quintet state can be made long lived at low temperatures, typically below 30–50 K.^6,7^ It is a great challenge for molecular engineering to rationally modify such complexes to achieve similar stability close to room temperature. A crucial aspect for future applications is that this family of rather stable molecules is based on the abundant, cheap, and environmentally compatible iron.

Theoretical chemistry-based ligand design can be a great asset for such efforts, yet high-level wave function-based computations are too expensive for applying them routinely to systems as large as transition-metal complexes. However, DFT offers a viable alternative, and the recent developments in understanding and designing certain properties of similar complexes computationally^8−17^ as well as the vast accumulated knowledge on the Fe(II) spin state transition systems^12,18−21^ provide us with a great opportunity to use such complexes as a testing ground in evaluating the impact of different ligand modifications. We selected a prototypical Fe(II) polypyridine complex to assess how electronic substitution effects can modify the potential energy landscape, in particular the singlet–quintet energy gap and the transition rate between these states. Together with an experimental verification of the predicted effects, this would contribute to building up a toolbox for efficient theory-based molecular engineering. Therefore, we synthesized the selected novel substituted complexes and also characterized their physicochemical properties in great detail.

An important question is the selection of the most relevant substitution patterns. A few recent studies discussed the influence of various electron-donating (ED) and electron-withdrawing (EW) groups on the electronic structure of different Fe(II) complexes of pyridine (Py)-bearing ligands and found that substitution at the γ position 4 on the Py ring is rather effective to tune the quintet–singlet energy gap Δ*E*_HL_.^22−25^

Recently, we have also demonstrated that it is possible to control the potential energy landscape of the [Fe(terpy)_2_]^2+^ complexes (terpy = 2,2′:6′,2″-terpyridine) substituted at position 4′ (γ in the central Py ring) with groups of different EW/ED properties, which allowed us to tune the quintet lifetime. According to DFT calculations, a 300 meV (ca. 7 kcal/mol) alteration of the quintet energy was achieved at the extremes, which resulted in a 20-fold difference in the quintet lifetime.^26^ This defines the approximate range in which the properties are tunable with substitutions on the molecular axis of this complex.

As an attempt to explore the opportunities to move beyond the above limits of the substitution at the 4′ position, here we examine how a single substitution at different positions of the terpy ligand affects the electronic structure of the complex. We decided to select a single atom as the substituent to minimize the possible steric effects, allowing us to focus on the variations of the electronic structure. Cl has been chosen for its dual character toward electron withdrawing and donating: first, it has a pronounced −I inductive effect due to its high electronegativity. Second, having lone pairs, the Cl atom can also donate electron density via the +M mesomeric (resonance) effect. Therefore, with a Cl atom at the γ or the α position to the N atom of a Py ring, the N atom should experience larger electron density. These effects are moderate for the case of a Cl substituent, but they are of sufficient magnitude for a systematic study. We have calculated the properties of homoleptic Fe(II) bis-terpyridine complexes having a single Cl substituent per ligand in each case, varying its position to cover all symmetrically unique ones, to explore at which position it has the largest impact on the electronic structure. Having identified this as position 5 (β position with respect to N_eq_, the N atom of the side ring), we extended the study to single and double substitutions with Cl at the 5 and 5′′ positions (on one or both of the side Py rings). Moreover, to confirm these substitution effects and examine their possible combination with the previously described axial (4′) ED/EW tuning,^26^ we have also included in this work the analogous derivatives of the [Fe(4′-SMe-terpy)_2_]^2+^ complex. With respect to molecular switches, the most interesting modification of the potential energy landscape includes the change of the barrier between the excited quintet state and the singlet ground state, which determines the quintet lifetime after photoexcitation. These quintet lifetimes have been determined experimentally by transient optical absorption spectroscopy, and they are compared to the theoretical expectations.

In the main body of the paper we describe how we synthesized novel complexes belonging to the important family of low-spin Fe(II) polypyridines, characterized their physical properties, used DFT to calculate their electronic structure, and compared our experimental and theoretical findings. The present work demonstrates rational molecular design for a well-known family of inorganic compounds, and the toolset used here can be extended to other fields of inorganic chemistry including but not restricted to catalysis, electrochemistry, and photochemistry.

## Experimental Section

### Computations

Molecular properties were calculated with density functional theory (DFT) as implemented in the ORCA 3.0 program package.^27^ Molecular structures were obtained by tight optimization using the TZVP basis set and BP86 functional,^28,29^ which is known to provide good agreement with experiments.^30,31^ Further geometry optimizations were performed with the same basis and the B3LYP* hybrid functional^32−35^ to obtain energies of the singlet, triplet, and quintet states, since this functional had been found to predict fair energies for the different spin states in Fe(II) complexes.^19,20,36−42^ Minimum energy crossing points (MECPs) between the singlet and the quintet potential wells were also located^43^ at the B3LYP*/TZVP level to determine the quintet-to-singlet energy barriers. MECP calculations starting from the two different minima involved converged to the same location and energy.

Theoretical UV–vis spectra were obtained with time-dependent DFT (TD-DFT) at the Tamm–Dancoff approximation (TDA)^44−47^ using B3LYP*/TZVP. For the displayed figures, the transition energies obtained were broadened by a pseudo-Voight function (with Lorentzian and Gaussian fwhm’s of 540 and 600 cm^–1^, except for the region between ca. 330 and 470 nm, where an extra 50% broadening was applied to better match the experiment). Mössbauer parameters were calculated according to the method described in ref (48). The RIJCOSX approximation was used in all B3LYP* calculations to reduce the computational costs. All of our computations utilized the conductor-like screening model (COSMO) to approximate the effect of solvation.

### Materials

**Scheme 1:**
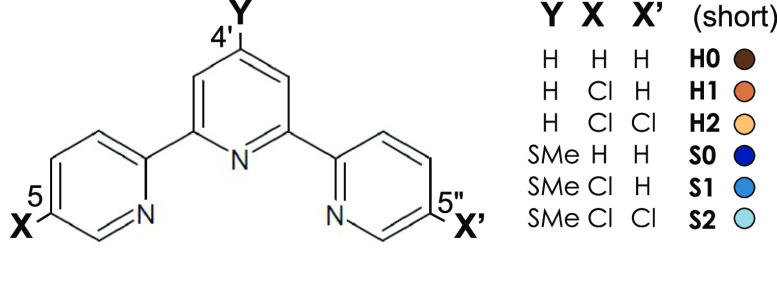
Ligands with Single- and Double-Cl Substitution in the 5 or 5,5′′ Position(s) of the terpy and 4′-SMe-terpy Ligands On the right, a shorthand notation and a color code is shown for the different homoleptic Fe(II) complexes of these ligands.

2,2′:6′,2″-Terpyridine was purchased from Sigma-Aldrich; the other derivatives were obtained from EVOBlocks Ltd., Budapest, in 99+% purity. (The schemes for the ligand synthesis reactions are available in the Supporting Information.) The notations of the samples studied experimentally are defined in Scheme 1. Note that all of these terpyridine derivatives are poisonous and should be treated accordingly. (Nevertheless, their Fe(II) complexes are mostly harmless.) Methanol and acetonitrile were purchased from Molar Chemicals. Toluene, NH_4_PF_6_, Amberlite IRA-402, and Celite 545 were purchased from Sigma-Aldrich.

Preparation of all complexes was carried out using standard methods, with differing solvents used in certain cases. TLC test of the products was performed on a silica plate, where the eluent used was a 4:1:1 mixture of acetonitrile, methanol, and water, saturated with KNO_3_. The tests have shown that–except for the [Fe(5,5″-di-Cl-terpy)_2_]^2+^ (**H2**) compound–the product was clean, containing the complex and a tiny amount of excess ligand which was deemed acceptable for the measurements. In the case of the **H2** complex, further purification was required.

^1^H and ^13^C NMR spectra were recorded on a Varian NMR System 400 spectrometer in D_2_O at 298 K on an indirect detection Z probe except for complex [Fe(5-Cl-4′-SMe-terpy)_2_]^2+^ (**S1**), where a 600 MHz spectrometer was used. The ^1^H chemical shifts are referenced to the residual solvent signal (4.79 ppm). The elemental composition of the samples were determined with HPLC-HRMS at Servier Hungária Ltd., using a Kinetex XB-C18 (50 mm × 2.1 mm, 2.6 μm) chromatography column and a LC118-TOF high-resolution mass spectrometer.

**H0** ([Fe(terpy)_2_]Cl_2_) and **S0** ([Fe(4′-SMe-terpy)_2_]Cl_2_): The preparation method of these complexes is described in ref (26).

4′-H (**H0**): ^1^H NMR (400 MHz, 298 K, D_2_O) δ[ppm] 8.96 (d, *J* = 8.0 Hz, 1H), 8.69 (t, *J* = 8.1, 8.1 Hz, 1H), 8.50 (dt, *J* = 7.8, 1.0, 1.0 Hz, 1H), 7.86 (td, *J* = 8.0, 7.8, 1.4 Hz, 1H), 7.16 (ddd, *J* = 5.6, 1.4, 0.8 Hz, 1H), 7.08–7.00 (m, 1H). HRMS (ESI-TOF) *m*/*z*: M^2+^ calcd for C_30_H_22_N_6_Fe 261.0628; found 261.0626.

4′-SMe (**S0**): ^1^H NMR (400 MHz, 298 K, D_2_O) δ[ppm] 8.77 (s, 2H), 8.47 (d, *J* = 8.0 Hz, 2H), 7.86 (td, *J* = 7.9, 7.8, 1.5 Hz, 2H), 7.24–7.18 (m, 2H), 7.06 (ddd, *J* = 6.7, 5.4, 1.1 Hz, 2H), 3.00 (s, 3H). HRMS (ESI-TOF) *m*/*z*: M^2+^ calcd for C_32_H_26_N_6_S_2_Fe 307.0505; found 307.0505

**H1** ([Fe(5-Cl-terpy)_2_]Cl_2_): 5-Chloro-2,2′:6′,2″-terpyridine (0.200 g, 0.748 mmol) was added to 50 mL of water. The ligand does not dissolve completely; it produces a dispersion. FeCl_2_·4H_2_O (0.071 g, 0.355 mmol) was dissolved in 5 mL of water and added to the ligand dispersion. The mixture was stirred for 12 h at room temperature, after which it was filtered on a G4 glass frit funnel with Celite to remove excess ligand. The solvent was evaporated, and the solid product was dried at 50 °C. The products are purple crystals (0.219 g, 0.332 mmol, 93% yield). ^1^H NMR δ[ppm] 8.96 (dd, *J* = 8.1, 3.5 Hz, 2H), 8.72 (t, *J* = 7.9, 7.9 Hz, 1H), 8.50 (d, *J* = 8.2 Hz, 2H), 7.98–7.84 (m, 2H), 7.16–7.03 (m, 3H). HRMS (ESI-TOF) *m*/*z*: M^2+^ calcd for C_30_H_20_N_6_FeCl_2_ 295.0238; found 295.0238.

**H2** ([Fe(5,5″-diCl-terpy)_2_]Cl_2_): 5,5″-Dichloro-2,2′:6′,2″-terpyridine (0.228 g, 0.753 mmol) was dissolved in 30 mL of acetone. FeCl_2_·4H_2_O (0.073 g, 0.367 mmol) was dissolved in 10 mL of acetone and added to the ligand dispersion. The mixture was stirred for 24 h at room temperature, filtered like **H1**, and dried at 40 °C. The products are purple crystals. TLC tests have shown that the product contains a significant amount of residual FeCl_2_. Purification was carried out by precipitating the [Fe(5,5″-diCl-terpy)_2_](PF_6_)_2_ in water by the addition of NH_4_PF_6_ in great excess. The resulting complex was removed by filtration on a G4 glass frit funnel, washed with water, and dissolved in acetonitrile to remove it from the frit. The acetonitrile was evaporated at 40 °C to get the product (0.280 g, 0.295 mmol, 78% yield). The [Fe(5,5″-diCl-terpy)_2_](PF_6_)_2_ (0.063 g, 0.066 mmol) was dissolved in acetonitrile, and the counterion was exchanged back to Cl^–^ by the use of Amberlite IRA 402 ion-exchange resin in a chromatography column. The product was washed from the column with methanol. The solvent was evaporated, and the solid product was dried at 40 °C (0.034 g, 0.047 mmol, 71% yield). ^1^H NMR δ[ppm] 8.97 (d, *J* = 8.2 Hz, 1H), 8.74 (t, *J* = 8.1 Hz, 0H), 8.49 (d, *J* = 8.7 Hz, 1H), 7.96 (dd, *J* = 8.7, 2.1 Hz, 1H), 7.02 (d, *J* = 2.1 Hz, 1H).

**S1** ([Fe(5-Cl-4′-SMe-terpy)_2_]Cl_2_): 5-Chloro-4′-methyltio-[2,2′:6′,2″-terpyridine] (0.300 g, 0.956 mmol) was added to 75 mL of a 2:1 mixture of water and methanol. The ligand does not dissolve completely in this mixture; it produces a dispersion. FeCl_2_·4H_2_O (0.090 g, 0.453 mmol) was dissolved in 5 mL of methanol and added to the ligand dispersion. The mixture was stirred for 48 h at room temperature and filtered like **H1**, the solvent was evaporated, and the solid product was dried at 35 °C. The products are purple crystals (0.254 g, 0.337 mmol, 74% yield). ^1^H NMR (600 MHz, 298 K, D_2_O): δ[ppm] 8.80–8.76 (m, 2H), 8.47 (d, *J* = 8.7 Hz, 2H), 7.94 (dd, *J* = 8.6, 2.2 Hz, 1H), 7.89 (td, *J* = 7.8, 7.8, 1.5 Hz, 1H), 7.19–7.12 (m, 2H), 7.09 (ddd, *J* = 7.2, 5.6, 1.3 Hz, 1H), 3.01 (s, 3H). HRMS (ESI-TOF) *m*/*z*: M^2+^ calcd for C_32_H_24_N_6_S_2_FeCl_2_ 341.0115; found 341.0117.

**S2** ([Fe(5,5′′-diCl-4′-SMe-terpy)_2_]Cl_2_): 5,5″-Dichloro-4′-methyltio-[2,2′:6′,2″-terpyridine] (0.506 g, 1.45 mmol) was dissolved in 50 mL of toluene. FeCl_2_·4H_2_O (0.141 g, 0.709 mmol) was dissolved in 50 mL of methanol and added to the ligand solution. The mixture was stirred for 24 h at room temperature and filtered like **H1**, the solvent was evaporated, and the solid product was dried at 35 °C. The products are purple crystals (0.286 g, 0.347 mmol, 49% yield). ^1^H NMR: δ[ppm] 8.78 (s, 1H), 8.47 (d, *J* = 8.6 Hz, 1H), 7.96 (d, *J* = 8.6 Hz, 1H), 7.08 (s, 1H), 3.01 (s, 2H). HRMS (ESI-TOF) *m*/*z*: M^2+^ calcd for C_32_H_22_N_6_S_2_FeCl_4_ 374.9725; found 374.9723.

UV–vis spectra were taken with a Jasco V-750 UV–visible spectrophotometer; molar absorption coefficients were calculated from the data using the concentration determined from the actual Fe content obtained with ICP-MS measurements.

^57^Fe Mössbauer spectroscopy data were taken at 294 K with a Wissel spectrometer using a sinusoidal velocity profile. Calibration was performed with a 21.1 μm thick high-purity α-iron reference foil. Isomer shifts are given relative to α-Fe, and the spectra were analyzed with the MossWinn 4.0 program.^49^

Cyclic voltammetry (CV) measurements were performed in a standard three-electrode arrangement (Pt working and counter electrodes and a Ag/AgNO_3_ reference electrode) connected to a BioLogic VMP-300 potentiostat. The complexes were used as PF_6_^–^ salts dissolved in dry acetonitrile (Sigma-Aldrich) also containing 0.1 M tetrabutylammonium hexafluorophosphate ([^*t*^Bu_4_N][PF_6_], Sigma-Aldrich) as supporting electrolyte. The stock solutions were dried over 3 Å zeolite beads (Alfa Aesar, 3–5 mm) for at least 24 h. The concentration of the solutions was around 1–2 mM, and all measurements were performed in a water- and oxygen-free environment secured by a continuous flow of 5.0 grade Ar gas saturated with acetonitrile before and during the measurement. The CVs were recorded at a scan rate of 100 mV/s, and the oxidation and reduction potential values were determined as the average of the corresponding cathodic and anodic waves. All reported potential values are the *E*_1/2_ average potentials, referred to the ferrocene/ferrocenium redox couple used as the internal reference.

Single-crystal X-ray diffraction measurements were performed on an Agilent Supernova diffractometer equipped with a dual-microfocus source, kappa goniometer, position-sensitive detector, and dry nitrogen gas flow cooler. Data were taken at 295 and 100 K using the Cu or Mo source in a hemisphere of the reciprocal space up to 0.8 Å resolution. The structure solution and refinement were done with SHELX^50^ software called from either scripts via the command line or from Olex2.^51^

Femtosecond transient absorption spectra were measured by the UV pump–supercontinuum probe technique using a home-built spectrometer based on an amplified Ti:sapphire laser system, described in detail in our previous communication.^26^ Part of the 800 nm Ti:sapphire beam (1 mJ, 50 fs, 1 kHz repetition rate) was frequency doubled in a β-barium borate (BBO) crystal, chopped at 500 Hz, and used as the pump. A portion of the 800 nm beam was delayed with a linear translation stage and used for supercontinuum generation in a sapphire crystal. The supercontinuum probe beam, spanning the 430–750 nm wavelength range, was further split into signal and reference beams that were spectrally dispersed and registered shot-to-shot with two CCD sensors. The instrument response function was less than 100 fs (fwhm) over the entire spectrum. The data were averaged over 5–9 scans to improve the signal-to-noise ratio. Measurement control was realized by in-house written software. Routines for data preprocessing (coherent artifact and GVD correction) and analysis were developed in our laboratory.

## Results and Discussion

### Substitution Effects with One Cl at Different Positions

**Figure 1:**
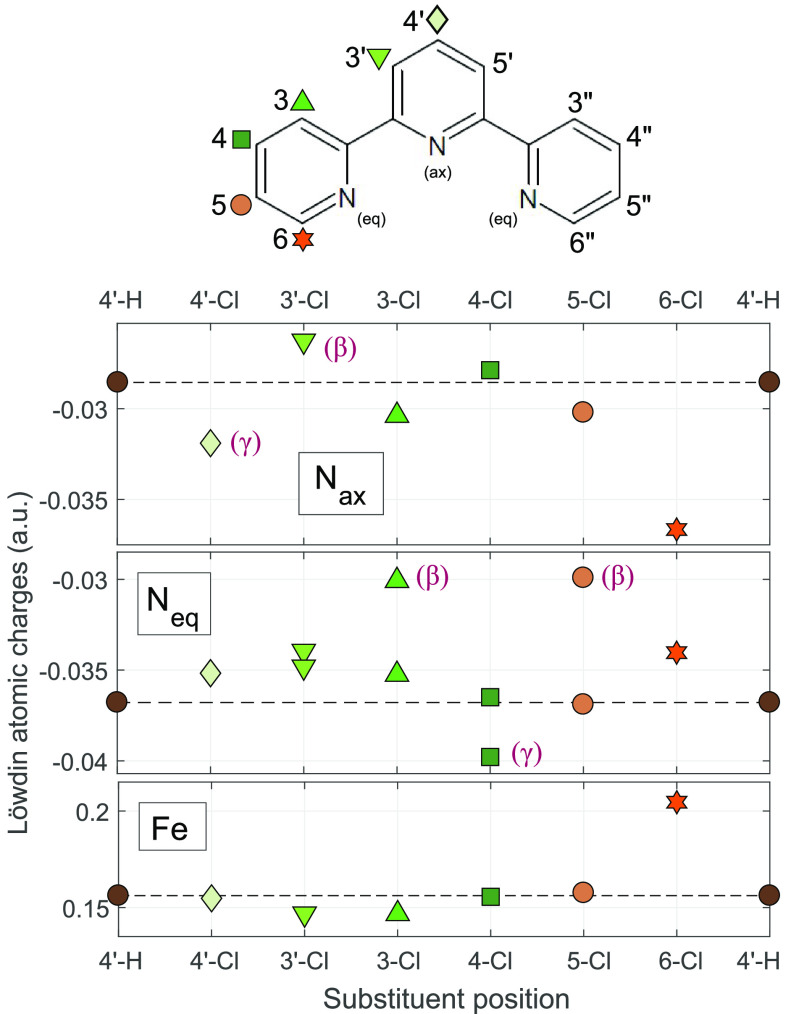
Calculated Löwdin total atomic charges (B3LYP*/TZVP) at the N atoms (middle panels) and at the Fe (bottom) in the ground state of the Fe(II) complexes with terpy ligands substituted with a single Cl atom in different positions. Substituent positions of the terpy are indicated on the abscissa; the symbols correspond to the notations of position indicated in the top. 4′-H (dark brown) denotes values that belong to the Fe(II) complex of the unsubstituted terpy, which serves as a reference. The charge on the N_eq_(Cl) in the α-substituted Py ring (6-Cl) is not shown in the figure because it has a very low value compared to the others: −0.077 au. Greek letters in parentheses serve as reminders to the relative location of the Cl in the Py ring containing the given N atom and are shown next to the value belonging to such affected N(Cl).

In order to investigate how substitution at different positions affects the properties of the complex, calculations were done on the series of [Fe(terpy)_2_]^2+^ derivatives substituted with a single Cl atom (on both ligands). Six symmetrically different positions are available for substitution on the terpy ligand, which are, from the center to the side, as follows: two on the central ring at positions 4′ and 3′ and four on the side ring in positions 3, 4, 5, and 6 (see the top of Figure 1). From these, the 3′, 3, and 5 are at β (“meta”) positions to the N atom in their Py rings; thus, for these substitutions, the affected N atoms (herein N(Cl)) are expected to experience similar effects, namely, a decrease of the electron density because of the −I effect. Likewise, the cases having a Cl atom at the γ (“para”) positions, such as 4′ or 4, are also expected to show some similarities, but here, it is the +M effect that dominates, increasing the electron density on the affected N atom. Position 6 is an α (“ortho”) position next to the N atom, at which the +M effect should also be relevant; however, such closeness to the N atom is known to weaken the coordinative bond to the metal in complexes caused by the strong sterical effect, leading to a significant weakening of the ligand field.^8,52^ Since this substitution is starkly different from the other cases, we do not examine it as closely as the others but will seize the opportunity to make use of the available data on the Fe(II) complex of the 6,6″-dichloro-terpy ligand^52^ and compare our calculations to the experimental results on this complex of a high-spin (quintet) ground state.

Finally, we note that the side rings can also be considered as substituents of the middle one; thus, variations of the electron density on the linking C atom can also contribute to some extent to the electron density on the N atom in a given Py ring.

However, in order to understand the relevant impacts of the substitution, it is also important to overview the structural changes it causes. The most relevant calculated structural parameters from the BP86-optimized molecular geometries are listed in Table 1; note that the table also contains data pertinent to the second part of this paper only. (Higher spin state data is given, too, for completeness. The B3LYP*/TZVP-optimized geometries, which are expected to be less accurate but what were used to calculate the electronic structure and energies, can be found in the Supporting Information. The available crystallography data are in agreement with these calculated structures, as we will discuss later.) It is clear from the data that the 6-Cl substitution lengthens the Fe–N bonds, in particular the one to the N_eq_ that is next to the Cl atom that causes the large steric strain. Substitution at position 3 or 3′ also leads to two different Fe–N_eq_ bond lengths, which can be explained by an intraligand steric effect between the middle and the side ring, which pushes the latter closer to the Fe, leading to a smaller distance between the Fe and the N_eq_(Cl), the N atom in the substituted side ring. These effects are also reflected by the NNN angle in the ligands. The rest of the series does not show such structural anomalies, as the Fe–N_eq_ bond length *r*(Fe–N_eq_) and the NNN angle vary very little for those complexes.

**Table 1:** Selected Structural Parameters of the FeN_6_ Core in the BP86/TZVP-Optimized Molecular Geometries for the Lowest Singlet, Triplet, and Quintet States of the Derivatives of the [Fe(terpy)_2_]^2+^ Complex Studied^a^

	singlet			triplet			quintet		
substituents of the terpy ligand	*r*(Fe–N_ax_)	*r*(Fe–N_eq_)	NNN	*r*(Fe–N_ax_)	*r*(Fe–N_eq_)	NNN	*r*(Fe–N_ax_)	*r*(Fe–N_eq_)	NNN
- (terpy, **H0**)	1.886 Å	1.979 Å	102.2°	1.909 Å	2.127 Å	108.1°	2.100 Å	2.193 Å	108.1°
4′-Cl	1.884 Å	1.980 Å	102.3°	1.908 Å	2.127 Å	108.2°	2.099 Å	2.194 Å	108.3°
3′-Cl (=5′-Cl)	1.890 Å	1.975 Å	101.3°	1.918 Å	2.156 Å	106.6°	2.119 Å	2.176 Å	106.5°
		1.963 Å			2.121 Å			2.164 Å	
3-Cl	1.884 Å	1.976 Å	101.9°	1.910 Å	2.095 Å	107.4°	2.106 Å	2.179 Å	107.4°
		1.971 Å			2.128 Å			2.182 Å	
4-Cl	1.886 Å	1.978 Å	102.2°	1.909 Å	2.121 Å	108.5°	2.100 Å	2.189 Å	108.1°
		1.980 Å			2.131 Å			2.196 Å	
5-Cl (**H1**)	1.887 Å	1.979 Å	102.2°	1.910 Å	2.114 Å	108.0°	2.101 Å	2.187 Å	108.1°
		1.980 Å			2.139 Å			2.201 Å	
6-Cl	1.893 Å	1.984 Å	103.8°	1.914 Å	2.088 Å	108.9°	2.138 Å	2.195 Å	108.5°
		2.064 Å			2.232 Å			2.243 Å	
6,6″-diCl	1.891 Å	2.074 Å	105.9°	1.913 Å	2.218 Å	111.1°	2.080 Å	2.301 Å	112.0°
**H2** (5,5″-di-Cl)	1.889 Å	1.980 Å	102.2°	1.911 Å	2.128 Å	108.1°	2.096 Å	2.198 Å	108.3°
**S0** (4′-SMe)	1.886 Å	1.980 Å	102.4°	1.909 Å	2.130 Å	108.4°	2.130 Å	2.185 Å	107.8°
**S1** (5-Cl-4′-SMe)	1.888 Å	1.979 Å	102.4°	1.912 Å	2.117 Å	108.4°	2.125 Å	2.187 Å	107.9°
		1.980 Å			2.141 Å			2.190 Å	
**S2** (5,5″-di-Cl-4′-SMe)	1.889 Å	1.980 Å	102.4°	1.912 Å	2.131 Å	108.4°	2.121 Å	2.190 Å	108.1°

a NNN is the N_eq_N_ax_N_eq_′ angle in a ligand. In complexes of single-Cl-substituted asymmetric ligands, when two different *r*(Fe–N_eq_) bond lengths are seen, the second (lower) one stands for the distance between the N_eq_ directly affected by the Cl substituent (i.e., the Cl is on the same ring, or in case of 3′-Cl substitution, closer to the given Py ring).

The variations of the electron density should be reflected by the Löwdin total atomic charges to an accuracy sufficient to evaluate the impact of the substitutions; therefore, these are displayed for the N and Fe atoms in Figure 1 along the substitution position in the series. As it is apparent from the figure, the atomic charges indeed vary according to the expectations discussed above.

Starting their description with N_ax_, the two possible substitutions on the middle ring result in opposite effects. In the case of the 4′ substitution, the +M effect leads to a pronounced increase in the electron density of the N_ax_ compared to [Fe(terpy)_2_]^2+^: thus, its Löwdin atomic charge becomes more negative. The other available position on the middle ring is 3′, a β position to N_ax_; therefore, only the −I effect prevails, leading to a decrease of the electron density and thus an increase in the charge on N_ax_. For the remaining positions (except 6-Cl) the side ring substitution causes smaller differences in the charge on N_ax_; these stem from the α +M and −I substituent effects of the side rings as well as structural strains in certain cases. Considering the charge on the N_eq_, the position 4 γ substitution causes an effect very similar to that seen with the γ in the middle ring. The two β substitutions on the side ring, at positions 3 and 5, cause a decrease of the electron density on N_eq_(Cl) due to the −I effect that is larger than that in the case of the middle ring (3′-Cl), probably because the interaction between the N and the Fe atoms is smaller due to the larger Fe–N_eq_ distance. The far N_eq_ in the other side ring is either unaffected (5-Cl) or slightly affected due to the steric interaction (3-Cl). The substitution on the middle ring changes the charge on the N_eq_ only moderately, again via (previously discussed) steric and side ring substituent effects. For completeness, the total charge on the Fe atom is also shown in Figure 1. It is obvious from the figure that large differences are only observed in cases with steric distortions.

Finally, the Cl in position 6 occupies an α position to the N_eq_ atom of the side ring and the meta position to the central ring, which should lead to similar ED/EW effects as substitution in position 4. However, here, the steric interaction between the Cl and the Fe is very big, which weakens the Fe–N_eq_(Cl) bond, and the Löwdin charges (Fe, 0.2046 au; N_eq_(Cl), −0.0768 au) readily reflect the resulting smaller overlap between these atoms. (The value for N_eq_(Cl) is out of the displayed range in Figure 1, as showing it would squeeze the scale and render the variations of N_eq_ difficult to read.)

We can infer from the above that the variation of the atomic charges presented reveal that apart from the 6-Cl substitution with its extreme steric interaction, all other substitutions cause a smaller change in the electron density of the N_ax_ than the previously studied substitution at position 4′. The larger changes in the charge of the Fe are also connected to distortions by steric interactions. The charge density of the N_eq_ atom shows interesting variations in the remaining cases, i.e., for the side ring substitutions at positions 3, 4, and 5. From these, the latter two are free from steric effects, and the variation of the N_eq_ atomic charges are of different sign and size, the 5-Cl being the larger one.

Thus, we can conclude that for going beyond the substitution at position 4′, position 5 seems to be the most interesting one from the above series, since the electronic effects on the N_eq_ are the largest, but steric effects do not pose any complications. As we will see later at the discussion of the high-spin states, the energy of the quintet state justifies this choice because it is the second deepest for substitution at position 5 (after position 6). Therefore, we decided to perform a detailed combined computational and experimental study on complexes with this type of substitution pattern, in particular, attention to the quintet lifetime, which is described in the rest of this paper.

### Complexes Substituted at the 5 (and 5′′) Position(s) with Cl

In order to better understand how substitution at position 5 varies the properties of the complex, we have extended our examination to include the complex of the 5,5″-di-Cl-terpy ligand (**H2**), which has a second Cl in the analogous β position of the other side ring. In addition, we examined another series of similarly modified [Fe(terpy)_2_]^2+^ derivatives, which were substituted simultaneously in the 4′ position with an electron-donating SMe group to verify the side ring β-substitution effect as well as to inspect the combined impact of the parallel substitution in the 5 and the 4′ positions on the electronic and molecular properties. (The effect of the 4′-SMe substitution was studied previously in ref (26).) Overall, two series of three complexes, whose ligands are displayed in Scheme 1, were thoroughly tested theoretically and experimentally.

### Ground State Properties and Substitution Effects: Structure and Charge Distribution

First, we explore the impact of the substitution on the structure of the ground state of the 5 and 5,5′′ complexes crucial to the understanding of the later discussion of the electronic structure.

**Figure 2:**
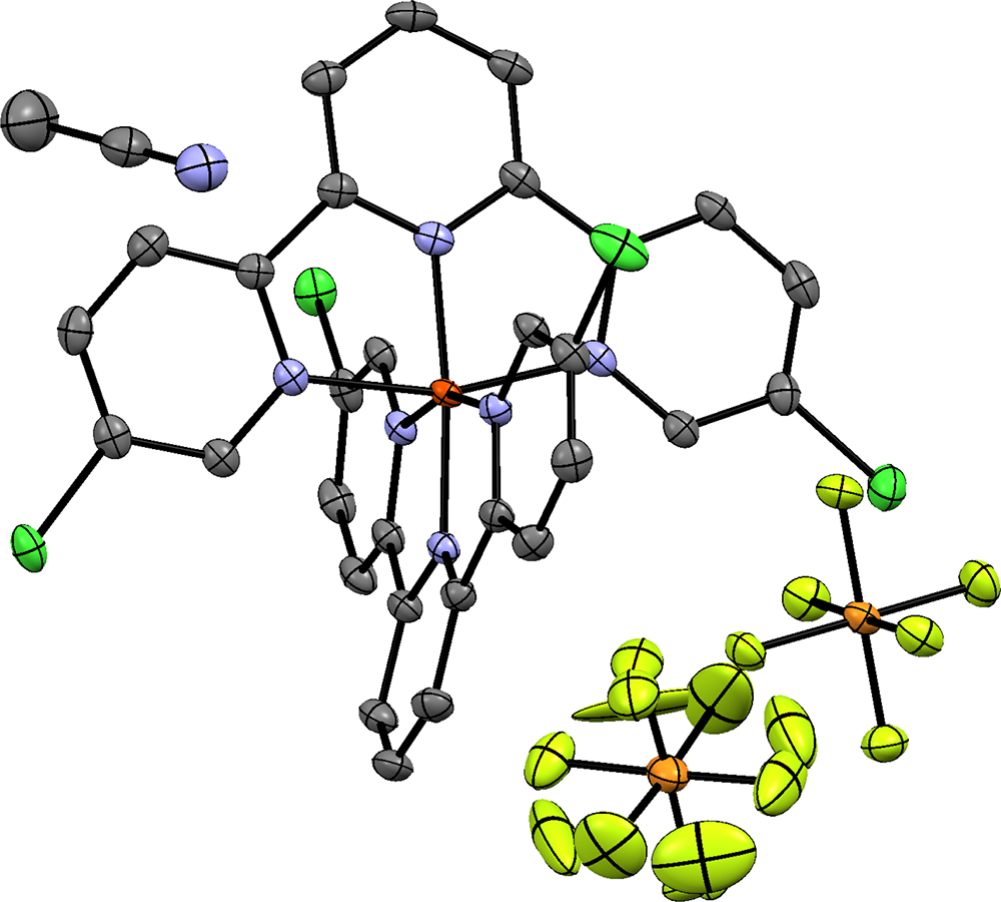
ORTEP diagram of the (PF_6_)^−^ salt of **H2**, without showing the hydrogen atoms. The data was taken at 100 K. A molecule of the crystalline solvent acetonitrile is also seen.

According to our calculations (Table 1), no significant structural changes occur in the FeN_6_ core or in the entire [Fe(terpy)_2_]^2+^ frame that reflect the substitutions with Cl at positions 5 and 5′′. The two-step substitution implies monotonic changes around 0.001–0.003 Å in *r*(Fe–N_ax_),  and this is the largest effect that occurs. With the single substitution at position 5, the two *r*(Fe–N_eq_) become slightly different, but their difference is smaller than 0.001 Å and only appears in their values in Table 1 because of the rounding. Simultaneously, the NNN bite angles (N_eq_–N_ax_–N_eq_′ angles) display about 0.01–0.03° change for both the **Hx** and the **Sx** series. These are in agreement with the structural data obtained via XRD measurements, which are presented in Table 2. (Unfortunately, none of the crystals grown from the singly substituted compounds **H1** and **S1** were of suitable quality for crystallography.) The bond lengths in each compound display tiny and nonsystematic variations: *r*(Fe–N_ax_) and *r*(Fe–N_eq_) vary within a 0.005 Å range around 1.885 and 1.985 Å, respectively, while the N–N–N angle varies between 102.2° and 102.8°. It is worth noting that literature structural data for the singlet terpy and the quintet 6,6″-dichloro-terpy complexes of Fe(II) also compare well with the calculations. The XRD results also show a small nonzero rocking angle Θ (= 180° – N_ax_FeN_ax_′), which is not present in the DFT results. This difference arises from solid state effects in the crystal (while the geometry optimization was performed on single complex molecules). By examining the molecular structures (provided in the Supporting Information), it becomes obvious that the large (PF_6_)^−^ ions make some of the middle Py rings tilted. This tilting is clearly seen in the projection of the structure of the dichloro-substituted complex **H2** determined by crystallography in Figure 2. The packing patterns cause the tilting of every second ligand, one for each complex molecule in the (PF_6_)^−^ salts of **H2** and **S0**, but in **S2**, both ligands are similarly affected. However, it is also clear from the structures that the FeN_6_ core is not perturbed strongly. Overall, the theoretical and experimental structures agree fairly well, which again reinforces the use of BP86 when calculating the molecular geometry of Fe(II) compounds.

**Table 2:** Comparison of Selected Calculated (BP86/TZVP) and Experimental Structural Parameters^a^

substituents	*r*(Fe–N_ax_)	*r*(Fe–N_eq_)	NNN	Θ
**H0** (calcd)	1.886 Å	1.979 Å	102.2°	0.0°
exp. (ref ^53^)	1.890(4) Å	1.988(3) Å	102.8(2)°	1.5(3)°
**H2** (calcd)	1.889 Å	1.980 Å	102.2°	0.1°
exp., *T* = 295 K	1.890(2) Å	1.986(2) Å	102.4(1)°	5.3(1)°
exp., *T* = 100 K	1.890(1) Å	1.983(3) Å	102.2(1)°	5.6(1)°
**S0** (calcd)	1.886 Å	1.980 Å	102.4°	0.1°
exp., *T* = 295 K	1.882(2) Å	1.976(4) Å	102.2(2)°	2.6(1)°
**S2** (calcd)	1.889 Å	1.980 Å	102.4°	0.1°
exp., *T* = 295 K	1.888(3) Å	1.990(7) Å	102.6(2)°	4.7(2)°
exp., *T* = 100 K	1.883(2) Å	1.976(6) Å	102.2(1)°	4.4(1)°
6,6″-diCl (calcd)	2.080 Å	2.301 Å	112.0°	0.0°
exp. (ref ^52^)	2.080(2) Å	2.272(1) Å	111.4(1)°	4.1(1)°

a Θ is the rocking angle 180° – N_ax_FeN_ax_′. The experimental values, where reference is not indicated, are from this work. Note that the ground state of the complex with the 6,6″-diCl-terpy ligand is the quintet.

**Figure 3:**
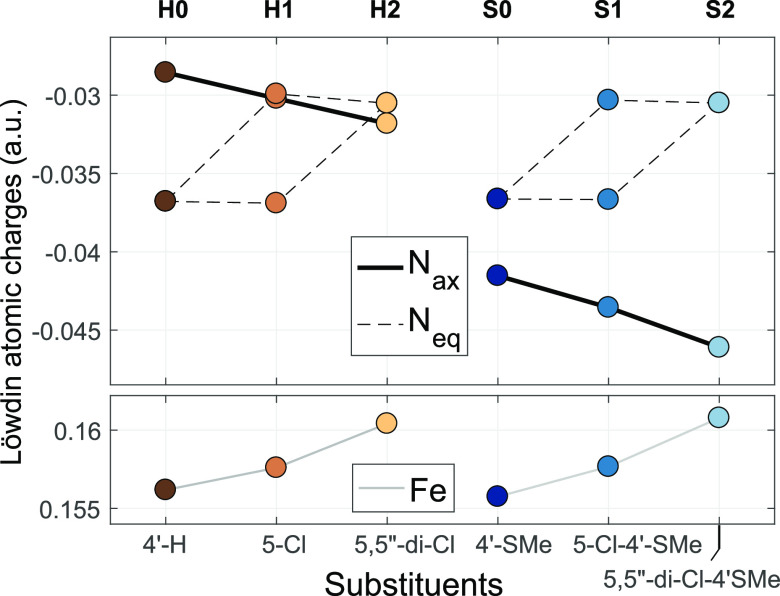
Variation of the (B3LYP*/TZVP) Löwdin atomic charges on the six N atoms (top) and the Fe (bottom) in the ground state of the complexes with single- and double-Cl substitution in the 5 or 5,5′′ position(s) of the terpy and the 4′-SMe-terpy ligands, respectively.

We continue with examining the relevant atomic charges in the **Hx** and **Sx** series, similarly to the previous section. The total Löwdin atomic charges for the FeN_6_ core are displayed in Figure 3. As we saw before, the single Cl substitution at position 5 in **H1** results in a lower electron density on the affected N_eq_ atom (because of the −I effect of the β Cl). It is evident from the figure that adding a second Cl to the other side ring of [Fe(terpy)_2_]^2+^ at the equivalent position 5′′ removes the difference between the N_eq_ charges, as the electron density is then decreased equally on both of these N atoms. The same applies to the [Fe(4′-SMe-terpy)_2_]^2+^ series, where a similar difference builds up between the substituted N_eq_(Cl) and the other N_eq_ at single substitution (**S1**), which also disappears at the 5,5′′ double substitution (**S2**). By comparing the atomic charges between the two series of complexes, it is apparent that 4′-SMe substitution leaves the side rings unaffected, as in both the [Fe(terpy)_2_]^2+^ and the [Fe(4′-SMe-terpy)_2_]^2+^ series the atomic charges of the N_eq_ are practically identical. On the other hand, the charge of the N_ax_ differs significantly in the 4′-H and 4′-SMe systems due to the electron-donating nature of the SMe substituent in the γ position in the middle Py ring, and thus, it leads to an increase in the electron density on N_ax_ via the +M effect in the **Sx** compounds. Beside this, the total atomic charge on the N_ax_ in the **Hx** and the **Sx** complexes varies quite similarly: it increases gradually upon the single (5) and double (5,5′′) substitutions. Since the Cl at position 5 of the side ring is in fact in a para position to the central ring and since on the latter the side ring is at an α position (2′), the +M effect of the Cl can lead to a transfer of electron density to N_ax_. Therefore, the substituted side ring can act as a weakly electron-donating substituent to the central ring. The charge on the Fe is also systematically influenced by these substitutions. However, the 4′-SMe substitution has a rather subtle effect on the total electron density of the Fe: there is almost no difference between the **Hx** and the **Sx** compounds.

We emphasize that the above variations of the atomic charges are rather small. However, Mössbauer spectroscopy, which is very sensitive to the symmetry of the charge distribution around the Fe nucleus, is able to distinguish the minuscule substitution effects in the ground state. In particular, it clearly shows the variation of the electric field gradient (EFG) at the Fe, which in this case stems from the difference of the electron density along the main molecular axis and those perpendicular to it. This asymmetric charge distribution, interacting with the quadrupole moment of the ^57^Fe nucleus, gives rise to the quadrupole splitting (Δ) in the Mössbauer spectrum. Since the axial Fe–N bonds are significantly shorter in these complexes than the equatorial ones, the charge distribution will resemble that of an axially compressed octahedron, which leads to a negative sign for the quadrupole splitting, and because these differences are large, the value is around 1 mm s^–1^, which is rather large for a low-spin Fe(II) complex. Replacing H in the axial 4′ position with the electron-donating SMe group causes an increase in the electron density on the molecular axis, which results in the increase of the absolute value of the quadrupole splitting, as seen in Table 3, albeit it is significantly stronger in the calculation than in the experiment. Nevertheless, if we compare the calculated and measured quadrupole splitting values in Table 3, it is obvious that, similarly to the two series, the differences between the complexes upon Cl substitution show systematic variations with the addition of the side ring Cl substituents. The absolute value of the quadrupole splitting shows an increment with each added Cl atom, reflecting the increase of the axial asymmetry of the charge distribution around the iron. This is fully consistent with the variation of the atomic charges of the N atoms, as seen in Figure 3: the axial asymmetry becomes larger when the charge on the N_ax_ atom is more negative and/or when on N_eq_ atom is more positive; this is exactly what happens at the stepwise Cl substitutions. Nevertheless, we wish to emphasize again that these variations are rather small. (The isomer shift values, which are related to the total charge at the iron nucleus, are also given for completeness, but their small variation, the relatively large scatter in the experimental values, and the complications arising from the opposite effects of the s and d electrons do not allow us to draw meaningful conclusions from this current data set.)

**Table 3:** Calculated and Measured Mössbauer Parameters of the Studied Complexes^a^

complex	δ_calcd_ (mm/s)	Δ_calcd_ (mm/s)	δ_exp_ (mm/s)	|Δ_exp_| (mm/s)
**H0**	0.329	–0.936	0.207(2)	1.070(4)
**H1**	0.337	–0.999	0.184(1)	1.112(2)
**H2**	0.346	–1.058	0.223(2)	1.146(5)
**S0**	0.338	–1.042	0.204(1)	1.076(3)
**S1**	0.346	–1.108	0.203(1)	1.158(2)
**S2**	0.355	–1.170	0.208(1)	1.226(1)

a δ: isomer shift. Δ: quadrupole splitting. Data were taken at 298 K on complexes with Cl^–^ counterions except for **H2**, where the counterion was (PF_6_)^−^. The calculated asymmetry parameter η is not shown as it is negligibly small for all complexes (between 0.005 and 0.013).

The systematic variations observed in the charge distribution direct the attention to the electronic structure of these compounds and call for a thorough investigation of the most important orbitals.

### Ground State Orbital Energies and Optical Spectra

The first insight into the variations of the frontier orbitals can be expected from cyclic voltammetry (CV).^54^ The oxidation potential is often considered as a measure of the stabilization of the highest occupied molecular orbital (HOMO); in these type of complexes it is the t_2g_(-like) orbitals.^55^ But, it was also shown that despite some earlier claims, its variations cannot be taken as a measure of the ligand field strength.^24^ The (first) reduction potential gauges the stabilization of the lowest unoccupied molecular orbital (LUMO) in a similar manner. Consequently, these quantities allow us to assess the effects of the substitutions on the energies of the HOMO and LUMO and on the gap between them.

The electrochemical properties of the complexes investigated infer some systematic behavior indeed (cf. Table 4). A relevant difference is apparent between the 4′-SMe- and 4′-H-substituted systems, with the 0.1 eV smaller oxidation potential suggesting the destabilization of the t_2g_ orbitals in the **Sx** complexes. However, substitution of a Cl at position 5 on the side ring shifts the oxidation potential higher by 54–58 mV in both cases (**H1** and **S1**). In the complexes with a second Cl on the side rings at position 5′′ there is another +100 mV shift in the oxidation potential both in **H2** and in **S2**. Accordingly, the effect shows a clear, systematic correlation with the Cl substitution with each step stabilizing the HOMO. The first reduction potential also increase monotonically with the Cl substitution. Therefore, the differences in the oxidation and the first reduction potentials are rather similar, suggesting that the HOMO–LUMO gap remains similar in these complexes. The 4′-Cl-terpy complex has also been measured with CV, whose parameters are rather close to those of **H1** despite the differences in the electron-donor behavior of Cl at positions 4′ (+M effect) and 5 (−I effect). This suggests that the effects of the Cl substitutions on the electrochemical properties are mostly electrostatic in nature.

**Table 4:** Oxidation Potential (OX), First (R1) and Second (R2) Reduction Potentials (as determined from CV with respect to ferrocene/ferrocenium as reference), and Difference of the Oxidation and the First Reduction Potentials

complex	OX (V)	R1 (V)	R2 (V)	OX–R1 (V)
**H0**	0.735	–1.641	–1.806	2.376
**H1**	0.789	–1.570	–1.725	2.359
**H2**	0.885	–1.565	n.a.	2.451
**S0**	0.637	–1.658	–1.807	2.295
**S1**	0.695	–1.580	–1.722	2.275
**S2**	0.796	–1.546	n.a.	2.342
4′-Cl	0.768	–1.537	–1.764	2.305

**Figure 4:**
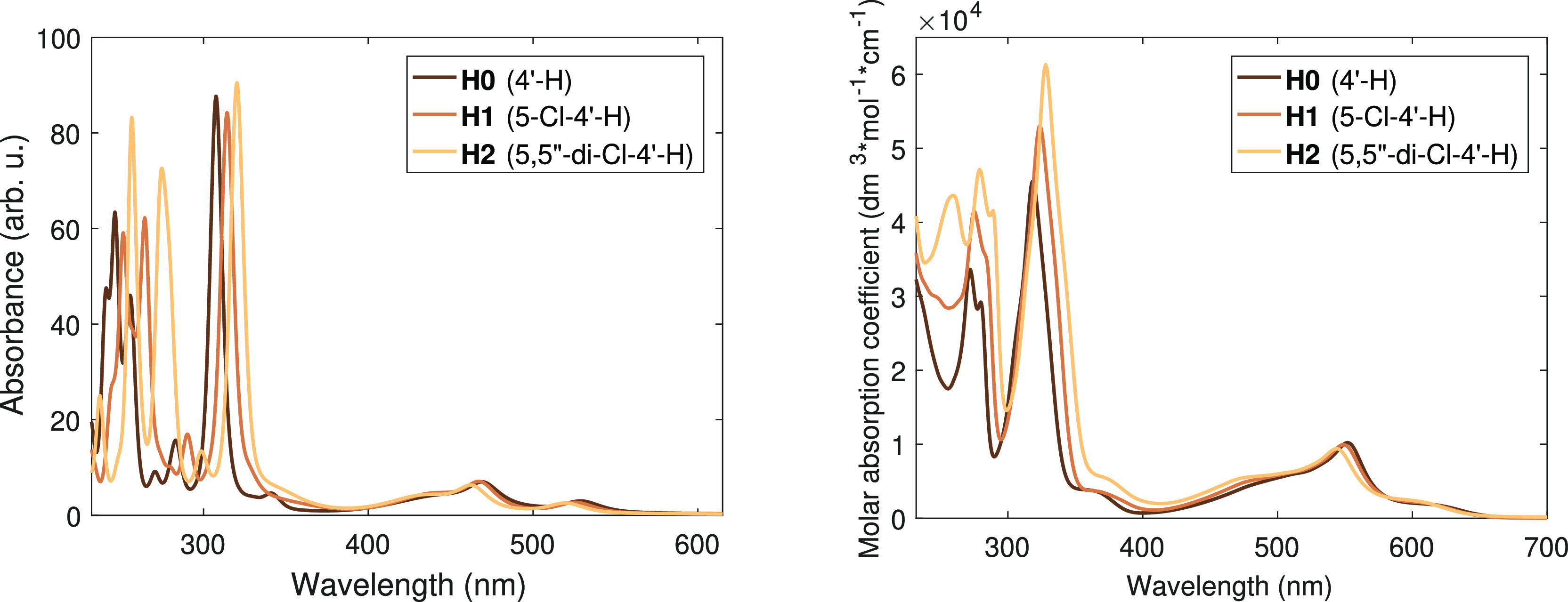
Calculated (left) and experimental (right) UV–vis spectra of the studied derivatives of [Fe(terpy)_2_]^2+^.

**Figure 5:**
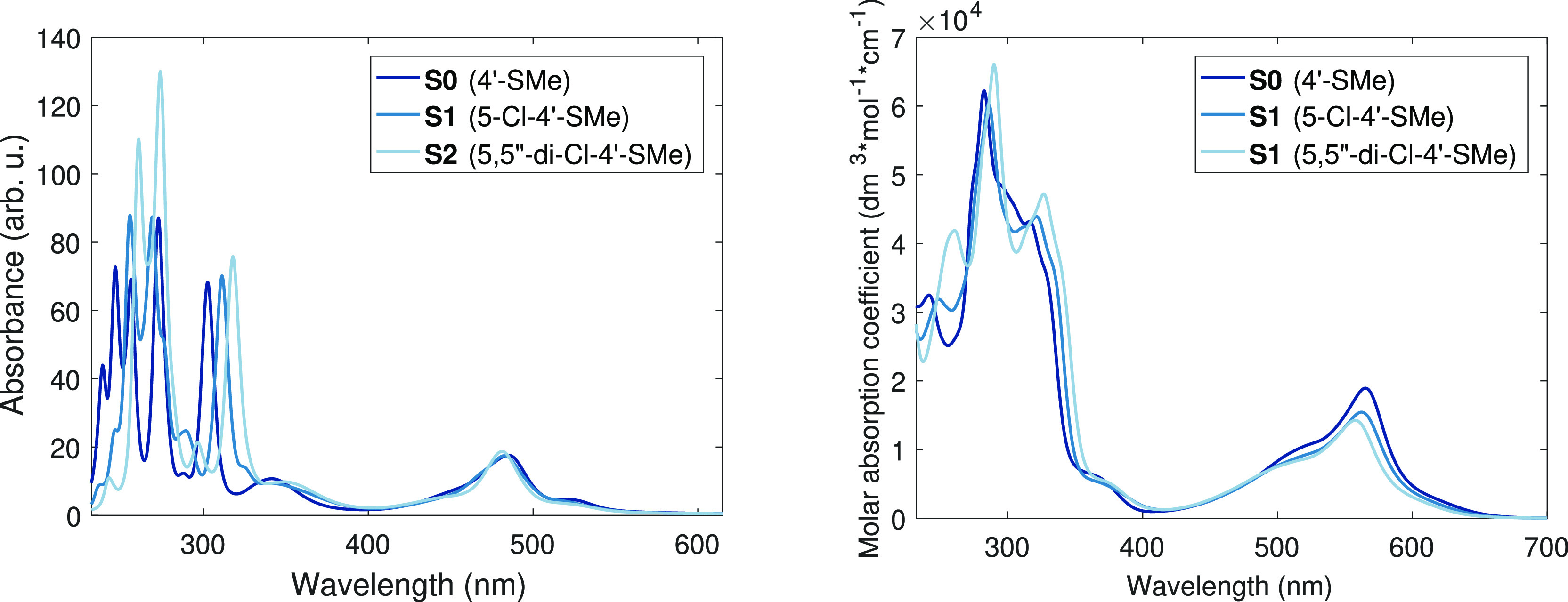
Calculated (left) and experimental (right) UV–vis spectra of the studied derivatives of [Fe(4′-SMe-terpy)_2_]^2+^. The legend describes the substituents of the base terpy ligand.

UV–visible spectroscopy combined with results from time-dependent DFT (TD-DFT) calculations can provide us with valuable further information about the electronic structure. Therefore, we have also applied them to investigate the molecular systems studied; the results are displayed in Figures 4 and 5. As it is apparent from these figures, there is a prominent similarity between the calculated and the experimental spectra. (The agreement is surely not quantitative, as the utilized B3LYP* functional overestimates the transition energies in the visible region by approximately 50 nm, causing a hypsochromic (blue) shift, but this is still within the expected accuracy range of the DFT and the TD-DFT methods.)

**Figure 6:**
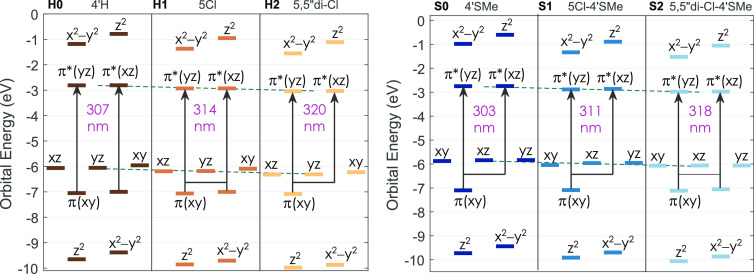
Molecular orbital energy diagram of the derivatives of [Fe(terpy)_2_]^2+^ (left) and the derivatives of [Fe(4′-SMe-terpy)_2_]^2+^ (right). The arrows indicate the origin of the band in the UV spectra that shifts with the Cl substitution. The dashed lines are parallel.

In the visible region (400–700 nm), both the calculated and the experimental spectra look fairly insensitive to the substitution. The analysis of the TD-DFT calculations reveals that in these transitions electron density is transferred from the populated Fe 3d-based t_2g_-like orbitals (one of which is the HOMO in these complexes) to the LUMO, which is a ligand-based π* orbital. (The calculations also indicate some mixing of these Fe 3d and the ligand π-based orbitals; thus, none of these are pure metal or ligand centered. This will be addressed later in more detail.) The energies of these orbitals are presented in Figure 6, which clearly shows that even though the orbital energies decrease within the substitution series, their difference does not change: the inductive effect of the Cl atoms causes the HOMO and LUMO energies to shift in parallel (cf. Figure 6, dashed lines). This is consistent with the cyclic voltammetry results discussed above (Table 4) and confirms that the HOMO–LUMO gap is rather close for all of the complexes investigated.

In contrast to the visible part of the spectrum, in the UV region, the TD-DFT calculations predict systematic red shifts with the 5 (and 5′′) Cl substitutions for both series. This is exactly what is found in the experimental data: in perfect agreement with the calculations, the two-step Cl substitution of the side ring at the β position causes gradual and close to equally spaced red shifts, as it is seen in Figures 4 and 5. This striking behavior impelled us to make efforts to discover the electronic structural reason behind this high sensitivity to the substitution.

Previous studies on Fe(II) complexes with Py-based ligands having an ED(EW) group at position 4 (γ) of the Py ring reported a similar blue(red)—hypsochromic(bathochromic)—shift of bands in the near-UV part of the spectra. This was interpreted as a consequence of the modification of the π-acceptor character of the ligand, whose increase resulted in increased Fe 3d–ligand π* backbonding, leading to a stronger ligand field. Thus, a red shift of the affected π* ← t_2g_ MLCT band was claimed to indicate an increase in the ligand field strength.^22,56^

The agreement between the experiment and the TD-DFT calculations allows us to turn to theory with confidence in order to verify the hypothesis that the systematic red shifts in the 300–350 nm region can be explained by increasing the ligand field strength. Our first observation is that the nature of the transition is somewhat different from that reported before:^22^ it is a π*(*xz*), π*(*yz*) ← π(*xy*) transition. Thus, the electrons do not originate from pure metal-centered t_2g_-like orbitals. (While these transitions involve many states and cannot be rigorously explained in the single-electron picture, selecting the contributing orbitals with the largest weights should give a sufficiently illuminating description.) These π(*xy*) orbital energies are about 1 eV lower than that of the HOMO, and they are composed of ligand π orbitals mixed with Fe 3d_*xy*_ atomic orbitals, the latter having a 5–6% weight in the molecular orbital. The π*(*xz*), π*(*yz*) molecular orbitals are based on the π* ligand orbitals, but also the Fe 3d_*xz*_ and 3d_*yz*_ orbitals are mixed in to a small extent to form the LUMO and LUMO + 1 due to π-backbonding between the metal d orbitals and the ligand π orbitals, see Figure 6. (Note that these mixings are consistent with the description of polypyridine ligands having dual π donor and acceptor characters in Fe(II) complexes, as reported by Ashley and Jakubikova.^24^) However, the origin of the red shifts seems inconsistent with the claim in previous works that it reflects an increase in the ligand field;^22,56^ the latter would suggest that the ligand field becomes larger with the 5(5″-di)-chloro substitution. In fact, the calculations indicate a *decreasing* ligand field splitting since the difference between the average energy of the e_g_^*^- and t_2g_-like orbitals is decreasing with the chloro substitutions; the difference of the values calculated are 5.039, 4.994, and 4.955 eV for **H0**, **H1**, and **H2**, respectively. The **Sx** series shows the same decreasing trend with the respective e_g_^*^–t_2g_ energy differences being 4.934, 4.875, and 4.819 eV for **S0**, **S1**, and **S2**, respectively. While these numbers are probably overestimated at this level of theory, their qualitative trend reliably indicates the decrease of the ligand field upon substitution along the series. Moreover, the variation of the quintet–singlet energy gaps, which we will discuss later, also supports a decreasing ligand field.

The origin of these apparently anomalous spectral shifts can be explained if we further examine the orbitals involved in the transition. The electron density change at these transitions reveals the obvious involvement of the d_*xy*_ orbital, and one can make out a general axial to side electron density transfer. Also, the Cl substituent seems to have a role by transferring p electrons at the transitions. (See Figures S9 and S10 in the Supporting Information.) Nevertheless, the energy diagram is more elucidating. As it is clear from Figure 6, the energies of the π(*xy*) orbitals are practically unaffected by the Cl substitutions. The π*(*xz*), π*(*yz*) (LUMO, LUMO + 1) orbitals have a strong weight from the π* orbitals of the side rings, which makes them more susceptible for the effects of the substitution; this is manifested in the variations of the energy of these orbitals. These energy differences give the energy of the transition; the corresponding wavelengths are given in the figure. Consequently, the most crucial factor to understand the spectral behavior is the insensitivity of the π(*xy*) orbitals to these substitutions, which results in an almost constant orbital energy.

Finally, no transitions in the UV–vis spectra are linked to the e_g_ and the e_g_^*^ orbitals, yet it is important to address them since they are related to the Fe–N bonding. The e_g_(-like) orbitals are always occupied, and their energies are relatively deep, while the e_g_^*^ orbitals can only become populated in excited states, following a photoexcitation. Their case is somewhat similar to that of the HOMO and LUMO orbitals discussed earlier and very similar for both series. The e_g_^*^–e_g_ energy difference shows an 0.1 eV increase at the mono (5) substitution, but it does not change significantly at the second (5′′) substitution, and the direction of this small change is even opposite to the first one (**H0**, **H1**, and **H2**: 8.53, 8.62, and 8.60 eV; **S0**, **S1**, and **S2**: 8.56, 8.69, and 8.67 eV). Apart from this, the orbital energies shift together due to the variations in the electrostatic effects of the ligands. Importantly, the shifts of these e_g_^*^ and e_g_ orbitals with the (side ring) β Cl substitution are larger than the above changes and also larger than those of the t_2g_-like ones, as we saw in the decreasing ligand field strengths.

After these insights into the ground state electronic structure, we explore the substitution effects on the lowest triplet and quintet states.

### Higher Spin State Molecular Structures and Energies

For the development of low-spin Fe(II)-based molecular switches that have an excited state of sufficient lifetime, the most crucial properties are the relative location of the minimum of the potential energy surface of the lowest quintet state (^5^T_2_ in *O*_*h*_ symmetry) with respect to that of the ground state as well as their crossing, defining the energy barrier between them. ^5^T_2_ is a metal-centered (MC) state with a 3d electron configuration of (t_2g_)^4^(e_g_^*^)^2^. Since both e_g_^*^-type σ-antibonding molecular orbitals are occupied in this state, all of the Fe–N bonds are significantly elongated when compared to the ground state. This quintet can be thermally populated if the ligand field strength is not too big in the so-called spin crossover systems.^57^ In Fe(II) polypyridines, which have a large ligand field, this state can be populated with light excitation. Besides the quintet state, it is also instructive to study the lowest ^3^MC state, ^3^T_1_, which has only one e_g_^*^ orbital populated. In fact, since the symmetry of [Fe(terpy)_2_]^2+^ is lower than that of *O*_*h*_ (*D*_2*d*_), the e_g_^*^ orbitals are split by hundreds of meVs.^19,58^ They will be referred to as  and , where the notation indicates on which Fe 3d orbitals they are based. From these,  is the lower one in energy; thus, this one will be populated in the triplet state ^3^T_1_. Therefore, asymmetrical structural changes are expected when this triplet state is populated, namely, the *r*(Fe–N_eq_) bond lengths are expected to get larger. In Fe(II)–polypyridyl complexes, it is hard to investigate the ^3^T_1_ state, partly because it is difficult to populate, since the direct optical transition is Laporte and spin forbidden, and partly because it is very short lived (τ ≈ 100 fs), as it rapidly decays into the quintet state.^59,60^ Fortunately, quantum chemistry is able to shed light on the structural and energetic properties of this spin state in the complexes investigated.

Based on the above discussion, not only can we expect charge redistributions and the emergence of spin moments in the triplet and quintet states but also the population of the e_g_^*^-like Fe–N antibonding orbitals also causes large structural changes. We present the structural parameters obtained from the optimized geometries in Table 1 as well as the (relative) energies in Table 5 for the three spin states of all of the complexes studied in this paper for completeness and possible comparisons; after a short general analysis, we will only discuss in detail those that belong to the **Hx** and **Sx** series. The calculated structural parameters in Table 1 clearly reflect the general expectations from the population of the e_g_^*^ orbitals. In the triplet state, the *r*(Fe–N_eq_) bond lengths become significantly (ca. 0.15 Å) larger with respect to those of the singlet while the *r*(Fe–N_ax_) bond length barely changes, in agreement with the calculations showing the population of the ; this orbital has its lobes oriented toward the N_eq_ atoms in the equatorial plane. In the quintet state, both types of Fe–N bonds become more than 0.2 Å (ca. 10%) larger than those in the singlet ground state because of the population of both e_g_^*^ antibonding orbitals. In addition, it is also evident from the data that the two *r*(Fe–N_eq_) bond lengths are not identical, but those impacted by the substitution, except for the 3′-Cl, are longer in both the triplet and the quintet states.

**Table 5:** Calculated (B3LYP*/TZVP) Quintet–Singlet (Δ*E*_HL_) and Triplet–Singlet (Δ*E*_T_) Energy Differences, Approximate Barrier Heights (Δ*E*^‡^) for the Two Approaches Described in the Main Text, Given as Energy Differences above the Quintet Minimum, Predicted Relative Lifetime Ratios, Measured Quintet Lifetimes (τ), and Derived Experimental Relative Lifetimes^a^

					relative lifetime				relative lifetime (**S0**)		
notation or substituents of terpy	Δ*E*_HL_ (meV)	Δ*E*_T_ (meV)	Δ*E*_X_^‡^ (meV)	Δ*E*_MECP_^‡^ (meV)	X	MECP	τ (ns)	relative lifetime (exp)	X	MECP	exp
**H0**	505	684	103	118	**1**	**1**	2.72^b^	**1**	**0.4**	0.3	**0.5**
**H1** (5-Cl)	476	667	113	143	**1.5**	2.8	3.64	**1.3**	**0.6**	0.7	**0.6**
**H2** (5,5′′-di-Cl)	450	644	122	144	2.1	2.9	4.19	1.5	0.9	**0.7**	**0.7**
**S0** (4′-SMe)	440	679	126	152	**2.5**	3.9	5.92^b^	**2.2**	**1**	**1**	**1**
**S1** (5-Cl-4′-SMe)	403	637	140	157	4.3	4.9	7.28	2.7	1.7	**1.2**	**1.2**
**S2** (5,5″-di-Cl-4′-SMe)	347	609	161	171	10.2	8.3	8.29	3.1	4.1	2.1	1.4
4′-Cl	482	681	111	115	**1.4**	0.9	4.20	**1.6**	**0.5**	0.2	**0.7**
4-Cl	485	676	110	129	1.3	1.6					
3′-Cl	576	802	81	120	0.4	1.1					
3-Cl	546	729	90	119	0.6	1.1					
6-Cl	141		254	226	408	76.7					
6,6″-di-Cl	–261	70									

a In addition to the **H0** reference, relative lifetimes are also given with respect to **S0** complex on the right of the table. The uncertainty of the lifetimes measured are below 0.05 ns in each case.

b From ref (26).

The Δ*E*_HL_ quintet energies are rather high, in agreement with the strong ligand field, see Table 5. The only notable exceptions to this are the 6-Cl and 6,6″-di-Cl substitutions because of the steric destabilization discussed before. In the case of the [Fe(6,6″-di-Cl-terpy)_2_]^2+^ complex, Δ*E*_HL_ is even a large negative value, suggesting that the ground state of this complex is the quintet—this is exactly what was reported experimentally for this system in ref (52). The Δ*E*_T_ triplet energies are 150–350 meV higher than the quintet energies.

**Figure 7:**
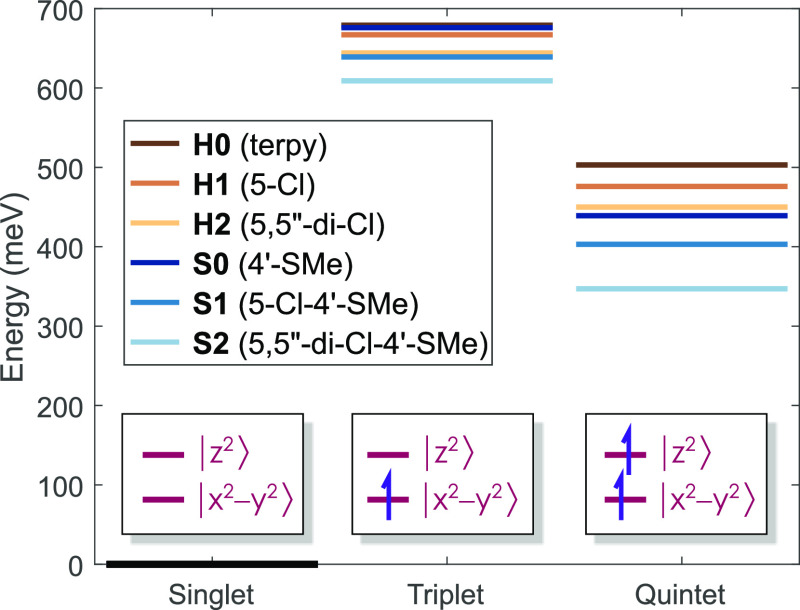
Diagram of the calculated (B3LYP*/TZVP) relative energies for the lowest singlet, triplet, and quintet states for the derivatives of [Fe(terpy)_2_]^2+^ studied. (In the lower part, the occupation of the - and the -based molecular orbitals are depicted for each state.)

Regarding the **Hx** and the **Sx** complexes, in the triplet state there are little structural variations in the FeN_6_ core linked to the substitution with Cl at positions 5 and 5′′: *r*(Fe–N_ax_) and the average of *r*(Fe–N_eq_) varies between 0.001 and 0.004 Å throughout the two series (Table 1). In the case of **H1** and **S1**, the elongation of *r*(Fe–N_eq_) is asymmetrical: the bond length between the Fe and N_eq_(Cl) is about 0.02 Å longer than its nonsubstituted counterpart. This modification of the side rings was expected to change the energy of the triplet state via the now populated  since this orbital is formed by the mixing of the Fe  and the appropriate p orbitals of the N_eq_ atoms; thus, the changes in the charge of the N_eq_ atoms affect mixing and the energy of the resulting orbital. The calculated energy difference of the singlet and triplet minimum (Δ*E*_T_) changes systematically upon Cl substitution at the 5 and 5′′ positions, as seen in Table 5 and Figure 7. For the **Hx** series, Δ*E*_T_ decreases by 17 and 40 meV after the mono- and dichloro substitutions, respectively. The substitution at the axial 4′ position has a negligible effect on Δ*E*_T_, as apparent from the comparison of the energies of **H0** and **S0**; this is not surprising as we already saw from the discussion of the ground state properties in Figure 3 that the 4′ substitution affects the charge on N_ax_ but leaves the charge of N_eq_ unaltered. Nevertheless, it apparently reinforces the effect of the substituent placed on the side rings: the mono- and disubstitution reduces Δ*E*_T_ of [Fe(4′-SMe-terpy)_2_]^2+^ derivatives by 40 and 70 meV, see Figure 7.

In the case of the quintet state, the structural variations that take place due to the 5-Cl and 5,5″-di-Cl substitutions are rather tiny: a ca. 0.005 Å shortening for both *r*(Fe–N_ax_) and *r*(Fe–N_eq_) is observed, accompanied by a 0.3° difference in the NNN angle at most, as is apparent from the data shown in Table 1. Yet, the decrease of Δ*E*_HL_ is more prominent than that in the triplet, 30–40 meV for the mono-substituted and 75–90 meV for the dichloro-substituted ligands (Table 5). Since there is a relevant energy shift between the 2 series (because of the 4′-SMe substituents), together they span a rather large 150 meV range (see Figure 7), which is promising as the Δ*E*_HL_ values are expected to influence the quintet lifetime.

### Quintet–Singlet Energy Barriers and Predicted and Measured Quintet Lifetimes

For the light-excited Fe(II) coordination compounds, Hauser (on the basis of earlier works^61^) described that the quintet state relaxes via a nonadiabatic multiphonon process and showed that at low temperatures a temperature-independent quantum tunneling process determines the relaxation rate, which is inversely proportional to Δ*E*_HL_; this was called the inverse energy gap law.^62^ At higher temperatures (ca. *T* > 50 K), the process becomes thermally activated and follows the Arrhenius law. Here, the relaxation rate depends on the classical barrier between the quintet and the singlet states. Assuming parabolic potentials which are only vertically shifted (i.e., different Δ*E*_HL_ but same bond length changes), it is easy to see that the barrier height still depends inversely on the energy gap. Therefore, it would be advantageous to retain this concept to estimate barrier heights and HS lifetimes.

In a previous work, we could successfully estimate the quintet lifetimes through a series of 4′-substituted [Fe(terpy)_2_]^2+^ derivatives, capitalizing on the similarity of those molecules.^26^ The barrier height Δ*E*_X_^‡^ was obtained by shifting high-accuracy CASPT2 level potential energy surfaces of the parent compound [Fe(terpy)_2_]^2+^, obtained earlier,^19^ to the calculated Δ*E*_HL_, and a simple numerical routine was used to determine the energy at the crossing point. Given the reasonable similarity of the complexes also in the current study and the systematic trend in the Δ*E*_HL_ values with the substitutions, we decided to also test the applicability of this shifted PES-crossing (“X”) approximation here, although the assumption that the substitution alters only the energy difference of the potential energy surfaces while both the distance of the quintet and the singlet minimum and the steepness of the PES remain unaltered is certainly weaker in this case, where the substitution patterns are more diverse than in the previous study. As it is apparent from the data listed in Table 5, Δ*E*_X_^‡^ ranges from 103 to 161 meV (above the quintet minimum) and also increases stepwise with the 5 and 5′′ Cl substitutions.

The barrier height can also be estimated by locating the minimum energy crossing point (MECP)^43^ of the two relevant potential energy surfaces, which is carried out by performing a geometry optimization while minimizing the energy difference of the states involved, taking all nuclear degrees of freedom into account. We have calculated the MECP between the quintet and the singlet states  for the complexes in this paper and found that they range from 119 to 170 meV (above the quintet minimum). They increase as a result of substitution with Cl at positions 5 and 5″, so we expect that the respective quintet lifetimes increase as well.

The range of Δ*E*_X_^‡^ is similar to that of Δ*E*_MECP_^‡^, but in this case, we got increasing results with more evenly distributed values; in the case of the MECP, there is also a gap between **H0** and the rest of the series.

We estimated lifetimes relative to that of [Fe(terpy)_2_]^2+^ as the selected reference using the Δ*E*_MECP_^‡^ and Δ*E*_X_^‡^ barrier values obtained as discussed above employing the Arrhenius equation, with the assumption that the pre-exponential factors do not significantly differ from compound to compound: .

**Figure 8:**
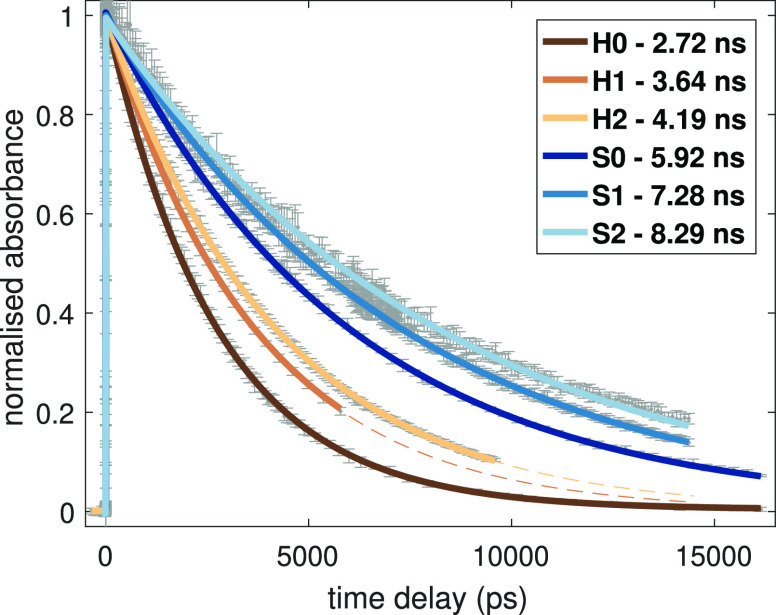
Evolution of the normalized GSB signal for the derivatives of [Fe(terpy)_2_]^2+^ complex with substituents as indicated in the legend and their fits with a step function and an exponential decay to determine the quintet lifetime.

The relative lifetimes determined this way can be compared with those obtained from transient optical absorption spectroscopy (TOAS) measurements. TOAS has been measured for 5 complexes with Cl-substituted ligands with **H1**, **H2**, **S1**, **S2**, and 4′-Cl; the TOAS of the complexes with unsubstituted terpy and the 4′-SMe-terpy ligands we have reported previously.^26^ As for most Fe(II) polypyridyl complexes, after the excitation the system arrives into the long-lived quintet excited state within 1 ps. The transient spectra are dominated by the ground state bleach (GSB); for the quintet excited state, absorption is negligible in the wavelength range of our optical probe (see Figure S11 in the Supporting Information). Therefore, the time evolution of the population of the quintet can be followed by the disappearance of the GSB. Accordingly, the TOAS data were fitted with a single-exponential decay, as displayed in Figure 8. For [Fe(terpy)_2_]^2+^ and its Cl-bearing derivatives **H1** and **H2**, we found that mono- and disubstitutions increased the lifetime τ from 2.72 to 3.64 ns and 4.19 ns, and in the case of the 4′-SMe-based series, τ increased from the original 5.92 to 7.28 ns and 8.29 ns for **S1** and **S2**, respectively, see Table 5.

The estimates based on Δ*E*_MECP_^‡^ and Δ*E*_X_^‡^ both predicted that these Cl substitutions will increase the lifetime of the quintet state. This is consistent with the TOAS measurements. Based on the numbers presented in Table 5, we can conclude that when compared to the experimental results, the relative lifetime calculated from Δ*E*_X_^‡^ shows a reasonable agreement for 4 out of the 7 measured compounds: **H1**, **H2**, 4′-Cl, and **S0**; however, they significantly overestimate the quintet lifetime for the 4′-SMe derivatives **S1** and **S2**.

Curiously, the lifetimes obtained from Δ*E*_MECP_^‡^ do not correspond to the experimental results very well. They are at least 75% or more off and only provide a better approximation to the experiment for the **S2** case, yet even this one is about 167% off from the observed lifetime. So, we have to conclude that the intuitive but simple approximation seems to work reasonably, while the one which is in principle better justified gives poorer results. The reasons for this remain unclear, and it will require further studies to better understand which factors lead to this apparent anomaly in which the simplicity of the approximations and the accuracy limits of DFT must have a considerable role. However, changing the reference to **S0** gives better agreement with the calculated relative lifetimes, except for that of **S2**, which remains largely overestimated. This choice is more arbitrary, and the better result can stem from a mere favorable coincidence; yet, we decided to present it as this also suggests that the potential of this approach can be larger than expected from the results with the **H0** reference. Nevertheless, even if the predictions are not fully satisfying quantitatively, they are qualitatively sufficient enough to encourage further applications in ligand design and molecular engineering. Substitution with groups of more pronounced EW/ED effects can also lead to larger changes in the photophysical properties; a detailed theoretical and experimental investigation to explore this is ongoing.

## Conclusions

We demonstrated how the physical and photophysical properties of the [Fe(terpy)_2_]^2+^ complex could be tuned by substituting individual hydrogens with a Cl atom at different positions of the terpyridine ligand. We carried out DFT calculations in order to find the position with the largest impact on the electronic structure. Based on the computed atomic charges within the FeN_6_ core, we inferred that after the previously studied position 4′ substitution, substitution at position 5 is the second most effective in this respect. Therefore, we performed further calculations for this type of substitution of [Fe(terpy)_2_]^2+^, extending it to both side rings, i.e., the double 5,5″-dichloro substitution. The same procedure was repeated for [Fe(4′-SMe-terpy)_2_]^2+^, which has an ED group on the molecular axis, allowing us to form a first assessment on the additivity of the effects from different groups. Moreover, we also prepared and experimentally characterized the complexes. The molecular structure from crystallography, where available, agrees with the structure obtained from DFT calculations. Changes in the atomic charges within the FeN_6_ core are supported by Mössbauer spectra. The UV–vis spectra show systematic shifts in the UV range that can be satisfactorily accounted for by TD-DFT through orbital analysis. The latter contradicts earlier claims that such shifts reflect the ligand field splitting. The lowest triplet and quintet state energies and geometries were also determined. The quintet–singlet energy barriers were estimated using two approaches: one based on the inverse energy gap law of spin-crossover compounds and the other on directly calculating the minimum energy crossing points between the potential energy surfaces of the quintet and singlet states. From these barriers, the quintet lifetimes were predicted and compared to those determined with TOAS. Overall, although achieving the required accuracy by DFT is a challenge for these molecules, the auspicious qualitative results should encourage one to further exploit the available theoretical toolkit for modifying the potential energy landscapes of such promising light-switchable molecules in order to improve their functionality.

## Supporting Information Available

Optimized molecular geometries and energies, schemes on the synthesis of the ligands, NMR and Mössbauer spectra, and cyclic voltammetry for the complexes (PDF)
