# Supplementary material for: Molecular Engineering to Tune Functionality: The Case of Cl-Substituted [Fe(terpy)2]2+
Source: Inorg Chem. 2023 Apr 11;62(16):6397–410. doi: 10.1021/acs.inorgchem.3c00271 (PMC10131222; doi:10.1021/acs.inorgchem.3c00271)
Supplement: Supplementary file 1 — ic3c00271_si_001.pdf [file ic3c00271_si_001.pdf]

Supporting Information for the Article

"Molecular engineering to tune functionality: the case of Cl substituted $[Fe(terpy)_2]^{2+}$"

Mariann Papp,[†,‡] Tamás Keszthelyi,[†] Andor Vancza,[†,¶] Éva G. Bajnóczi,[†] Éva Kováts,[†] Zoltán Németh,[†] Csilla Bogdán,[†] Gábor Bazsó,[†] Tamás Rozgonyi,[†] and György Vankó[*,†]

†*Wigner Research Centre for Physics, P.O. Box 49, H-1525 Budapest, Hungary*
‡*Hevesy György PhD School of Chemistry, Eötvös Loránd University, Pázmány Péter sétány 1/A, H-1117 Budapest, Hungary*
¶*Department of Physical Chemistry and Materials Science, Faculty of Chemical Technology and Biotechnology, Budapest University of Technology and Economics, Műegyetem rkp. 3., H-1111 Budapest, Hungary*

E-mail: vanko.gyorgy@wigner.hu

Contents

1 Energies **3**
1.1 B3LYP*/TZVP energies in Hartree . . . 3
1.2 BP86/TZVP energies in Hartree 3

2 Molecular structures **3**
2.1 Selected structural parameters obtained with B3LYP*/TZVP 3
2.2 MECP structural parameters 4
2.3 XRD 4

3 Spectra **4**
3.1 Mössbauer spectra 4
3.2 Cyclic voltammetry 5
3.3 NMR 5

4 Reaction schemes for ligand synthesis **7**

5 Electron density differences for transitions in the 300-330 nm region of the UV–vis spectra **9**

6 TOAS **10**

7 Optimized BP86/TZVP level geometries of the studied complexes **11**
7.1 Singlet state 11
7.1.1 $[Fe(terpy)_2]^{2+}$(**H0**) 11
7.1.2 $[Fe(3\text{-Cl-terpy})_2]^{2+}$ 11
7.1.3 $[Fe(4\text{-Cl-terpy})_2]^{2+}$ 11
7.1.4 $[Fe(5\text{-Cl-terpy})_2]^{2+}$ (**H1**) 12
7.1.5 $[Fe(5,5''\text{-diCl-terpy})_2]^{2+}$ (**H2**) . . 12
7.1.6 $[Fe(6\text{-Cl-terpy})_2]^{2+}$ 13
7.1.7 $[Fe(4'\text{-Cl-terpy})_2]^{2+}$ 13
7.1.8 $[Fe(3'\text{-Cl-terpy})_2]^{2+}$ 14
7.1.9 $[Fe(4'\text{-SMe-terpy})_2]^{2+}$(**S0**) 14
7.1.10 $[Fe(5\text{-Cl-}4'\text{-SMe-terpy})_2]^{2+}$ (**S1**) . 14
7.1.11 $[Fe(5,5''\text{-diCl-}4'\text{-SMe-terpy})_2]^{2+}$ (**S2**) 15
7.2 Triplet state 15
7.2.1 $[Fe(terpy)_2]^{2+}$(**H0**) 15
7.2.2 $[Fe(3\text{-Cl-terpy})_2]^{2+}$ 16
7.2.3 $[Fe(4\text{-Cl-terpy})_2]^{2+}$ 16
7.2.4 $[Fe(5\text{-Cl-terpy})_2]^{2+}$ (**H1**) 17
7.2.5 $[Fe(5,5\text{-diCl-terpy})_2]^{2+}$ (**H2**) . . . 17
7.2.6 $[Fe(6\text{-Cl-terpy})_2]^{2+}$ 18
7.2.7 $[Fe(4'\text{-Cl-terpy})_2]^{2+}$ 18
7.2.8 $[Fe(3'\text{-Cl-terpy})_2]^{2+}$ 19
7.2.9 $[Fe(4'\text{-SMe-terpy})_2]^{2+}$(**S0**) 19
7.2.10 $[Fe(5\text{-Cl-}4'\text{-SMe-terpy})_2]^{2+}$ (**S1**) . 20
7.2.11 $[Fe(5,5''\text{-diCl-}4'\text{-SMe-terpy})_2]^{2+}$ (**S2**) 21
7.3 Quintet 21
7.3.1 $[Fe(terpy)_2]^{2+}$(**H0**) 21
7.3.2 $[Fe(3\text{-Cl-terpy})_2]^{2+}$ 21
7.3.3 $[Fe(4\text{-Cl-terpy})_2]^{2+}$ 22
7.3.4 $[Fe(5\text{-Cl-terpy})_2]^{2+}$ (**H1**) 22
7.3.5 $[Fe(5,5''\text{-diCl-terpy})_2]^{2+}$ (**H2**) . . 23
7.3.6 $[Fe(6\text{-Cl-terpy})_2]^{2+}$ 23
7.3.7 $[Fe(4'\text{-Cl-terpy})_2]^{2+}$ 24
7.3.8 $[Fe(3'\text{-Cl-terpy})_2]^{2+}$ 24
7.3.9 $[Fe(4'\text{-SMe-terpy})_2]^{2+}$(**S0**) 25
7.3.10 $[Fe(5\text{-Cl-}4'\text{-SMe-terpy})_2]^{2+}$ (**S1**) . 25
7.3.11 $[Fe(5,5''\text{-diCl-}4'\text{-SMe-terpy})_2]^{2+}$ (**S2**) 25

8 Optimized B3LYP*/ TZVP level geometries of the studied complexes **27**
8.1 Singlet state 27
8.1.1 $[Fe(terpy)_2]^{2+}$(**H0**) 27

8.1.2 [Fe(3-Cl-terpy)$_2$]$^{2+}$ 27
8.1.3 [Fe(4-Cl-terpy)$_2$]$^{2+}$ 27
8.1.4 [Fe(5-Cl-terpy)$_2$]$^{2+}$ (**H1**) 28
8.1.5 [Fe(5,5"-diCl-terpy)$_2$]$^{2+}$ (**H2**) . . 28
8.1.6 [Fe(6-Cl-terpy)$_2$]$^{2+}$ 29
8.1.7 [Fe(4'-Cl-terpy)$_2$]$^{2+}$ 29
8.1.8 [Fe(3'-Cl-terpy)$_2$]$^{2+}$ 30
8.1.9 [Fe(4'-SMe-terpy)$_2$]$^{2+}$(**S0**) 30
8.1.10 [Fe(5-Cl-4'-SMe-terpy)$_2$]$^{2+}$ (**S1**) . 30
8.1.11 [Fe(5,5"-diCl-4'-SMe-terpy)$_2$]$^{2+}$ (**S2**) 31
8.2 Triplet state 31
8.2.1 [Fe(terpy)$_2$]$^{2+}$(**H0**) 31
8.2.2 [Fe(3-Cl-terpy)$_2$]$^{2+}$ 32
8.2.3 [Fe(4-Cl-terpy)$_2$]$^{2+}$ 32
8.2.4 [Fe(5-Cl-terpy)$_2$]$^{2+}$ (**H1**) 33
8.2.5 [Fe(5,5"-diCl-terpy)$_2$]$^{2+}$ (**H2**) . . 33
8.2.6 [Fe(6-Cl-terpy)$_2$]$^{2+}$ 34
8.2.7 [Fe(4'-Cl-terpy)$_2$]$^{2+}$ 34
8.2.8 [Fe(3'-Cl-terpy)$_2$]$^{2+}$ 35
8.2.9 [Fe(4'-SMe-terpy)$_2$]$^{2+}$(**S0**) 35
8.2.10 [Fe(5-Cl-4'-SMe-terpy)$_2$]$^{2+}$ (**S1**) . 36
8.2.11 [Fe(5,5"-diCl-4'-SMe-terpy)$_2$]$^{2+}$ (**S2**) 37
8.3 Quintet state 37
8.3.1 [Fe(terpy)$_2$]$^{2+}$(**H0**) 37
8.3.2 [Fe(3-Cl-terpy)$_2$]$^{2+}$ 37
8.3.3 [Fe(4-Cl-terpy)$_2$]$^{2+}$ 38
8.3.4 [Fe(5-Cl-terpy)$_2$]$^{2+}$ (**H1**) 38
8.3.5 [Fe(5,5"-diCl-terpy)$_2$]$^{2+}$ (**H2**) . . 39
8.3.6 [Fe(6-Cl-terpy)$_2$]$^{2+}$ 39
8.3.7 [Fe(4'-Cl-terpy)$_2$]$^{2+}$ 40
8.3.8 [Fe(3'-Cl-terpy)$_2$]$^{2+}$ 40
8.3.9 [Fe(4'-SMe-terpy)$_2$]$^{2+}$(**S0**) 41
8.3.10 [Fe(5-Cl-4'-SMe-terpy)$_2$]$^{2+}$ (**S1**) . 41
8.3.11 [Fe(5,5"-diCl-4'-SMe-terpy)$_2$]$^{2+}$ (**S2**) 41
8.4 Quintet-singlet MECP 42
8.4.1 [Fe(terpy)$_2$]$^{2+}$(**H0**) 42
8.4.2 [Fe(3-Cl-terpy)$_2$]$^{2+}$ 42
8.4.3 [Fe(4-Cl-terpy)$_2$]$^{2+}$ 42
8.4.4 [Fe(5-Cl-terpy)$_2$]$^{2+}$ (**H1**) 43
8.4.5 [Fe(5,5"-diCl-terpy)$_2$]$^{2+}$ (**H2**) . . 43
8.4.6 [Fe(6-Cl-terpy)$_2$]$^{2+}$ 44
8.4.7 [Fe(4'-Cl-terpy)$_2$]$^{2+}$ 44
8.4.8 [Fe(3'-Cl-terpy)$_2$]$^{2+}$ 45
8.4.9 [Fe(4'-SMe-terpy)$_2$]$^{2+}$(**S0**) 45
8.4.10 [Fe(5-Cl-4'-SMe-terpy)$_2$]$^{2+}$ (**S1**) . 45
8.4.11 [Fe(5,5"-diCl-4'-SMe-terpy)$_2$]$^{2+}$ (**S2**) 46

References **47**

1 Energies

1.1 B3LYP*/TZVP energies in Hartree

Table S1: (U)KS B3LYP*/TZVP energies in Hartree for the minima of the lowest singlet, triplet and quintet sates, and the singlet-quintet MECP, respectively

substituents	LS	IS	HS	MECP
-	-2746.94898232494	-2746.92385605015	-2746.93042015235	-2746.92609807386
4'-Cl	-3665.84725175770	-3665.82222051484	-3665.82952551607	-3665.82530377698
5-Cl (**H1**)	-3665.84629913222	-3665.82179273529	-3665.82881979172	-3665.82355951788
5,5"-di-Cl (**H2**)	-4584.74291638999	-4584.71925281364	-4584.72636965715	-4584.72107486366
4'-SMe (**S0**)	-3621.59476664765	-3621.56982411297	-3621.57861666646	-3621.57303331804
5-Cl-4'-SMe (**S1**)	-4540.49191819359	-4540.46851752596	-4540.47711157238	-4540.47133900991
5,5"-di-Cl-4'-SMe (**S2**)	-5459.38801918280	-5459.36562628020	-5459.37527036239	-5459.36900576030
3'-Cl	-3665.83165184150	-3665.80218778732	-3665.81047588546	-3665.80607450277
4-Cl	-3665.84935719232	-3665.82452074038	-3665.83153554350	-3665.82679020727
3-Cl	-3665.83055295870	-3665.80377117939	-3665.81050173317	-3665.80613073830
6-Cl	-3665.83355977559	-3665.81879715020	-3665.82839551720	-3665.82008549010

1.2 BP86/TZVP energies in Hartree

Table S2: (U)KS BP86/TZVP energies in Hartree for the minima of the lowest singlet, triplet and quintet sates.

substituents	LS	IS	HS
-	-2749.28642377464	-2749.243945158539	-3668.52959371741
4'-Cl	-3668.58512885317	-3668.52691877395	-3668.51413967421
5-Cl (**H1**)	-3668.58399890601	-3668.54247502973	-3668.53013601757
5,5"-di-Cl (**H2**)	-4587.88148657276	-4587.84080820041	-4587.82848236142
4'-SMe (**S0**)	-3624.42148965820	-3624.37927440536	-3624.36982720746
5-Cl-4'-SMe (**S1**)	-4543.71963245894	-4543.67783766208	-4543.66943647313
5,5"-di-Cl-4'-SMe (**S2**)	-5463.01735760780	-5462.97679096799	-5462.96850164218
3'-Cl	-3668.57164202327	-3668.52550724802	-3668.51447628428
4-Cl	-3668.58719805591	-3668.54549580895	-3668.53318649400
3-Cl	-3668.57068229988	-3668.52691877395	-3668.51413967421
6-Cl	-3668.56824238224	-3668.53831505341	-3668.52959371741

2 Molecular structures

2.1 Selected structural parameters obtained with B3LYP*/TZVP

Table S3: Selected structural parameters of the FeN_6 core in the B3LYP*/TZVP optimized molecular geometries for the singlet and quintet states of the studied derivatives of the $[Fe(terpy)_2]^{2+}$complex

substituents	Singlet			Triplet			Quintet		
of terpy	r(Fe-N_{ax})	r(Fe-N_{eq})	NNN	r(Fe-N_{ax})	r(Fe-N_{eq})	NNN	r(Fe-N_{ax})	r(Fe-N_{eq})	NNN
-	1.909 Å	2.019 Å	103.23°	1.936 Å	2.161 Å	108.72°	2.152 Å	2.227 Å	108.57°
5-Cl (**H1**)	1.912 Å	2.019 Å	103.24°	1.938 Å	2.161 Å	108.73°	2.147 Å	2.229 Å	108.68°
5,5"-di-Cl	1.914 Å	2.021 Å	103.33°	1.940 Å	2.164 Å	108.80°	2.148 Å	2.231 Å	108.84°
4'-SMe (**S0**)	1.908 Å	2.020 Å	103.50°	1.935 Å	2.164 Å	109.11°	2.147 Å	2.233 Å	109.19°
5-Cl-4'-SMe (**S1**)	1.910 Å	2.021 Å	103.56°	1.937 Å	2.165 Å	109.13°	2.165 Å	2.235 Å	109.32°
5,5"-di-Cl-4'-SMe (**S2**)	1.913 Å	2.024 Å	103.64°	1.940 Å	2.168 Å	109.21°	2.138 Å	2.236 Å	109.49°
4'-Cl	1.907 Å	2.017 Å	103.20°	1.934 Å	2.160 Å	108.82°	2.145 Å	2.224 Å	108.68°
4-Cl	1.910 Å	2.017 Å	103.16°	1.936 Å	2.159 Å	108.64°	2.151 Å	2.225 Å	108.49°
3'-Cl (=5'-Cl)	1.914 Å	1.997 Å	102.12°	1.940 Å	2.121 Å	107.28°	2.143 Å	2.197 Å	107.13°
		2.011 Å			2.156 Å			2.204 Å	
3-Cl	1.906 Å	2.011 Å	102.68°	1.935 Å	2.140 Å	108.04°	2.150 Å	2.202 Å	107.90°
		2.004 Å			2.149 Å			2.215 Å	
6-Cl	1.923 Å	2.018 Å	105.06°	1.947 Å	2.126 Å	109.8°	2.157 Å	2.244 Å	109.80°
		2.126 Å			2.274 Å			2.280 Å	
6,6"-diCl	1.932 Å	2.148 Å	107.68°	1.951 Å	2.257 Å	111.99°	2.114 Å	2.327 Å	112.70°

2.2 MECP structural parameters

Table S4: Selected structural parameters of the FeN_6 core in the B3LYP*/TZVP optimized molecular geometries of the singlet-quintet minimum energy crossing points of the derivatives studied of the $[Fe(terpy)_2]^{2+}$complex

substituents of terpy	r(Fe-N$_{ax}$) (Å)	r(Fe-N$_{eq}$) (Å)	NNN (°)
-	2.048	2.168	107.41
5-Cl (**H1**)	2.047	2.166	107.35
5,5"-di-Cl	2.045	2.169	107.51
4'-SMe (**S0**)	2.038	2.169	107.84
5-Cl-4'-SMe (**S1**)	2.040	2.162	107.58
5,5"-di-Cl-4'-SMe (**S2**)	2.039	2.160	107.59
4'-Cl	2.043	2.168	107.57
3'-Cl	2.062	2.150	106.08
3-Cl	2.050	2.158	106.93
4-Cl	2.046	2.166	107.36
6,6"-diCl	1.980	2.210	109.63

2.3 XRD

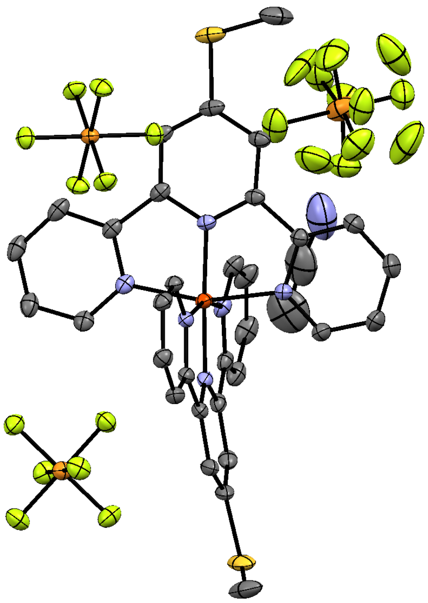

Figure S1: ORTEP diagram of the $(PF_6)^-$ salt of **S0**, taken at 250 K, without the hydrogen atoms, with acetonitrile as crystalline solvent in the background.

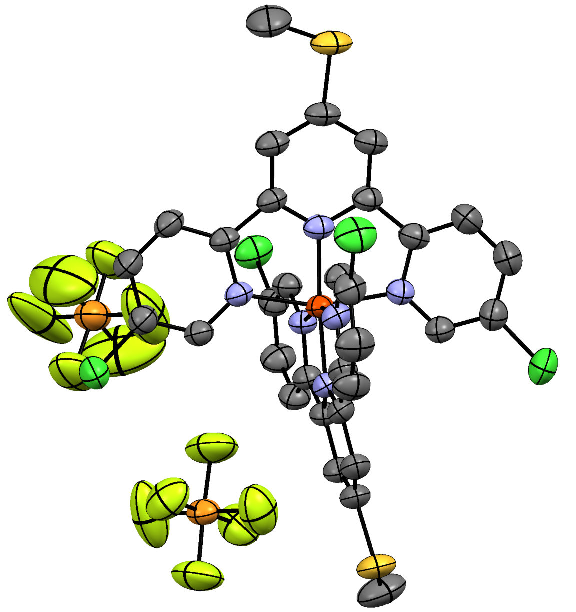

Figure S2: ORTEP diagram of the $(PF_6)^-$ salt of **S2**, taken at 250 K, without the hydrogen atoms.

3 Spectra

3.1 Mössbauer spectra

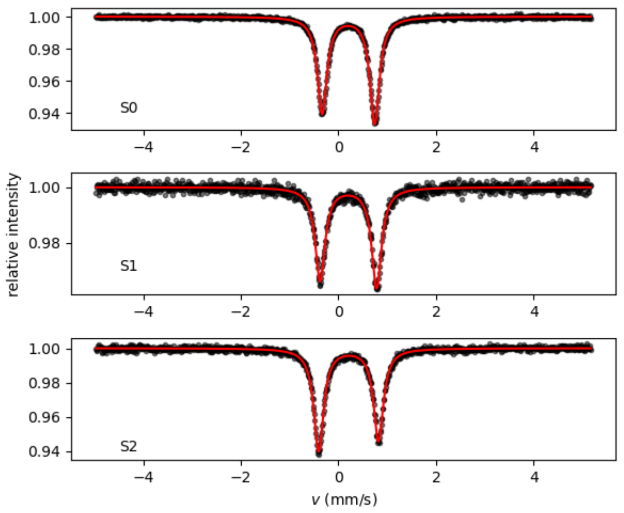

Figure S3: Room temperature ^{57}Fe Mössbauer spectra of the $[Fe(terpy)_2]^{2+}$ complexes with substituents of 4'-SMe (**S0**, *top*), 5-Cl-4'-SMe (**S1**, *middle*) and 5,5"-di-Cl-4'-SMe (**S2**, *bottom*).

3.2 Cyclic voltammetry

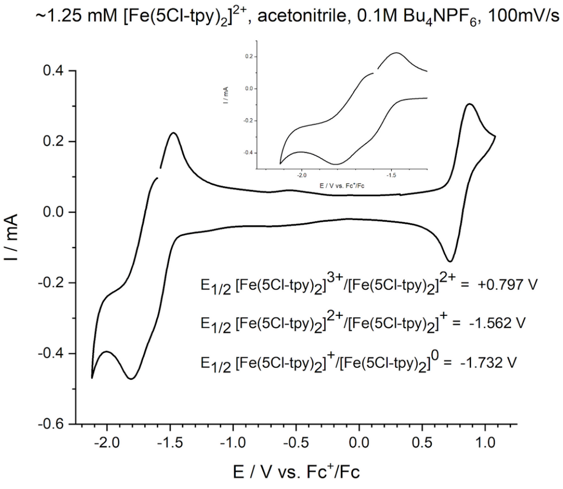

Figure S4: Cyclic voltammetry of **H1**

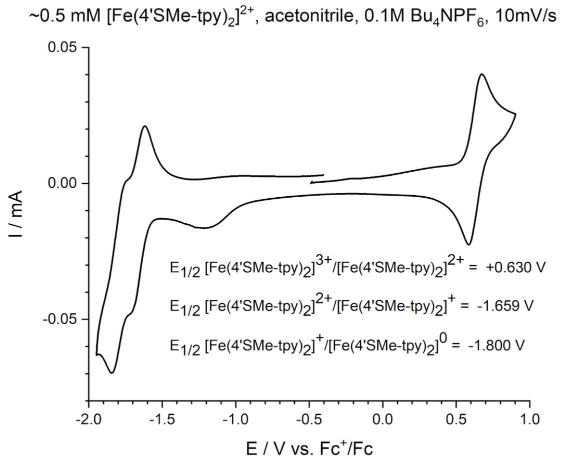

Figure S5: Cyclic voltammetry of **S0**

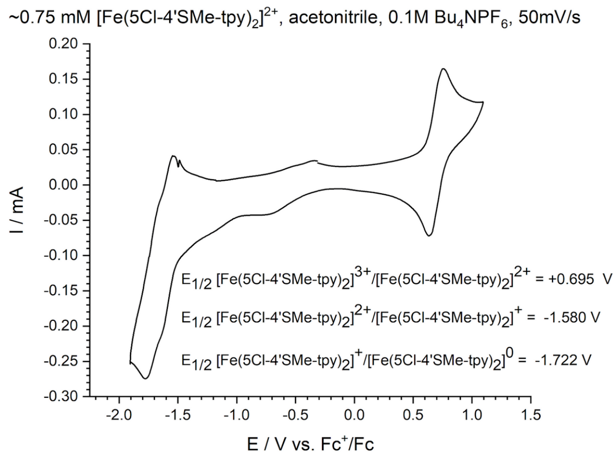

Figure S6: Cyclic voltammetry of **S1**

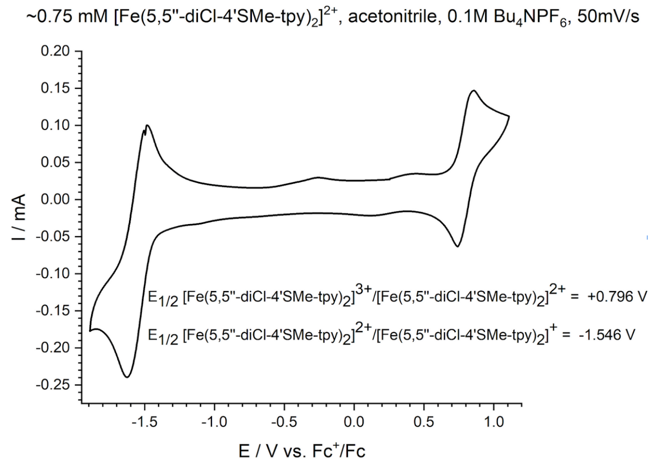

Figure S7: Cyclic volammetry of **S2**

3.3 NMR

The ^{1}NMR of $[Fe(terpy)_2]^{2+}$agreed with the literature spectra by Elsbernd and Beattie.[1] The replacement of the 4'-H by SMe results in the removal of the splitting of the signal from the neighbouring 3' and 5' protons. These lines, which are found at the largest chemical shifts in the spectra with values of δ(3',5')= 8.82ppm and 8.39 ppm, for R = H **H0** and SMe (**S0**), respectively. In Fig. S8 the spectral variations within of the **Sx** series, (4'-SMe, 5-Cl-4'-SMe and 5,5"-di-Cl-4'-SMe) can be followed. With the addition of Cls, the splittig structure for the H in the 4 and 6 positions are getting simpler (since the coupling with H at position 5 (and 5") is removed), and the lines 4,4" are shifted to a little higher, while the lines 6,6" are shifted to a little smaller chemical shifts.

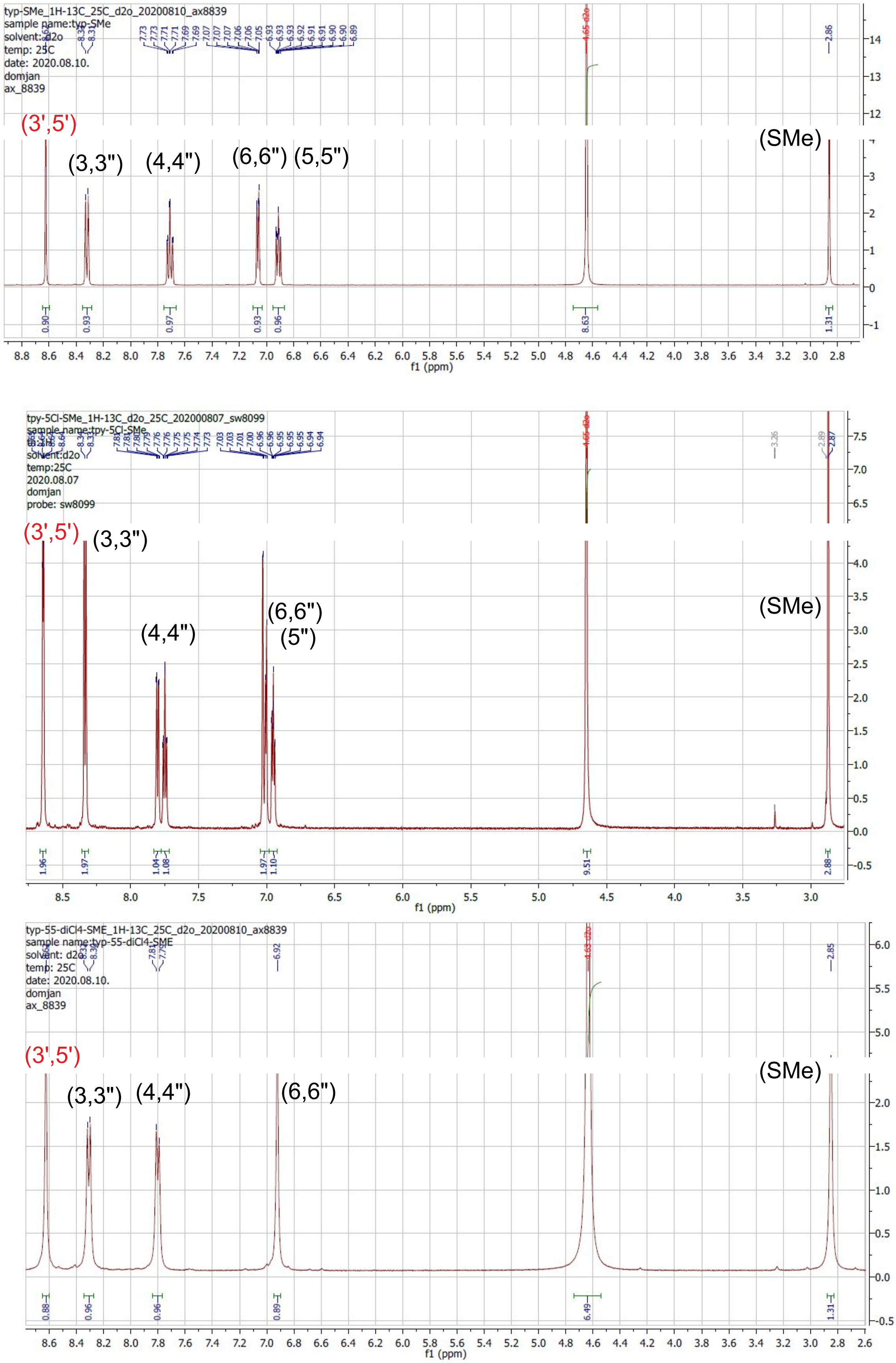

Figure S8: ^{1}H NMR spectra of the $[Fe(terpy)_2]^{2+}$ complexes with substituents of 4'-SMe (**S0**, *top*), 5-Cl-4'-SMe (**S1**, *middle*) and 5,5"-di-Cl-4'-SMe (**S2**, *bottom*).

4 Reaction schemes for ligand synthesis

Synthesis of the 5-Cl-terpyridine derivatives

László Varga, PhD

EvoBlocks Ltd.

laszlo.varga@evoblocks.org

Berlini utca 47-49, H-1045 Budapest, Hungary

1. Reaction schemes

5-Cl-2,2':6',2''-terpyridine

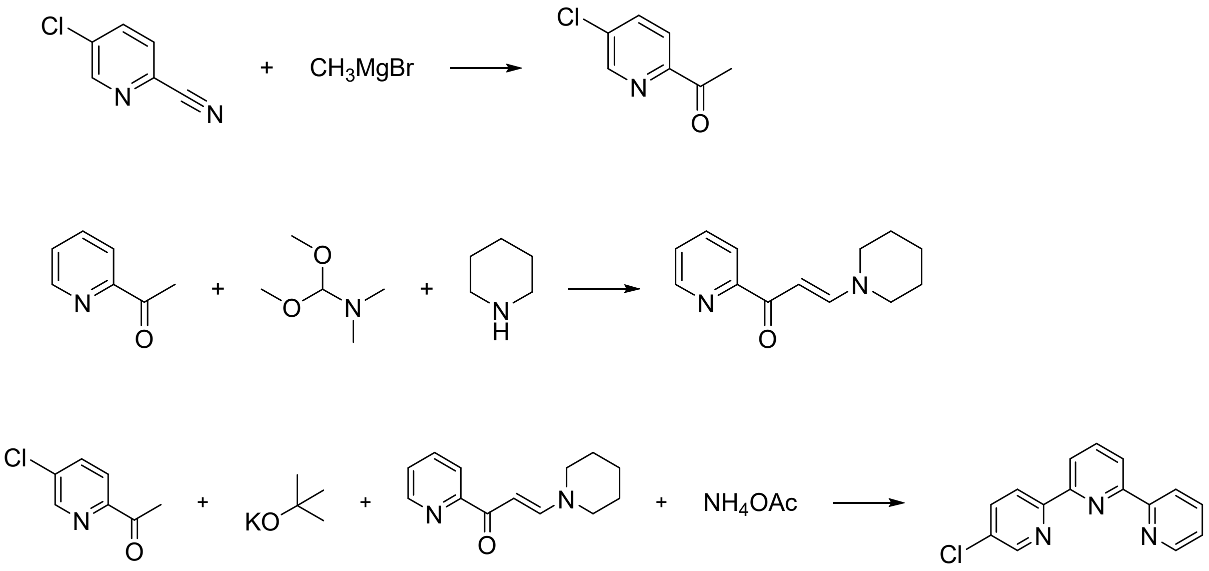

5-chloro-4'-SMe-2,2':6',2''-terpyridine

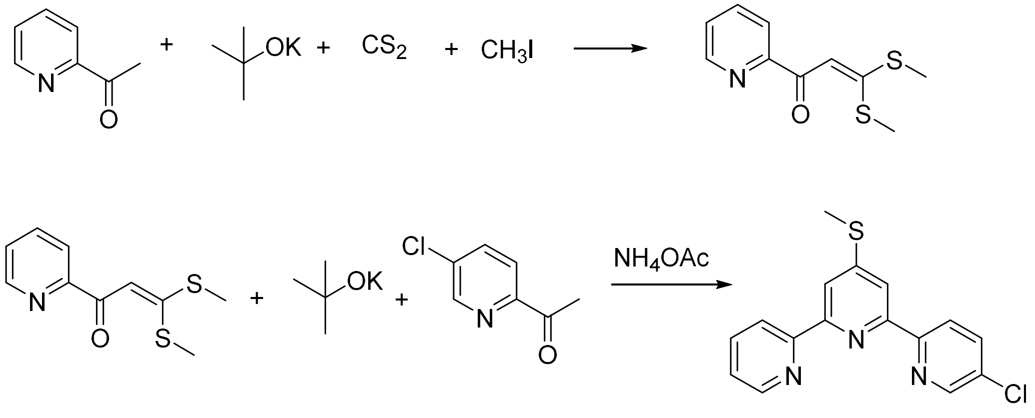

5,5”-dichloro-4'-SMe-2,2':6',2"-terpyridine

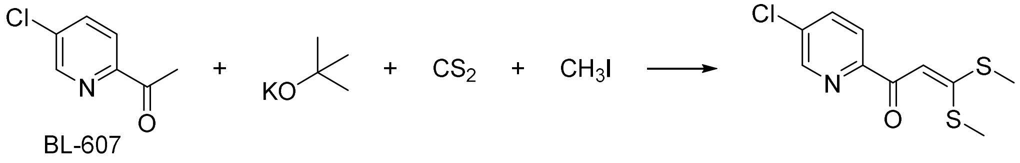

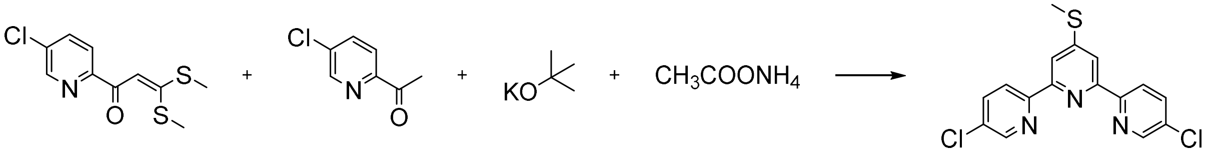

5,5”-diamino-2,2':6',2"-terpyridine

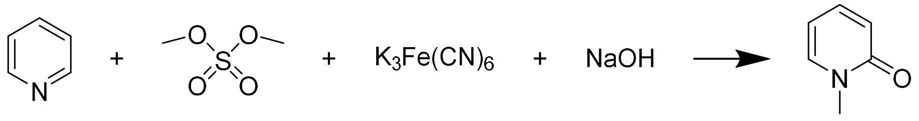

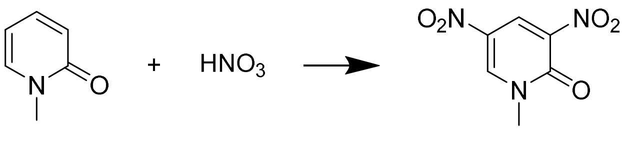

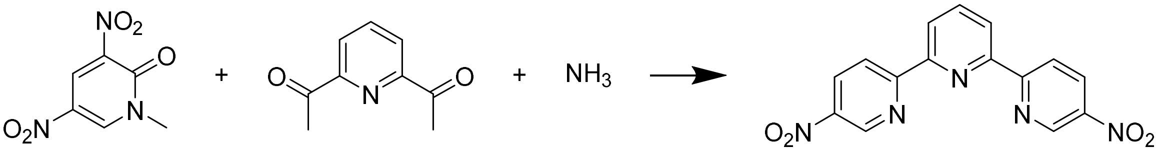

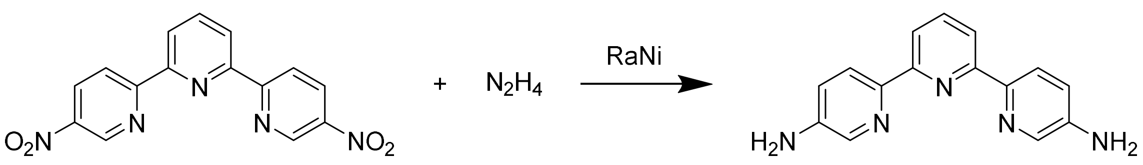

5,5"-dichloro-2,2':6',2"-terpyridine

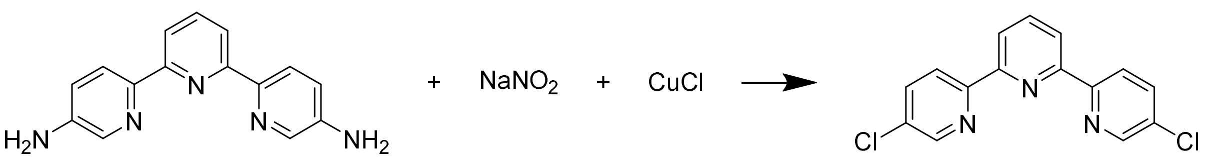

5 Electron density differences for transitions in the 300-330 nm region of the UV–vis spectra

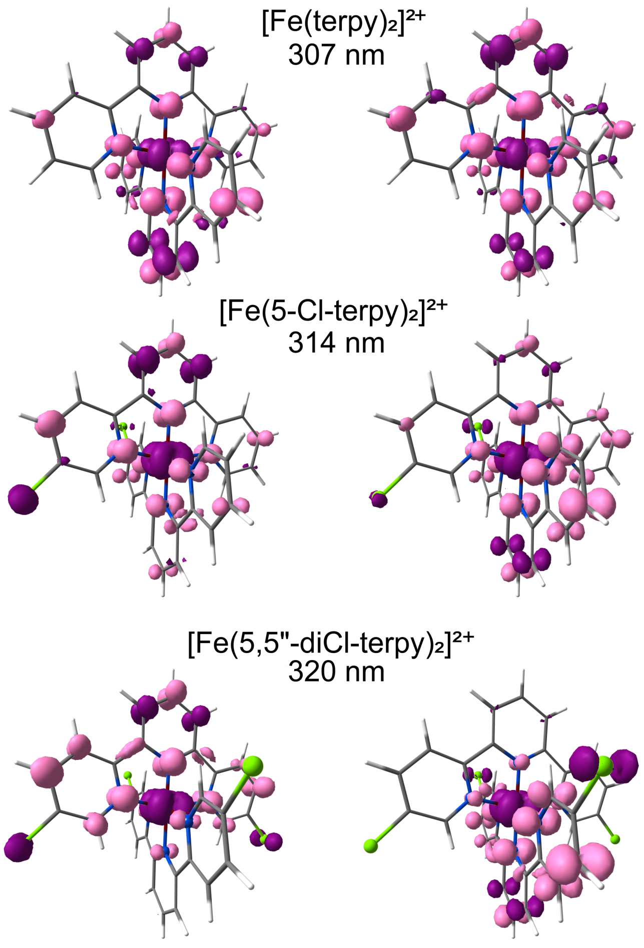

Figure S9: Electron density differences for the transitions in the 300-330 nm region for $[Fe(terpy)_2]^{2+}$and its chloro-substituted derivatives. Pink(purple) indicates an increase(decrease) in the electron density upon excitation.

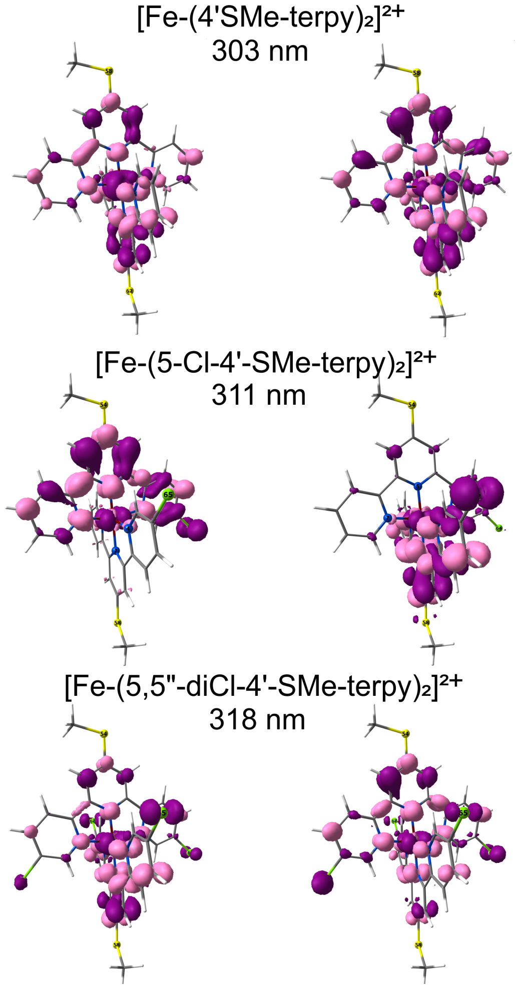

Figure S10: Electron density differences for the transitions in the 300-330 nm region for $[Fe(4'\text{-SMe-terpy})_2]^{2+}$ and its chloro-substituted derivatives.

6 TOAS

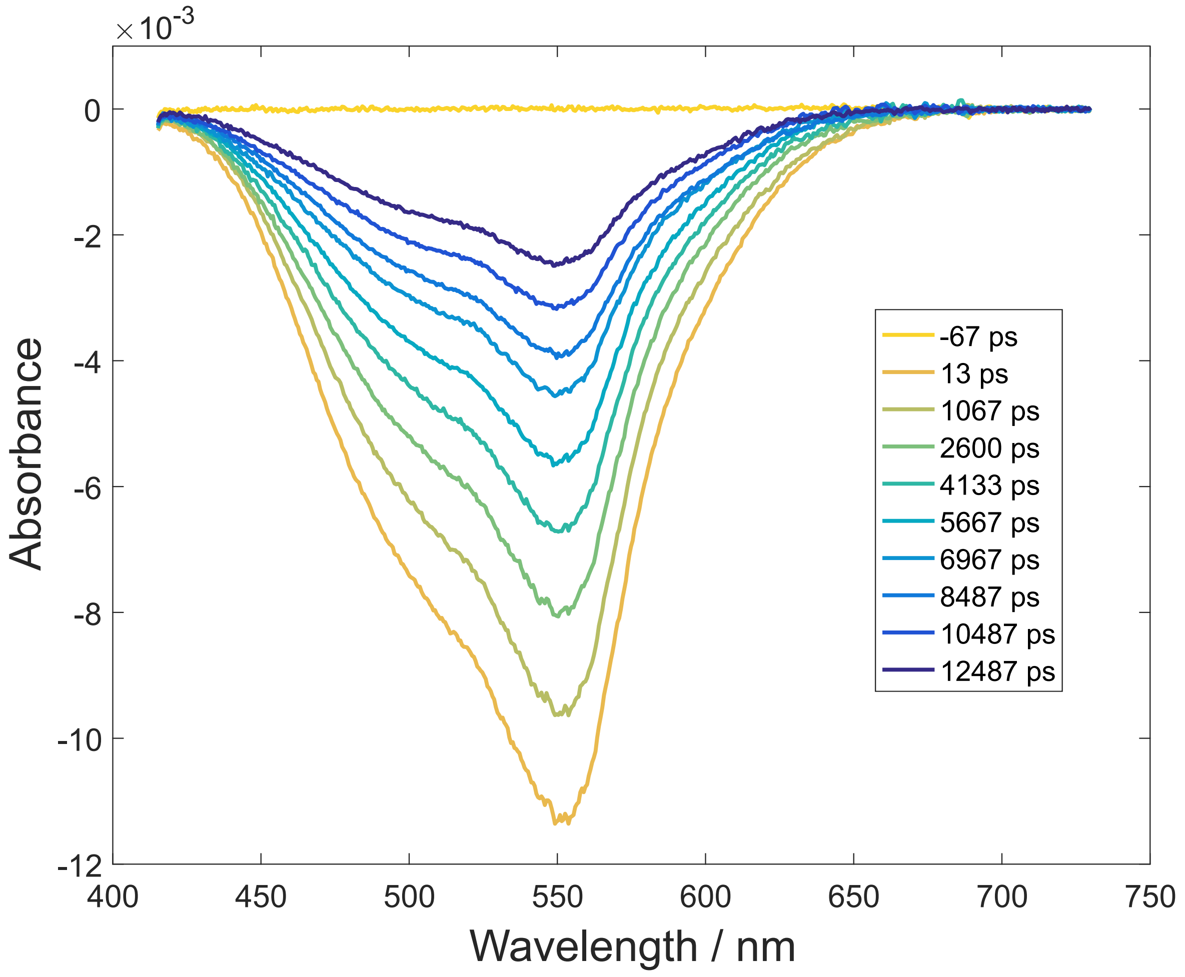

Figure S11: Example transient optical absorption spectroscopy (TOAS) data of the **S2** complex at some selected time delays.

7 Optimized BP86/TZVP level geometries of the studied complexes

7.1 Singlet state

7.1.1 $[Fe(terpy)_2]^{2+}$(**H0**)

See the Supplementary Information of Ref. 2.

7.1.2 $[Fe(3\text{-Cl-terpy})_2]^{2+}$

Fe	0.983740201260	-0.862323704874	-0.232625731668
N	1.253042797859	-0.505601317483	-2.062844684664
N	2.053970599901	-2.446960347699	-0.712008671500
N	-0.005295046616	0.845122592125	-0.336651286640
N	0.715713277869	-1.205539909614	1.599366806196
N	-0.691788953576	-1.895071554557	-0.343448261247
N	2.582184692325	0.065164209800	0.464171418547
H	-1.811608489460	4.231877548457	-0.963885977087
H	5.349760212222	1.851940951165	-0.269295169087
H	5.597247960254	1.761979221528	2.241694021682
H	-0.636571947446	3.062676933372	-2.832950781662
C	-1.307474828050	3.281693690742	-0.789044810315
C	4.618176087308	1.338920021430	0.354290464340
H	-1.802602948397	3.167142543778	1.325797818026
C	-0.651863877037	2.630444241382	-1.832849528788
C	4.751540996310	1.287074265617	1.744032423456
C	-1.304313812887	2.696115816439	0.479374999954
H	0.476049742364	1.865572029174	-4.351772155660
H	3.395014196522	0.743316776398	-1.327941225923
C	3.524729284971	0.718993319520	-0.246945556071
C	0.901655589863	0.953927289891	-3.934920974566
C	-0.007062505028	1.415527460316	-1.585601856167
C	3.782665267252	0.616215865837	2.488946971179
H	3.857858459214	0.559756565099	3.574821362159
C	-0.645597913781	1.482872730433	0.666102312426
C	1.639355233593	0.080402027165	-4.732805075950
C	0.721607592424	0.634604699254	-2.586372352195
H	-0.626667781234	1.004502285376	1.644156585790
C	2.708073004595	0.012239473074	1.830218675968
C	2.183772965608	-1.086255520854	-4.187074370393
H	2.757776962070	-1.757664522794	-4.816505234994
H	2.199452743901	-0.547988469886	4.571352418539
C	1.980854199093	-1.373883151937	-2.830504929721
C	1.625433466706	-0.719599041897	2.489397718013
C	1.470763215170	-0.936806242948	3.861267336377
C	2.447959557506	-2.521539157186	-2.034567155029
C	0.361697324992	-1.660172480280	4.298965411389
C	3.214452069777	-3.631400594555	-2.447475585468
C	-0.379199197290	-1.916316442701	2.010634255342
H	-0.864602575543	-1.815955115070	-2.411027571150
C	2.399439886344	-3.415608230645	0.157290495753
C	-0.569020407015	-2.152809602950	3.379131213862
C	-1.323796996993	-2.184171748975	-1.496587832996
H	2.057530940933	-3.297290644813	1.182993745115
C	-1.207147741609	-2.326200891108	0.863971581700
C	3.569336544064	-4.631933741638	-1.540940384505
C	3.155569775041	-4.520837066666	-0.218205428241
H	-1.428231267229	-2.712954014785	3.731181284527
H	4.161824360085	-5.482225090963	-1.876897261828
C	-2.505583944633	-2.915500304912	-1.536000022368
H	3.413365766477	-5.280325582540	0.518623732955
C	-2.405742535727	-3.069712083395	0.857608625568
H	-2.983008999151	-3.127113360279	-2.491554222388
C	-3.056634200539	-3.365052979932	-0.341371676144
H	-3.982294661712	-3.939178337697	-0.328592276979
H	0.214942587561	-1.845323488580	5.362965341439
H	1.797120438219	0.304603432521	-5.787496692833
Cl	3.774486305295	-3.847429061328	-4.097233512082
Cl	-3.165374673993	-3.673177184356	2.320659236008

7.1.3 $[Fe(4\text{-Cl-terpy})_2]^{2+}$

Fe	0.994569185279	-0.850671203065	-0.228828270622
N	1.264755276685	-0.498078351487	-2.062522265103
N	2.068733301185	-2.445925015716	-0.692836105971
N	0.014108539048	0.864654953702	-0.364428360891
N	0.718813102590	-1.199852427836	1.603863092858
N	-0.688658282543	-1.883905432271	-0.359579392298
N	2.587978370750	0.067060718130	0.501595149031
H	5.362179169395	1.857569992483	-0.198989105473
H	-0.615003509516	3.058580484016	-2.891219997878
C	-1.279505389986	3.286101606603	-0.843218010392
C	4.625670677219	1.341198331717	0.416025021512
H	-1.785059307683	3.212477694589	1.273869238656
C	-0.628593794270	2.634672387976	-1.888406082284
C	4.748308731585	1.281848018071	1.806509521931
C	-1.285651395487	2.728343529813	0.436711456812
H	0.508700354883	1.816446969037	-4.412389468993
H	3.415626735764	0.754890235046	-1.279391332760
C	3.536880131655	0.724692629630	-0.197393136784
C	0.926968308721	0.913761252287	-3.969064029377
C	0.012699017028	1.423913913958	-1.619741959468
C	3.773575601520	0.606824642491	2.540196408734
H	3.839272179266	0.543935686185	3.626470376289
C	-0.626807101589	1.517988344310	0.627657450970
C	1.663512593531	0.016316778623	-4.748338695936
C	0.739484190157	0.631387144939	-2.613223411050
H	-0.613147784219	1.055749473508	1.613521450968
C	2.704271072925	0.006706032679	1.870150192907
C	2.199014602461	-1.139663139460	-4.172271460862
H	2.773450543469	-1.842540550212	-4.774445210681
H	2.147975306270	-0.599137387589	4.609776018375
C	1.984515163416	-1.378615704203	-2.811439414937
C	1.615649716666	-0.729858739123	2.514293372950
C	1.432105089140	-0.973564717597	3.879017604471
C	2.451748144271	-2.512411452684	-2.011738665789
H	3.505757116120	-3.602558341535	-3.548355637796
C	0.312794661154	-1.704255502945	4.290299844170
C	3.216694528094	-3.575932838504	-2.498221991261
C	-0.373903176127	-1.913169519161	1.993491160216
H	-0.949794802332	-1.848176919596	-2.425363566555
C	2.447860162553	-3.439266071398	0.138871917843
C	-0.601979608591	-2.180683474039	3.346120053712
C	-1.367011423304	-2.198384048331	-1.483134730888
H	2.127824515331	-3.357112734669	1.176135672063
C	-1.190645003596	-2.312373221965	0.845806076215

C	3.602831303753	-4.595069329842	-1.628444029375
C	3.211265959297	-4.523803922340	-0.289164589233
H	-1.476073116620	-2.749516392003	3.661003674910
C	-2.547827895045	-2.934018111934	-1.475144415371
H	3.489421591452	-5.295616869054	0.427698826076
C	-2.370076300636	-3.054286630697	0.932965105282
H	-3.052904704274	-3.159749637359	-2.412463903760
H	-2.747203779942	-3.382178907159	1.899934029520
C	-3.047617134163	-3.363184140155	-0.244160155648
H	0.152866050522	-1.903617280546	5.349740651887
H	1.821574168340	0.218996876920	-5.807339639609
Cl	-4.529209182619	-4.292564638811	-0.175823735511
Cl	-2.091924194288	4.807937230791	-1.139501012733
H	4.200462623051	-5.431503765188	-1.991518966796
H	5.589937102282	1.754364490965	2.313297383728

7.1.4 $[Fe(5\text{-}Cl\text{-}terpy)_2]^{2+}$ (H1)

Fe	0.988511883	-0.849815792	-0.228904202
N	1.257794550	-0.484721400	-2.086036671
N	2.085638106	-2.466126570	-0.726393416
N	-0.034759022	0.886867217	-0.387210447
N	0.712618474	-1.188707192	1.631867844
N	-0.725911445	-1.914232692	-0.331132122
N	2.615380329	0.078618589	0.521625219
H	-1.914872921	4.192367862	-1.128563855
H	5.605957856	1.701130463	2.367781317
H	-0.668118469	3.025732698	-2.926565043
C	-1.387340715	3.268883594	-0.923843729
C	4.664854552	1.317858690	0.466244058
C	-0.688758443	2.611065553	-1.926333173
C	4.768322330	1.247246836	1.850448557
C	-1.387273410	2.705006758	0.343942746
H	0.499076372	1.824854310	-4.410472374
H	3.470048798	0.759798467	-1.235761985
C	3.575914536	0.721458169	-0.158319193
C	0.924569027	0.931937019	-3.969806488
C	-0.023881064	1.422630585	-1.641151982
C	3.779875816	0.581469963	2.565922052
H	3.838237211	0.507691648	3.645208544
C	-0.702942186	1.519310683	0.582726418
C	1.683488737	0.056787383	-4.742530956
C	0.729210657	0.634255199	-2.623253621
H	-0.696747421	1.075542771	1.570070063
C	2.712579967	0.008486802	1.880763857
C	2.220245380	-1.095259749	-4.173728569
H	2.806769005	-1.779676754	-4.773565502
H	2.113151303	-0.526847636	4.615760915
C	1.983313915	-1.348650765	-2.824772314
C	1.603530185	-0.710888331	2.525045949
C	1.407693110	-0.911425583	3.889970711
C	2.446080841	-2.503677849	-2.041980924
H	3.454646573	-3.569884580	-3.614811146
C	0.278324631	-1.612328337	4.307287630
C	3.182351066	-3.560886230	-2.566667740
C	-0.385499055	-1.868300700	2.021265645
H	-0.987466935	-1.951369372	-2.387959458
C	2.467987989	-3.472595380	0.072347740
C	-0.630886502	-2.099580077	3.372348034
C	-1.394410200	-2.263647575	-1.435025383
H	2.169534619	-3.409052144	1.111365075
C	-1.211432909	-2.293874026	0.885327220
C	3.566622753	-4.602233922	-1.730628147
C	3.205330192	-4.556556025	-0.389007714
H	-1.509013761	-2.645049058	3.694759152
H	4.139710083	-5.434201228	-2.124119335
C	-2.570422316	-3.001605841	-1.370096222
C	-2.389510206	-3.024965269	1.000650488
H	-2.764922305	-3.313648257	1.974242321
C	-3.087214771	-3.389132271	-0.141891895
H	-4.005887056	-3.958754706	-0.073831142
Cl	-3.385194119	-3.429075865	-2.858278689
Cl	-2.247526340	3.465672111	1.665071135
H	5.411995384	1.824978383	-0.132347894
H	3.486540554	-5.343725813	0.299916537
H	1.858906791	0.274243116	-5.789794225
H	0.104854845	-1.779452079	5.364147709

7.1.5 $[Fe(5,5''\text{-}diCl\text{-}terpy)_2]^{2+}$ (H2)

Fe	0.975727948519	-0.849894921519	-0.230756129434
N	1.244914200067	-0.503148073558	-2.068154257726
N	2.050902703853	-2.446244797416	-0.693125390983
N	-0.011153680066	0.861407903370	-0.372708099828
N	0.706777970472	-1.192338675145	1.606572388562
N	-0.705803595325	-1.886936597350	-0.351867404199
N	2.568036735246	0.076702073498	0.493769714253
H	-1.829426304368	4.240011833328	-1.040223083475
H	5.573941561134	1.783280059584	2.288921817328
H	-0.645458806798	3.056254547491	-2.888457511049
C	-1.321263528229	3.294122026144	-0.858599320823
C	4.591621985194	1.349086037631	0.403166313852
C	-0.658472907136	2.629018630193	-1.886330621149
C	4.731225725885	1.305027535334	1.791180016847
C	-1.311872419098	2.710542250805	0.409135104050
H	0.488903109292	1.807189488890	-4.419969718171
H	3.389783322994	0.759969643470	-1.299728763542
C	3.505657796872	0.730242535781	-0.217775677544
C	0.907713975230	0.905435454493	-3.975510012315
C	-0.010285098534	1.418357893248	-1.629795606972
C	3.757813205047	0.629432501166	2.521990282561
H	3.833666262122	0.575972999093	3.607853976020
C	-0.651985149821	1.499766308621	0.624651788423
C	1.645770331320	0.007359722667	-4.752308711643
C	0.718652741427	0.625481338611	-2.618687742606
H	-0.640106879835	1.038779142567	1.610619068266
C	2.686658544623	0.021499186633	1.862243561209
C	2.182187614390	-1.147950965974	-4.175593006874
H	2.757746640120	-1.850854414977	-4.776555182535
H	2.142116706650	-0.575510864579	4.606664732690
C	1.965734195288	-1.384346100740	-2.814621780438
C	1.604203206842	-0.715699525604	2.512449119920
C	1.426086211112	-0.954811246791	3.878692934702
C	2.431980866547	-2.514818647156	-2.012472952633
H	3.486359832914	-3.619732137978	-3.540972844013
C	0.309961986990	-1.688287023837	4.293836876068
C	3.194434706079	-3.582978816948	-2.492198139218
C	-0.383006536981	-1.907713300456	1.999170721925
H	-0.960387514200	-1.848318441034	-2.422547215305
C	2.423202659561	-3.431001406691	0.146401790620

C	-0.605905260371	-2.171129517809	3.354208089694
C	-1.374199016793	-2.196835311305	-1.478677517937
H	2.108743056220	-3.353968996626	1.185021825031
C	-1.202171609970	-2.312658536042	0.857584807563
C	3.582737475281	-4.603444349094	-1.628289513417
C	3.185446840378	-4.514639711518	-0.293214411212
H	-1.477414742047	-2.741748357013	3.672769168777
H	4.177555392705	-5.443435616301	-1.985099239399
C	-2.556477743957	-2.937954772871	-1.443706562468
C	-2.382028230873	-3.056736212247	0.935079432603
H	-2.759387514161	-3.385160117834	1.902444276875
C	-3.077721456283	-3.379674539734	-0.226687019925
H	-3.999888484934	-3.957659856779	-0.184574762976
Cl	-3.370002281458	-3.301026435095	-2.949611472998
Cl	-2.121910341115	3.481478361005	1.755074139169
H	1.804650775599	0.208967793667	-5.811388734070
H	0.153339083014	-1.884750712856	5.354316516551
Cl	3.631634930772	-5.754890080315	0.857762276757
Cl	5.771281118591	2.178071151902	-0.588701276438

7.1.6 $[Fe(6\text{-}Cl\text{-}terpy)_2]^{2+}$

Fe	0.965338052528	-0.944401807681	-0.251629056753
N	1.332844031937	-0.484668408370	-2.051380311013
N	2.092207881871	-2.584650044206	-0.800298869515
N	-0.048981410905	0.757723219502	-0.354679215106
N	0.658575308305	-1.150007064313	1.605004037957
N	-0.791298621761	-2.025501733328	-0.314085788101
N	2.583977900009	-0.026543410907	0.437680120603
H	-1.897615837491	4.134496843390	-0.956905994096
H	5.451446542152	1.580952726396	-0.334209143669
H	5.623308203516	1.676326786257	2.184707683841
H	-0.549928061894	3.084485895367	-2.782431767823
C	-1.381814524542	3.189299941369	-0.789369047632
C	4.681218090165	1.149199031113	0.304100318585
H	-2.031967470952	2.952703236069	1.272799709499
C	-0.630685946103	2.604788460738	-1.807217142910
C	4.772736214668	1.200204430118	1.696552930033
C	-1.458500256547	2.539084891075	0.444063060399
H	0.770897984239	2.038126203401	-4.235606796074
H	3.491976055540	0.485182658673	-1.367436849532
C	3.579214583195	0.532672740161	-0.284234694792
C	1.165743185307	1.102612669529	-3.842212405385
C	0.026056840763	1.394704441606	-1.569803870233
C	3.752716651550	0.628720508195	2.454476789704
H	3.790045403876	0.650991688821	3.543516708088
C	-0.782641313982	1.334316644522	0.622315026140
C	2.005016754995	0.302393536494	-4.620980461056
C	0.847814827137	0.686757419241	-2.546473288782
H	-0.830917677175	0.814820537402	1.576619928590
C	2.672570705325	0.022060406852	1.807962976722
C	2.518984837199	-0.885043386737	-4.094337986159
H	3.186443364336	-1.506241506374	-4.689736130782
H	2.030123836555	-0.245639317498	4.563432761847
C	2.163720704504	-1.259510778623	-2.793671468677
C	1.543413526916	-0.607688048931	2.485102434896
C	1.315329232744	-0.674366867730	3.862375692107
C	2.590788444310	-2.465720752969	-2.086480178336
H	3.783768781375	-3.287292660019	-3.682004372016
C	0.151027020172	-1.293765219849	4.323630560108
C	3.415652616333	-3.428386590428	-2.667656922004
C	-0.483308176617	-1.737600765066	2.042781445791
C	2.435226552606	-3.707594334924	-0.137201857168
C	-0.763691354862	-1.823857970223	3.410922104107
C	-1.513942386590	-2.516511386632	-1.341400808996
C	-1.310320453261	-2.246955762200	0.950465495849
C	3.758133051574	-4.566917002172	-1.943562708256
C	3.254060310001	-4.713921122378	-0.656212066799
H	-1.679884119688	-2.295644914853	3.762172468329
H	4.402334827369	-5.332838774955	-2.375276168229
C	-2.725824977364	-3.197795678856	-1.201553763200
H	3.481787174451	-5.587370208808	-0.048229098775
C	-2.514093093169	-2.917210308754	1.165204036216
H	-3.244401742858	-3.557248428213	-2.087997674519
H	-2.880419039361	-3.063086808126	2.179618138922
C	-3.237236892694	-3.396194585491	0.076380504956
H	-4.182212528531	-3.919619898539	0.218406920373
H	-0.048140703205	-1.355973059799	5.393452100938
H	2.267533581473	0.608232426291	-5.633050168744
Cl	1.828598788371	-3.968692399883	1.484331677601
Cl	-0.927767277817	-2.316579334746	-2.979224557070

7.1.7 $[Fe(4'\text{-}Cl\text{-}terpy)_2]^{2+}$

Fe	0.000124921958	-0.003479090566	-0.000106140321
N	0.001609549114	0.001136336364	-1.884163473409
N	1.346627651262	-1.421056445390	-0.312629009287
N	-1.345800453403	1.415688488562	-0.307774121420
N	-0.001146506296	-0.007984960692	1.883956337702
N	-1.418160170317	-1.350773368672	0.305832418362
N	1.418031962423	1.342243059183	0.314157105614
C	-3.205918936559	3.380442706359	-1.036677637153
C	3.104941636947	2.944682590207	-0.293358080569
C	-2.522888197739	2.662180014528	-2.016877472449
C	3.378221958752	3.203555074369	1.052067875361
C	-2.945609588194	3.102335999635	0.307478111300
C	2.122741019046	2.012061711091	-0.621393429397
C	-0.836568423106	0.889926636912	-3.947708819533
C	-1.600000992094	1.687195489114	-1.631836274112
C	2.657486834331	2.518434971206	2.028976162324
C	-2.013695586099	2.117948891902	0.630983410283
C	0.004255798431	0.008323669754	-4.632724282910
C	-0.814346445915	0.862424706124	-2.551754797528
C	1.684897107278	1.594892358227	1.639463304587
C	0.843770177267	-0.876836102683	-3.950722063259
C	0.818918207060	-0.856594822758	-2.554692190968
C	0.857699067569	0.807306280257	2.555532847245
C	0.880793046446	0.827366742010	3.951599298746
C	1.603127741698	-1.685883088002	-1.637610040310
C	-0.002947954782	-0.014527779237	4.632518291192
C	2.527035827536	-2.658612361826	-2.025942878344
C	-0.860817806274	-0.826477538247	2.550500137619
C	2.013127582995	-2.127793472865	0.623757615342
C	-0.885755738772	-0.853189646195	3.946419959730
C	-2.121528545336	-2.016319045834	-0.633766527859
C	-1.686781672727	-1.609702445649	1.629572957386
C	3.208623318330	-3.381525101474	-1.048164041290
C	2.945901702940	-3.110255284560	0.296923055028
C	-3.104019897689	-2.950646430617	-0.311451223101
C	-2.659773757856	-2.535195866850	2.013445965314
C	-3.379080355211	-3.215876291180	1.032371348480

H	-3.929549115154	4.145576205793	-1.318835380420
H	3.642226846216	3.454833014801	-1.091803231757
H	4.141504714291	3.927809190961	1.337633244497
H	-2.702643457295	2.855748260678	-3.074120971862
H	-3.454080824087	3.637442445608	1.108445841901
H	-1.487257440923	1.575817071380	-4.487042132343
H	1.889798708486	1.790584172104	-1.661629189259
H	2.847170600319	2.697210868005	3.087096170556
H	-1.790983672537	1.881469606685	1.670142454333
H	1.495531980743	-1.559869132532	-4.492351434663
H	1.564999147115	1.477200412211	4.494080539970
H	2.708842238097	-2.846711964748	-3.083832013314
H	-1.887242138300	-1.789962357151	-1.672633102218
H	1.788566417906	-1.896515183402	1.663700107611
H	-1.570677701462	-1.505614372981	4.484870367532
H	3.933058908330	-4.144965782449	-1.332818866510
H	3.453248309913	-3.649214283341	1.096025343400
H	-3.640113230488	-3.457171829152	-1.112993841818
H	-2.850896135648	-2.718873679433	3.070462596119
H	-4.142602236874	-3.941577399996	1.313613069116
Cl	-0.004150647434	-0.018633299812	6.384445916047
Cl	0.005953775770	0.012921554264	-6.384648425312

7.1.8 [Fe(3'-Cl-terpy)$_2$]$^{2+}$

Fe	0.988752136826	-0.844027676387	-0.228117027246
N	1.260134737860	-0.496902131986	-2.066537417852
N	2.057276157231	-2.448247424947	-0.658570262691
N	0.018527809691	0.857335075195	-0.369510351630
N	0.714216811924	-1.197818936427	1.607706243424
N	-0.691408982425	-1.868948784849	-0.391979860741
N	2.566026653530	0.067045483521	0.502132042634
H	-1.804090320688	4.239911182360	-0.932692929107
H	5.304818821519	1.874635827775	-0.294765558048
H	5.591424230959	1.794728893099	2.216922357358
H	-0.661479173589	3.105715631532	-2.817884605790
C	-1.295859517740	3.289854057885	-0.769752378588
C	4.590449294274	1.358400505623	0.345784611149
H	-1.761698061439	3.153076416709	1.350943840777
C	-0.653202578759	2.653210611181	-1.831529196100
C	4.743953786668	1.311623884656	1.730379020648
C	-1.275118092742	2.695039351992	0.490754535927
Cl	0.346232110490	2.260345007221	-4.858592007321
H	3.338467351459	0.741918655811	-1.304578161619
C	3.490273691054	0.724954438514	-0.226677916747
C	0.952075580174	0.859324189531	-3.999166911852
C	0.004402734541	1.434707546090	-1.621938707979
C	3.794496737634	0.635662813608	2.497015701687
H	3.902143056680	0.593207136405	3.576395057827
C	-0.608672371748	1.482514386273	0.647704737026
C	1.689146725272	-0.061321837134	-4.752866406642
C	0.730720312382	0.637866429655	-2.623826340771
H	-0.572756657329	0.991717577708	1.618692281682
C	2.705911777596	0.013412679287	1.873042031783
C	2.209200858170	-1.199982806276	-4.148218055455
H	2.782462687129	-1.916083754837	-4.734913511587
Cl	2.446300791007	-0.508728969505	5.178860846787
C	1.980262269946	-1.401118334711	-2.785680158030
C	1.618683406260	-0.733401385955	2.526517861167
C	1.382417076442	-1.024392197456	3.886385090229
C	2.442270610987	-2.525766832604	-1.973562056848
H	3.500300140136	-3.630748551483	-3.499545958881
C	0.253069927977	-1.760394358586	4.264920281404
C	3.207959778432	-3.593807411512	-2.450793235661
C	-0.387602219932	-1.916050859733	1.959632504245
H	-0.931735884613	-1.813642604452	-2.458298689103
C	2.433165018868	-3.434462381154	0.182986862073
C	-0.641199471222	-2.210804261347	3.300626147552
C	-1.360182368915	-2.172341279005	-1.524335302876
H	2.111332377333	-3.344670495841	1.218971546802
C	-1.198782674097	-2.303555839276	0.806688019382
C	3.591560805001	-4.605532447661	-1.571733499039
C	3.196974341362	-4.522893098207	-0.233972701461
H	-1.519687842948	-2.782789321555	3.595215485214
H	4.189857403002	-5.445012118882	-1.926411378218
C	-2.543262241273	-2.907985734411	-1.518793414648
H	3.473225231929	-5.288704090763	0.489973211280
C	-2.382018695619	-3.044736624827	0.875108664426
H	-3.040331320551	-3.124248593079	-2.463615878263
H	-2.762885630308	-3.376909273968	1.839938456566
C	-3.064259300033	-3.352071619226	-0.300928303514
H	-3.988651295487	-3.928499132008	-0.266033240729
H	0.082533641034	-1.977493679629	5.318595381475
H	1.853160818675	0.122813068051	-5.813844395488

7.1.9 [Fe(4'-SMe-terpy)$_2$]$^{2+}$(**S0**)

See the Supplementary Information of Ref. 2.

7.1.10 [Fe(5-Cl-4'-SMe-terpy)$_2$]$^{2+}$ (**S1**)

Fe	1.010043536143	-0.831012736610	-0.222975216837
N	1.292990337739	-0.465292893141	-2.053396279843
N	2.081135388213	-2.426287765900	-0.698694629873
N	0.041023963780	0.890408956809	-0.359017831348
N	0.724872002545	-1.192780740032	1.607060592636
N	-0.676497238987	-1.858189156900	-0.364035916643
N	2.602407193317	0.071253376137	0.530409793010
H	-1.747722220367	4.291071623109	-1.004734420252
H	5.600623307497	1.731938974919	2.373512206778
H	-0.565019604240	3.115143229956	-2.856959459019
C	-1.247895854047	3.339633342193	-0.829212175462
C	4.646164389318	1.337877442317	0.467169296472
C	-0.585390234141	2.677864865732	-1.859314621384
C	4.760182511515	1.267033364959	1.857802369355
C	-1.248270978147	2.744257654665	0.433231333840
H	0.558632959750	1.881051964078	-4.369341429733
H	3.442903630543	0.769375833347	-1.239607801569
C	3.558519851330	0.730650934779	-0.157266623855
C	0.976583190180	0.966988138084	-3.952886044930
C	0.052393077904	1.459987158352	-1.611499727771
C	3.778065755446	0.590082699318	2.579901964733
H	3.838288820275	0.519362019641	3.665938969411
C	-0.599522887207	1.526718920828	0.640717315354
C	1.715080759857	0.072832888884	-4.749722983148
C	0.782429021432	0.668412380344	-2.603183361864
H	-0.594534216387	1.056047494143	1.622245720346
C	2.710235206183	-0.000403199780	1.899774360535
C	2.239236525636	-1.098719105209	-4.160168692900
H	2.814763164729	-1.805953509277	-4.757477031709
H	2.154322279271	-0.616984384556	4.610488768867
C	2.012265580481	-1.341294456233	-2.810392291211

C	1.612584665831	-0.739482691096	2.530585611267
C	1.429434587349	-0.991207802777	3.890910428522
C	2.470647079838	-2.485551201484	-2.016247129760
H	3.527753346681	-3.570691194074	-3.554367651356
C	0.302840994588	-1.726903374500	4.304594323465
C	3.233166357133	-3.549104115530	-2.505619442741
C	-0.371522204129	-1.907242742965	1.989501118590
H	-0.914164331510	-1.796179000442	-2.435709002078
C	2.452191030047	-3.427108669637	0.128106965167
C	-0.608766262155	-2.189596333939	3.330130951961
C	-1.339025456804	-2.152327220131	-1.499495526012
H	2.126631269183	-3.349725497499	1.164005667804
C	-1.186623268222	-2.294431244577	0.836526453790
C	3.610659430008	-4.575890009403	-1.640997665536
C	3.212924740260	-4.512017752118	-0.303158046761
H	-1.489142287449	-2.760853867619	3.625296299770
H	4.206510591110	-5.412386881316	-2.006842553645
C	-2.525811192705	-2.885550812409	-1.482296117996
C	-2.372209284172	-3.031059737184	0.895852413634
H	-2.760562571858	-3.367285454794	1.856254136229
C	-3.060864046813	-3.337000134961	-0.274691075566
H	-3.987314864711	-3.908928305859	-0.246451707070
S	-0.062723530375	-2.125370364561	5.988378187948
C	1.311041688677	-1.364985966598	6.926332100221
H	1.313305834335	-0.274807682763	6.808538158670
H	2.274616829091	-1.800699413130	6.637921696879
S	2.042375312840	0.311606894207	-6.471313702019
C	1.233628820389	1.908624823130	-6.848355313273
H	0.152510846491	1.853252774876	-6.671694246512
H	1.691400406647	2.725096004065	-6.278262152395
H	1.101520641178	-1.614832808569	7.973613954812
H	1.417365191215	2.067420674020	-7.917788634668
Cl	-3.330077134767	-3.227974020003	-2.999655363770
Cl	-2.058468298972	3.511019305676	1.783124180504
H	5.388470742002	1.856063943557	-0.139328310211
H	3.484338110189	-5.289932434552	0.409707840150

7.1.11 [Fe(5,5''-diCl-4'-SMe-terpy)$_2$]$^{2+}$ (S2)

Fe	1.013101384130	-0.831214362414	-0.222569672965
N	1.296897759012	-0.464895242925	-2.054105835617
N	2.081690487592	-2.427902665128	-0.700707796912
N	0.040966071731	0.888618235712	-0.359714571589
N	0.728448646257	-1.193528598628	1.608700907715
N	-0.674774426929	-1.856575803187	-0.362896026092
N	2.603759134387	0.073376949367	0.532148028732
H	-1.752959491417	4.285115354230	-1.005768297347
H	5.606343129929	1.737245471110	2.373964213270
H	-0.565171383338	3.113174004469	-2.856902468697
C	-1.251535531729	3.334571100989	-0.829905666035
C	4.637007127549	1.333232012291	0.474633343154
C	-0.586149146832	2.675088826520	-1.859667472563
C	4.764746736984	1.271043837378	1.863072943921
C	-1.253001497438	2.738840138554	0.432180889311
H	0.561493589042	1.880823819869	-4.369341764198
H	3.444615047343	0.773338263598	-1.245103450044
C	3.552259980999	0.729699836264	-0.162752769059
C	0.979895586115	0.967006365327	-3.952881170142
C	0.053510912307	1.458489928420	-1.611760661057
C	3.780426800326	0.592849237635	2.576801622557
H	3.848061833511	0.526294292150	3.662399237448
C	-0.601967981311	1.522158767898	0.639706834337
C	1.719163518121	0.073181461422	-4.750155193616
C	0.785737330213	0.668398499196	-2.603490212276
H	-0.597291632551	1.050987469177	1.620909032238
C	2.710690874448	0.000431384054	1.901540815274
C	2.243000990240	-1.098969954434	-4.161227603754
H	2.817992881657	-1.806274241074	-4.758922477912
H	2.157052582714	-0.614815417321	4.612631731035
C	2.015421162481	-1.341201265428	-2.811555458327
C	1.615525429040	-0.739035985374	2.532344219920
C	1.432760437633	-0.989713824560	3.892838187786
C	2.470872121445	-2.485234347742	-2.018509267484
H	3.528490121661	-3.581132233232	-3.551478688622
C	0.306196414979	-1.725918803995	4.306222086126
C	3.229933709811	-3.552763390701	-2.504276376424
C	-0.368089949466	-1.907441666751	1.990875705019
H	-0.912466283308	-1.792125136193	-2.434483015720
C	2.443841502953	-3.423887143849	0.130658463035
C	-0.605518808674	-2.188902827046	3.331394262376
C	-1.337089327435	-2.148873058548	-1.498473064529
H	2.122579398518	-3.355139775228	1.167783295232
C	-1.183699741276	-2.293668472902	0.837573798646
C	3.607334174613	-4.584443134399	-1.648825338554
C	3.202497564204	-4.506970319785	-0.315294750643
H	-1.486035761688	-2.759765454517	3.626738245535
H	4.199559597165	-5.424017396776	-2.010897767973
C	-2.524157709645	-2.882218062928	-1.481662422982
C	-2.369126357653	-3.030181049995	0.896654847467
H	-2.757043284145	-3.367634105754	1.856766370297
C	-3.058117509122	-3.334644681974	-0.274108901778
H	-3.984459735662	-3.906758490176	-0.245844386442
S	-0.059325358940	-2.124335562450	5.988743763548
C	1.314112196709	-1.364144768623	6.927147373282
H	1.315876442437	-0.273930580771	6.809822113687
H	2.277670787943	-1.799820086294	6.638697784783
S	2.046978696701	0.312880679926	-6.470279071127
C	1.237253081599	1.909310216329	-6.847271192607
H	0.156078007417	1.852762093429	-6.671479011863
H	1.694261969250	2.725823798272	-6.276680449244
H	1.104212880561	-1.614621504559	7.974186266888
H	1.421766025541	2.068262052893	-7.916530950742
Cl	-3.328459569124	-3.222760157330	-2.998419257425
Cl	-2.066456803610	3.502266558786	1.780847867325
Cl	5.830910647101	2.166692502105	-0.496928093342
Cl	3.635178516930	-5.762117584376	0.825529325756

7.2 Triplet state

7.2.1 [Fe(terpy)$_2$]$^{2+}$(H0)

Fe	0.976694689001	-0.847825729525	-0.226292080603
N	1.249386281381	-0.498524696915	-2.084617125952
N	2.134319533072	-2.547746627197	-0.782288070593
N	-0.071219132641	0.995118421066	-0.440548177826
N	0.702504075828	-1.197972120825	1.627699545881
N	-0.828416656377	-1.963448974477	-0.303072836032
N	2.667255059555	0.125601018724	0.607034347747
H	-1.833080191427	4.332951052223	-1.331971368384
H	5.471523607211	1.927861072090	0.075763454938
H	5.578715711974	1.748579474220	2.589784530719
H	-0.615027208121	3.051567728661	-3.086189096074
C	-1.337700492806	3.394655791401	-1.080407506775
C	4.705526078611	1.394792558590	0.638102733362
H	-1.900387437620	3.426669320682	1.021275666481

C	-0.655910655368	2.678625437243	-2.063490078527
C	4.760374830282	1.292574147113	2.031708053168
C	-1.377915986085	2.896469295156	0.225603695300
H	0.487946701136	1.801782307844	-4.455293043612
H	3.560247635302	0.852556941101	-1.120869197844
C	3.641708325111	0.796687241284	-0.034797019813
C	0.910605090004	0.901257252766	-4.012041501236
C	-0.027444044405	1.475861025399	-1.716283489832
C	3.755390697558	0.599689027480	2.705403900361
H	3.781667169250	0.510129479700	3.790777920465
C	-0.731488338387	1.692987827759	0.501918259845
C	1.646012970101	0.009636944409	-4.794514364244
C	0.721920183641	0.628939515127	-2.653718356781
H	-0.738328554107	1.268370140456	1.506499048542
C	2.714597155647	0.022455238415	1.967010391438
C	2.180811485424	-1.138783868908	-4.208729773745
H	2.756851758107	-1.843679486869	-4.806451116281
H	2.132097053191	-0.601106836650	4.642655765338
C	1.971023157597	-1.376634517269	-2.847048869985
C	1.597737040974	-0.730437468504	2.551480727526
C	1.414142504177	-0.977203792883	3.915253025579
C	2.473536062535	-2.539380227434	-2.103396559379
H	3.501400811714	-3.551962166555	-3.722820498802
C	0.302604783420	-1.709396240946	4.335916661062
C	3.237284781128	-3.569991021341	-2.666091280083
C	-0.389197267388	-1.916635091316	2.034118666953
H	-1.165846609861	-1.975615024330	-2.351740419546
C	2.543433415776	-3.568113709680	-0.005907128937
C	-0.607029011937	-2.183258319636	3.389109396048
C	-1.549258265316	-2.307870173597	-1.386341430149
H	2.247285717496	-3.530590990983	1.043117605294
C	-1.257830572805	-2.350758877069	0.932879065339
C	3.656683100162	-4.622850947705	-1.853935968552
C	3.305626471820	-4.625078625675	-0.500311779413
H	-1.478890378722	-2.755753455866	3.702461547466
H	4.252205043991	-5.433966740073	-2.274089970460
C	-2.725594380231	-3.049732970616	-1.296449455393
H	3.614205394514	-5.429640254413	0.166494161471
C	-2.431595556514	-3.097631553303	1.095187733906
H	-3.275893830079	-3.305327453999	-2.201424691776
H	-2.763799419124	-3.400529179932	2.087477039092
C	-3.171586372691	-3.449652864382	-0.032787878710
H	-4.087960285424	-4.031003645078	0.073821830184
H	0.145998716247	-1.909400556146	5.395594940639
H	1.801306580002	0.208556511562	-5.854595421287

7.2.2 [Fe(3-Cl-terpy)$_2$]$^{2+}$

Fe	0.961608887401	-0.854609311411	-0.200725721970
N	1.238904455484	-0.504664969797	-2.072743919416
N	2.046863388843	-2.608699756747	-0.831525435990
N	-0.140300670450	0.951569222607	-0.411605370033
N	0.681996626180	-1.212416713487	1.639484390030
N	-0.817593546020	-1.967835935405	-0.265665536937
N	2.665522349387	0.031121774441	0.554101166894
H	-1.771717299500	4.366537894727	-1.247023695101
H	5.483011935020	1.781977929195	-0.064384485785
H	5.627284415015	1.660964332751	2.450867175141
H	-0.580611181449	3.083027209689	-3.014836906025
C	-1.315889605489	3.404864995260	-1.011009488127
C	4.718608014396	1.273726259743	0.522524642856
H	-1.899844034272	3.412091896768	1.084929576827
C	-0.649882553630	2.685659625115	-2.002868110510
C	4.794067961309	1.203235085208	1.916782363853
C	-1.387434623247	2.881410521614	0.283327977939
H	0.285077412884	1.741807542598	-4.428292937683
H	3.538884908235	0.709300996713	-1.205841748979
C	3.637435040988	0.675830787015	-0.120731752719
C	0.756824052800	0.859630348598	-3.998855012437
C	-0.068587435002	1.454270742366	-1.675582146016
C	3.789707933610	0.542099669101	2.622913815133
H	3.829946199109	0.480990581460	3.710084685133
C	-0.784878031430	1.651510483641	0.540415903758
C	1.448176337928	-0.037613240423	-4.808326801791
C	0.658213427900	0.602035433660	-2.628565830774
H	-0.818030330932	1.205852542067	1.535483857662
C	2.730930349299	-0.039920908916	1.917202580285
C	2.052997676363	-1.158023806014	-4.236968796340
H	2.583958669475	-1.864296353018	-4.866711313694
H	2.223765916804	-0.640715809401	4.607280281012
C	1.962225312759	-1.371546073544	-2.855545215433
C	1.614391446647	-0.763526883956	2.535768696821
C	1.474761401158	-1.002565558949	3.904289868051
C	2.540761538404	-2.503256794639	-2.101724253360
C	0.365006676961	-1.715506603307	4.354140236600
C	3.514377909697	-3.435714297097	-2.524414775533
C	-0.422452312817	-1.909746228529	2.071242110142
H	-1.013270454551	-1.963192768935	-2.329083132038
C	2.425719137617	-3.604409299181	-0.017331057712
C	-0.584372094454	-2.171527630286	3.438643597582
C	-1.477066529951	-2.273527149827	-1.393529260638
H	1.979728443346	-3.617330234073	0.977086929798
C	-1.310289073401	-2.315594743424	0.966214780278
C	3.901872408410	-4.478713246792	-1.679757627246
C	3.340790390738	-4.577699482157	-0.409926580393
H	-1.443733065880	-2.731011243907	3.791222687369
H	4.649279145869	-5.196373492836	-2.017939706984
C	-2.690345578967	-2.953510003947	-1.382588887000
H	3.618634404905	-5.384001620522	0.267872883759
C	-2.550610970688	-2.992321475382	1.022160229806
H	-3.199267050330	-3.193071253078	-2.315096820055
C	-3.233733911317	-3.312416764545	-0.152727049213
H	-4.187769368404	-3.835479654119	-0.094453543148
H	0.237364699898	-1.922733057812	5.416354504958
H	1.513940227854	0.127783086773	-5.883649902330
Cl	4.340137315872	-3.353021203586	-4.076607970255
Cl	-3.339749696381	-3.471122392058	2.519051849981

7.2.3 [Fe(4-Cl-terpy)$_2$]$^{2+}$

Fe	0.994108789682	-0.849578858354	-0.230151802874
N	1.273725283125	-0.489302794127	-2.084121124030
N	2.133969937937	-2.548621696171	-0.784908652594
N	-0.060135374648	0.989693747870	-0.443079513252
N	0.720506930308	-1.209485298017	1.624789844396
N	-0.828817681059	-1.952117155416	-0.302377139330
N	2.679025040533	0.131419369507	0.607231575042
H	5.484701392887	1.933981313353	0.084113063869
H	-0.555941403210	3.079192408198	-3.079290796237
C	-1.298059079671	3.390153386155	-1.073394943276
C	4.720614122616	1.395061056800	0.643764105740
H	-1.900830969782	3.419471425077	1.020363072133
C	-0.611022072577	2.691625494212	-2.063523536380
C	4.784004332150	1.273022631655	2.035168157793
C	-1.369566175120	2.892130811584	0.230114314270

H	0.548426681377	1.831979066986	-4.447136106573
H	3.563634402736	0.881594158138	-1.115974834243
C	3.651054572943	0.809176425175	-0.031135744911
C	0.961770375761	0.926929248130	-4.004806949322
C	0.003286558459	1.484072133406	-1.711933860017
C	3.780881219862	0.573535974599	2.705172248858
H	3.812707018706	0.469094927034	3.789423865668
C	-0.731413081587	1.683695673347	0.493938559938
C	1.702709206512	0.038237426095	-4.785080005859
C	0.758132488302	0.644350792364	-2.651247921427
H	-0.761726614000	1.255049378607	1.496245025668
C	2.734867722943	0.009138943147	1.965077088466
C	2.227120245787	-1.116268949784	-4.201709230063
H	2.807745628433	-1.817969992438	-4.798922279938
H	2.163305193217	-0.632887682800	4.637356883566
C	2.001517270355	-1.363933705357	-2.844429203629
C	1.619386896525	-0.748009281477	2.547520942721
C	1.441752219234	-1.003742493210	3.910645246926
C	2.493178152721	-2.531563492832	-2.101147056992
H	3.547716948552	-3.532118678683	-3.710407524600
C	0.331308801348	-1.737449207931	4.331914469569
C	3.267241941307	-3.557019209477	-2.658150772761
C	-0.369734696933	-1.928773596788	2.033151908618
H	-1.196608586976	-1.950016595491	-2.347940636192
C	2.531906225751	-3.573674076561	-0.008587980183
C	-0.583361533764	-2.203902792792	3.386661646021
C	-1.562029758047	-2.289429607692	-1.378664231435
H	2.219138657566	-3.543364170889	1.035477960764
C	-1.244278574649	-2.354106815182	0.932074671859
C	3.675794619569	-4.614311018729	-1.845933336804
C	3.303484173073	-4.626145288783	-0.498361411777
H	-1.454246145651	-2.776742342955	3.701832077932
C	-2.735131433132	-3.033911472437	-1.293511972071
H	3.602948444040	-5.434335304565	0.168117394585
C	-2.411214138922	-3.106347357636	1.107550750080
H	-3.296805620457	-3.282748573598	-2.192004924639
H	-2.735083052015	-3.422959516544	2.096992673794
C	-3.152294489379	-3.440550797094	-0.023456694924
H	0.179499324416	-1.944684461894	5.390944589133
H	1.871498816335	0.244586310789	-5.841604739171
Cl	-4.619985775550	-4.378828112281	0.152101238057
Cl	-2.080328732077	4.905559169287	-1.470458262873
H	4.279916179146	-5.421568017545	-2.261618431065
H	5.607285174994	1.718888144018	2.594247243978

7.2.4 $[Fe(5\text{-}Cl\text{-}terpy)_2]^{2+}$ (H1)

Fe	0.977139081510	-0.859733250653	-0.233022031673
N	1.245755981077	-0.498986195377	-2.090078239585
N	2.151089558673	-2.530049830322	-0.777867768676
N	-0.052611780587	1.005877212724	-0.439529461167
N	0.714970246844	-1.220954226834	1.623820295873
N	-0.841653246497	-1.936533659025	-0.306421158148
N	2.659988471698	0.148045629244	0.616333764574
H	-1.823306465604	4.343286510187	-1.326587001250
H	5.609610900981	1.699077714188	2.606501194821
H	-0.616394995741	3.062177335040	-3.080553797477
C	-1.326745707561	3.405125002603	-1.081751501045
C	4.692122019863	1.398714623689	0.663948074360
C	-0.648879711981	2.683497918037	-2.059634572850
C	4.779312581854	1.262821214760	2.051727877362
C	-1.353286702835	2.895396441631	0.218848262949

H	0.498112166426	1.810592031013	-4.456395851912
H	3.533393035259	0.925203910626	-1.108609625448
C	3.620744438305	0.830242181187	-0.026021353545
C	0.916513428826	0.907913797095	-4.014049900737
C	-0.016473595524	1.480834860573	-1.717155208534
C	3.778128814180	0.554355494242	2.709362148714
H	3.825104635645	0.435336058105	3.791357798387
C	-0.706497306978	1.693468447532	0.509316426997
C	1.648912682144	0.014743256278	-4.796440374333
C	0.725720720212	0.633232646149	-2.655966455774
H	-0.714152321539	1.278270690128	1.517465068655
C	2.723278995355	0.002119718045	1.970827883022
C	2.182222995095	-1.135609440031	-4.211994217467
H	2.758425797891	-1.839430633526	-4.810899097078
H	2.156401438658	-0.654647781468	4.640000525142
C	1.969600083559	-1.375472924007	-2.851552973517
C	1.612576366425	-0.761189871614	2.548516929199
C	1.436662543647	-1.024467718440	3.910970376089
C	2.474669990438	-2.534790405523	-2.103901476642
H	3.482987116841	-3.563665880848	-3.724264914966
C	0.328335332445	-1.763642419799	4.327027304029
C	3.230490450496	-3.571397877274	-2.664641601374
C	-0.374661523747	-1.943862868892	2.026794804605
H	-1.204690201551	-1.898683137303	-2.350320144773
C	2.565694550604	-3.544805378447	0.003556755276
C	-0.586222357131	-2.226392719466	3.379211644039
C	-1.571355650328	-2.261174777982	-1.389850246962
H	2.281041296747	-3.497355718989	1.055017876063
C	-1.250264334691	-2.362317543003	0.924221040856
C	3.657881177276	-4.617052520089	-1.846916685479
C	3.321264542667	-4.606969732902	-0.490084899551
H	-1.456963134631	-2.801539380958	3.690372146886
H	4.248533432516	-5.432554401009	-2.265815527778
C	-2.737725424213	-3.019195305992	-1.304547049909
H	3.636798806012	-5.405088350009	0.181039172137
C	-2.411388096588	-3.128382465949	1.080710143532
H	-3.296726110210	-3.256262864705	-2.209005912228
H	-2.725677039375	-3.463377400130	2.068071520267
C	-3.162147350395	-3.458588303113	-0.046977755949
H	-4.070079179830	-4.053552536662	0.055785937630
Cl	5.916623279502	2.279326745416	-0.227461224845
Cl	-2.189998210826	3.751100706844	1.498197780079
H	0.176677189244	-1.976786611111	5.384938905911
H	1.806817729447	0.215312266116	-5.855836986783

7.2.5 $[Fe(5,5\text{-}diCl\text{-}terpy)_2]^{2+}$ (H2)

Fe	0.979322000859	-0.854113739496	-0.231486002678
N	1.255145469630	-0.493291218535	-2.088606157253
N	2.133042859894	-2.550692518561	-0.794190112320
N	-0.071561078382	0.985958945944	-0.437944578864
N	0.711006160141	-1.211884675540	1.625927273486
N	-0.831350691100	-1.969758357029	-0.302756554266
N	2.672726902851	0.122546885042	0.604847801923
H	-1.809045078427	4.346528538498	-1.299944932156
H	5.592772971828	1.733727741579	2.589883905685
H	-0.583781638046	3.085164161983	-3.054970425248
C	-1.321555011365	3.401959616934	-1.061681107338
C	4.700066946251	1.381588726920	0.644487870098
C	-0.633036174339	2.691463919238	-2.040474712782
C	4.771023494597	1.280416634901	2.036051644766
C	-1.370456955337	2.873283817555	0.230537434206

H	0.522544002650	1.834478040653	-4.441193195094
H	3.563685187198	0.859972994288	-1.128965270633
C	3.638285094472	0.792660368984	-0.043514194485
C	0.936977380100	0.927872438313	-4.003269274299
C	-0.012555979159	1.480638017036	-1.707671309904
C	3.764560499509	0.584891296067	2.699654593434
H	3.798645592786	0.494170895395	3.784889625164
C	-0.735079956597	1.663041568664	0.512119373100
C	1.674280527088	0.040257252637	-4.788042766901
C	0.737638290950	0.642399500317	-2.649006342528
H	-0.760742718094	1.232233292568	1.513206140162
C	2.720747453803	0.010247299479	1.963579274129
C	2.201326603556	-1.116003003366	-4.210785866570
H	2.779192819807	-1.816022320037	-4.812460719418
H	2.149614169158	-0.623314464846	4.638683437151
C	1.980136685963	-1.365971579206	-2.852926690816
C	1.607833900169	-0.745640423515	2.547640644233
C	1.430529122053	-0.998051236384	3.911631893334
C	2.475921412681	-2.532618494498	-2.114292215205
H	3.513559913262	-3.545694283414	-3.730403245216
C	0.321649251250	-1.734584246408	4.331980895326
C	3.240971469402	-3.562818681003	-2.676090720491
C	-0.377459817638	-1.933978799133	2.032728869405
H	-1.179702255858	-1.967193095388	-2.356377133613
C	2.530442677873	-3.568762271485	-0.015050676961
C	-0.590604429321	-2.206952055239	3.387430565822
C	-1.548812445995	-2.301805093918	-1.387203624382
H	2.234372386698	-3.545019592275	1.033428587018
C	-1.249356962236	-2.364154612325	0.934300715414
C	3.658370408215	-4.622447860591	-1.875970902786
C	3.293809352217	-4.619004054373	-0.527308511681
H	-1.459649013877	-2.782644732978	3.702338969202
H	4.254406272878	-5.433683808807	-2.293342055480
C	-2.723782312659	-3.047406924992	-1.278734745986
C	-2.420760544446	-3.114618718678	1.095811070477
H	-2.751437431165	-3.427883699429	2.084737780339
C	-3.172075165941	-3.463248516659	-0.022575278317
H	-4.087315515009	-4.045024475385	0.081667299762
Cl	5.931971534130	2.241860983348	-0.254488813632
Cl	-2.221543670869	3.712782869623	1.509675212295
H	0.169403549216	-1.940232243395	5.391271431236
H	1.839071025453	0.249348463455	-5.844661976272
Cl	-3.620614710655	-3.450110889478	-2.727176258690
Cl	3.783635043926	-5.920894997053	0.535920191097

7.2.6 [Fe(6-Cl-terpy)$_2$]$^{2+}$

Fe	0.965338052528	-0.944401807681	-0.251629056753
N	1.332844031937	-0.484668408370	-2.051380311013
N	2.092207881871	-2.584650044206	-0.800298869515
N	-0.048981410905	0.757723219502	-0.354679215106
N	0.658575308305	-1.150007064313	1.605004037957
N	-0.791298621761	-2.025501733328	-0.314085788101
N	2.583977900009	-0.026543410907	0.437680120603
H	-1.897615837491	4.134496843390	-0.956905994096
H	5.451446542152	1.580952726396	-0.334209143669
H	5.623308203516	1.676326786257	2.184707683841
H	-0.549928061894	3.084485895367	-2.782431767823
C	-1.381814524542	3.189299941369	-0.789369047632
C	4.681218090165	1.149199031113	0.304100318585
H	-2.031967470952	2.952703236069	1.272799709499
C	-0.630685946103	2.604788460738	-1.807217142910

C	4.772736214668	1.200204430118	1.696552930033
C	-1.458500256547	2.539084891075	0.444063060399
H	0.770897984239	2.038126203401	-4.235606796074
H	3.491976055540	0.485182658673	-1.367436849532
C	3.579214583195	0.532672740161	-0.284234694792
C	1.165743185307	1.102612669529	-3.842212405385
C	0.026056840763	1.394704441606	-1.569803870233
C	3.752716651550	0.628720508195	2.454476789704
H	3.790045403876	0.650991688821	3.543516708088
C	-0.782641313982	1.334316644522	0.622315026140
C	2.005016754995	0.302393536494	-4.620980461056
C	0.847814827137	0.686757419241	-2.546473288782
H	-0.830917677175	0.814820537402	1.576619928590
C	2.672570705325	0.022060406852	1.807962976722
C	2.518984837199	-0.885043386737	-4.094337986159
H	3.186443364336	-1.506241506374	-4.689736130782
H	2.030123836555	-0.245639317498	4.563432761847
C	2.163720704504	-1.259510778623	-2.793671468677
C	1.543413526916	-0.607688048931	2.485102434896
C	1.315329232744	-0.674366867730	3.862375692107
C	2.590788444310	-2.465720752969	-2.086480178336
H	3.783768781375	-3.287292660019	-3.682004372016
C	0.151027020172	-1.293765219849	4.323630560108
C	3.415652616333	-3.428386590428	-2.667656922004
C	-0.483308176617	-1.737600765066	2.042781445791
C	2.435226552606	-3.707594334924	-0.137201857168
C	-0.763691354862	-1.823857970223	3.410922104107
C	-1.513942386590	-2.516511386632	-1.341400808996
C	-1.310320453261	-2.246955762200	0.950465495849
C	3.758133051574	-4.566917002172	-1.943562708256
C	3.254060310001	-4.713921122378	-0.656212066799
H	-1.679884119688	-2.295644914853	3.762172468329
H	4.402334827369	-5.332838774955	-2.375276168229
C	-2.725824977364	-3.197795678856	-1.201553763200
H	3.481787174451	-5.587370208808	-0.048229098775
C	-2.514093093169	-2.917210308754	1.165204036216
H	-3.244401742858	-3.557248428213	-2.087997674519
H	-2.880419039361	-3.063086808126	2.179618138922
C	-3.237236892694	-3.396194585491	0.076380504956
H	-4.182212528531	-3.919619898539	0.218406920373
H	-0.048140703205	-1.355973059799	5.393452100938
H	2.267533581473	0.608232426291	-5.633050168744
Cl	1.828598788371	-3.968692399883	1.484331677601
Cl	-0.927767277817	-2.316579334746	-2.979224557070

7.2.7 [Fe(4'-Cl-terpy)$_2$]$^{2+}$

Fe	1.016813426024	-0.792009029562	-0.215174453282
N	1.276300587019	-0.414141267170	-2.066949768569
N	2.163291530154	-2.482478758803	-0.804565572040
N	-0.027227002811	1.052665145164	-0.393166703369
N	0.762413615496	-1.168403993590	1.637813460720
N	-0.797037037249	-1.904202401580	-0.282117897751
N	2.720465761038	0.170681588646	0.621581120027
H	-1.755199753467	4.428647190405	-1.203806548570
H	5.525672097728	1.973007473638	0.096430107738
H	5.656541231652	1.749313001483	2.605158200699
H	-0.558655167075	3.175328556528	-2.990461510246
C	-1.271559748523	3.478848026220	-0.975400550567
C	4.762910840388	1.432888503549	0.656724798731
H	-1.828974681508	3.467411700660	1.127327810349
C	-0.600576920259	2.777764047929	-1.976847133431

C	4.831168338264	1.306070951821	2.047206163223
C	-1.314640851735	2.950448300175	0.318053281717
H	0.515643325826	1.936775337692	-4.394009337727
H	3.600003275470	0.926379282777	-1.101367788807
C	3.690573856569	0.850141412798	-0.017103492409
C	0.933480075160	1.028438228406	-3.965074109790
C	0.015972664439	1.562323314860	-1.657697146457
C	3.829670982435	0.604800398539	2.718125908861
H	3.865956776404	0.497103607460	3.801947940858
C	-0.679484120424	1.734767153809	0.566050704236
C	1.663063794622	0.138393501064	-4.753410696125
C	0.754441572282	0.726155559586	-2.613631769761
H	-0.690315040383	1.287702894257	1.560854923883
C	2.781857556800	0.044457604114	1.978376571678
C	2.202398028169	-1.025310844413	-4.204640338938
H	2.772467707983	-1.717394049547	-4.821281989593
H	2.228367843350	-0.614899237149	4.649208631296
C	1.992391104178	-1.279411363759	-2.847757642849
C	1.666855138607	-0.716054127705	2.558859357593
C	1.505371225236	-0.977389240828	3.921084984319
C	2.497257438981	-2.456152141242	-2.126912256122
H	3.517865186784	-3.444956811986	-3.766206840829
C	0.393131766600	-1.714761133710	4.328287427364
C	3.257403661370	-3.477466327585	-2.708941833548
C	-0.324607484584	-1.891099228601	2.047094500104
H	-1.162670204322	-1.885176933246	-2.325274634013
C	2.573184261065	-3.515170569153	-0.045584508607
C	-0.534824817693	-2.180983861818	3.396796598061
C	-1.532543540551	-2.231707770894	-1.360041932768
H	2.280892744058	-3.493224092969	1.004619060406
C	-1.210261967015	-2.311001973077	0.952348743175
C	3.678749016266	-4.542940460736	-1.913916163492
C	3.332377747271	-4.565337802695	-0.559839809708
H	-1.401660536477	-2.756349720452	3.715736088128
H	4.272350428625	-5.348060553812	-2.348319543060
C	-2.708172036588	-2.974788534940	-1.264607591359
H	3.642596359048	-5.380160036187	0.093512608123
C	-2.380431742998	-3.060196796620	1.121115500736
H	-3.270924036703	-3.216051421133	-2.165649365079
H	-2.699636826050	-3.378832774747	2.112391249003
C	-3.136531558114	-3.394602008074	-0.001870371326
H	-4.051722071691	-3.976713247219	0.108961159232
Cl	0.159231210462	-2.058140763670	6.029245401668
Cl	1.906280950404	0.486684467091	-6.451987721734

C	4.677464590446	1.354222355814	1.910210502859
C	-1.154337017086	2.991125938798	0.347348812740
Cl	-0.020731117373	2.039918576126	-4.940256785767
H	3.343035670087	0.828273518546	-1.163801140457
C	3.478632504428	0.797827375111	-0.083012998331
C	0.770039983617	0.752268585654	-4.050198392051
C	0.041194926834	1.523888859642	-1.678079752834
C	3.720852644320	0.653998761507	2.646314396185
H	3.814327506667	0.593103516013	3.725465063539
C	-0.740846505675	1.672330343722	0.524662754681
C	1.474242738394	-0.180689686001	-4.815437883260
C	0.685937361047	0.612639194934	-2.648918133294
H	-0.879123260694	1.164323397037	1.480326622931
C	2.650297082863	0.034800103778	1.985905358117
C	2.071471250181	-1.265997602113	-4.189886204643
H	2.623434538589	-1.994728661438	-4.780828031001
Cl	2.329983727886	-0.491436261274	5.266755794775
C	1.930570389250	-1.422068918470	-2.808661980950
C	1.554373274909	-0.738745882942	2.596143856458
C	1.304657596682	-1.033131656661	3.952847338788
C	2.485216886284	-2.554000822533	-2.050178892910
H	3.606576117313	-3.496706460949	-3.654481467785
C	0.190774766372	-1.794496047726	4.323508136094
C	3.320213497835	-3.533985692012	-2.604264293812
C	-0.420600414834	-1.969208446175	2.007824207804
H	-1.065608053111	-1.954419626901	-2.396582137933
C	2.586953631479	-3.579378978918	0.044284251868
C	-0.676725849635	-2.265194118492	3.347290829623
C	-1.468775878396	-2.312617627056	-1.449658672990
H	2.271827704045	-3.561043597175	1.088398939197
C	-1.245728633861	-2.397849901563	0.877085149932
C	3.788841470206	-4.561857059665	-1.786844301213
C	3.417509704412	-4.589482691329	-0.439330606073
H	-1.544808185466	-2.857418062348	3.631769235732
H	4.441179838389	-5.332938363248	-2.198428032713
C	-2.633619696342	-3.075216665044	-1.407898469214
H	3.762283759879	-5.376980290445	0.230148660260
C	-2.410510501128	-3.167165104850	0.989403282666
H	-3.151828872493	-3.321310816114	-2.333848811304
H	-2.766592867774	-3.494817322901	1.964904103934
C	-3.110022360849	-3.508704891239	-0.166489889961
H	-4.019326706162	-4.106443623352	-0.098866121459
H	0.012179096883	-2.012583009440	5.375414355201
H	1.537021202981	-0.059751910234	-5.896347532716

7.2.8 [Fe(3'-Cl-terpy)$_2$]$^{2+}$

Fe	0.960093103327	-0.858604526273	-0.179568892738
N	1.239077154984	-0.505337666058	-2.069281675607
N	2.131029413605	-2.585009693197	-0.737975040341
N	-0.154776447309	0.958887587691	-0.451844786226
N	0.670138808633	-1.222869869005	1.654816025232
N	-0.786175795165	-1.978941754422	-0.338231396599
N	2.550653643285	0.119454894948	0.618192261621
H	-1.187335985305	4.638352694965	-1.058187043277
H	5.284678076093	1.969722395072	-0.087386287866
H	5.509563945639	1.834295304539	2.426007934372
H	-0.101824371663	3.337605263060	-2.861647091206
C	-0.913328360069	3.597180075717	-0.886436813672
C	4.559506361459	1.432298345733	0.522693448419
H	-1.635737163605	3.526450488540	1.165165023359
C	-0.308168925305	2.863094730617	-1.907801786185

7.2.9 [Fe(4'-SMe-terpy)$_2$]$^{2+}$(S0)

Fe	1.005662150408	-0.826446531372	-0.224186455872
N	1.293893128732	-0.459317255719	-2.075515608229
N	2.158691737384	-2.522961289423	-0.790168636260
N	-0.022123520550	1.026032539218	-0.435765143208
N	0.724045301640	-1.197427622612	1.627767985885
N	-0.817156461286	-1.927763637631	-0.308824236769
N	2.694702606192	0.138817886719	0.644199287638
H	-1.726652273212	4.401407420460	-1.298410331134
H	5.509675237094	1.939565442424	0.160459760158
H	5.604653742792	1.711333091306	2.671106109505
H	-0.505758304210	3.131984779978	-3.055081029615
C	-1.249817381722	3.451780642249	-1.055036866244
C	4.738513955222	1.399182724791	0.709031622333
H	-1.841483650587	3.457442303319	1.038929311757
C	-0.565245225497	2.740962110626	-2.039779370080

C	4.786831464042	1.269803681561	2.100619462169
C	-1.315664917609	2.932903458584	0.241879547536
H	0.580162034519	1.885583895796	-4.408904630037
H	3.598005557582	0.895794696707	-1.064925791402
C	3.674905169512	0.818528013113	0.020505770665
C	0.994777312513	0.970003959051	-3.993334432192
C	0.043144270804	1.525065232690	-1.703995937461
C	3.775220908018	0.568616229306	2.755497854794
H	3.797122603091	0.459431262368	3.839509648444
C	-0.687718471782	1.717691506455	0.507820497203
C	1.729563517130	0.080295774098	-4.793015805144
C	0.792433573982	0.677752502517	-2.643369363810
H	-0.714309887785	1.276733745683	1.505234076882
C	2.735763531726	0.009530361088	2.001838992577
C	2.245334649851	-1.087011363206	-4.195033967633
H	2.822024057861	-1.796459590161	-4.788136220940
H	2.156850049184	-0.637755867826	4.641999595913
C	2.016557935795	-1.332262376277	-2.846042716704
C	1.610394548516	-0.750336267722	2.566177282755
C	1.429303234612	-1.011724538613	3.925286797509
C	2.506501942248	-2.507521659387	-2.108956521146
H	3.541403827790	-3.515348922412	-3.727547849653
C	0.312560469006	-1.751209916355	4.346997826193
C	3.268675583968	-3.538179767174	-2.673079332007
C	-0.370306500634	-1.918644388135	2.028427270843
H	-1.157654410321	-1.903553646527	-2.355532560206
C	2.555883534661	-3.551758965953	-0.018297102955
C	-0.595410673326	-2.206336414512	3.369767614595
C	-1.540373128832	-2.253046902114	-1.396242510924
H	2.251677479789	-3.518394766525	1.028345053494
C	-1.243598335351	-2.336263977165	0.920424265014
C	3.676711961594	-4.599022269159	-1.865414311915
C	3.315736180647	-4.609533139863	-0.514575345983
H	-1.472898808833	-2.782189226291	3.663144395425
H	4.271612909283	-5.410074120256	-2.287094294534
C	-2.716898851157	-2.995828991957	-1.317163572129
H	3.615601801497	-5.420329929881	0.148638033511
C	-2.416620536005	-3.085785632352	1.072274297378
H	-3.269373396424	-3.235358080199	-2.225072175969
H	-2.747512285854	-3.406591826299	2.059069033602
C	-3.159728424860	-3.417869872455	-0.059701997976
H	-4.076290945065	-4.000116964937	0.039549504336
S	-0.043518605779	-2.160312235380	6.030458914173
C	1.335686654019	-1.403197172549	6.963627999843
H	1.335751772720	-0.312388215545	6.851542482446
H	2.297711893272	-1.836207751885	6.666147338409
S	2.061048128969	0.317304415547	-6.514345514038
C	1.253792583652	1.915120373485	-6.892449663559
H	0.172524613955	1.860556948040	-6.716281331207
H	1.711996182849	2.731233615920	-6.322173546820
H	1.134046405512	-1.658619974821	8.011068636874
H	1.437984183050	2.073976287547	-7.961775826106

7.2.10 [Fe(5-Cl-4'-SMe-terpy)$_2$]$^{2+}$ (S1)

Fe	1.007317186601	-0.827985234947	-0.223363910099
N	1.290640989105	-0.459102719975	-2.077434683947
N	2.187661336679	-2.495984076404	-0.771833145925
N	0.000977116037	1.050860167390	-0.438562759625
N	0.728548883397	-1.204145134903	1.629395097244
N	-0.825468027992	-1.886029887279	-0.312448240444
N	2.680326824839	0.170359754697	0.663303437966
H	-1.758252792680	4.393873183130	-1.331302534506
H	5.628400254823	1.670342146821	2.695963354458
H	-0.551186240833	3.110357148155	-3.080258482805
C	-1.264864846763	3.454584180264	-1.084578523248
C	4.719862668147	1.407973610368	0.743350778960
C	-0.585825365633	2.730589123176	-2.059811649619
C	4.798638431776	1.248847087727	2.129146302813
C	-1.296511246245	2.944510801724	0.215788739388
H	0.565260111855	1.867729917579	-4.425537337897
H	3.568477819552	0.969119637849	-1.043261383749
C	3.649368013705	0.857158670072	0.038194748438
C	0.983077183902	0.956443398478	-4.004268778800
C	0.042177644707	1.526307577378	-1.716088773427
C	3.789158262475	0.535575923747	2.768627308617
H	3.831938572388	0.397932452030	3.848487741603
C	-0.654004394157	1.740922666885	0.508001044638
C	1.715934417336	0.062139695090	-4.799949894931
C	0.786409288602	0.673511202090	-2.650995674965
H	-0.665291245532	1.325178095065	1.515932362482
C	2.735022876248	0.002505617979	2.015760429781
C	2.238957137069	-1.098716513286	-4.193922056893
H	2.816900760956	-1.809935119546	-4.783604739688
H	2.153652723656	-0.662738965856	4.650762494886
C	2.014849876387	-1.334614632154	-2.843266954625
C	1.612645679606	-0.762793754595	2.572185743916
C	1.428695137440	-1.032812614980	3.929664143306
C	2.513333098915	-2.500946206817	-2.096961223002
H	3.518758055683	-3.534536028002	-3.716679767290
C	0.311086781173	-1.774658047695	4.343917006820
C	3.263918949298	-3.540925688704	-2.657572430631
C	-0.368753162198	-1.924389771975	2.023109418079
H	-1.175832045105	-1.819466392588	-2.357297617477
C	2.595065919862	-3.514299299587	0.008912834406
C	-0.597188746921	-2.220157844005	3.361391701601
C	-1.549577153811	-2.194548102823	-1.404435453253
H	2.308564171837	-3.465484055433	1.059807861054
C	-1.242029270707	-2.327479870351	0.909542384024
C	3.683967117552	-4.590332917759	-1.840635842415
C	3.345171302672	-4.580371274660	-0.484490017189
H	-1.477917209214	-2.793406456439	3.649881348240
H	4.270580356134	-5.408512415470	-2.259996234594
C	-2.717927268117	-2.951152166837	-1.336576273392
H	3.655085568214	-5.381226348647	0.186003332788
C	-2.405577973446	-3.092590579173	1.048845797378
H	-3.272186874303	-3.175015648872	-2.247321098530
H	-2.727534802029	-3.440914000245	2.029170774872
C	-3.150733354068	-3.405893144301	-0.087507253313
H	-4.060503893928	-4.000155996643	0.002307070272
S	-0.050017852109	-2.193850388281	6.022382338559
C	1.326714572714	-1.443230801106	6.963910330123
H	1.328172635129	-0.351777866405	6.857928988658
H	2.289262588967	-1.876088617241	6.667877245538
S	2.040410484505	0.285231050811	-6.522975940996
C	1.225348643970	1.876148732942	-6.911786194779
H	0.144691498334	1.818219780801	-6.732831264938
H	1.681673303363	2.699002169168	-6.349685322991
H	1.121259205635	-1.703658001577	8.009327679615
H	1.406054859203	2.027209235704	-7.982821409454
Cl	5.954767894676	2.296377596381	-0.126506216665

Cl	-2.134888439334	3.802822962060	1.492815245577

7.2.11 $[Fe(5,5''\text{-diCl-4'-SMe-terpy})_2]^{2+}$ (S2)

Fe	1.006124181912	-0.828798383663	-0.223578568244
N	1.294609243926	-0.460326851539	-2.077520734651
N	2.162446303952	-2.525018988569	-0.791977347258
N	-0.024355707079	1.023579735745	-0.437538548477
N	0.724180244576	-1.200593172767	1.629981176352
N	-0.816998496975	-1.930269001769	-0.307662279711
N	2.692121234887	0.139295206035	0.645752557900
H	-1.741565658641	4.394937298486	-1.300697915738
H	5.605585846769	1.713789546711	2.670516538251
H	-0.514951354671	3.132653193765	-3.051331074496
C	-1.259948995838	3.447518768230	-1.061960272126
C	4.724107964451	1.390064981542	0.714925891698
C	-0.570264062332	2.735202703012	-2.038607846964
C	4.785933652669	1.270759709856	2.105393588932
C	-1.317379541604	2.916084406410	0.228717889605
H	0.582892970799	1.886528536624	-4.409705589381
H	3.596179545254	0.896134345296	-1.072113982178
C	3.664534715944	0.814332139971	0.012793362556
C	0.996924548341	0.970824831596	-3.993938221963
C	0.042561568377	1.520618068990	-1.706226858694
C	3.772410075279	0.570231667941	2.752846267532
H	3.800633224367	0.466533642701	3.836959798358
C	-0.688946196028	1.702496031608	0.510593991348
C	1.733500245806	0.081808600843	-4.793564008552
C	0.793275502916	0.676621050592	-2.644760567433
H	-0.720459522343	1.269401628894	1.510584627779
C	2.731234672731	0.009000154786	2.003291370326
C	2.248989446904	-1.086364891447	-4.195845749264
H	2.826652011474	-1.794666885909	-4.789249090975
H	2.154093633997	-0.636257890578	4.644712864690
C	2.018094490511	-1.332366475347	-2.847565920277
C	1.608815508402	-0.751114440522	2.568282495904
C	1.427763006562	-1.011345168851	3.927401588138
C	2.506746536032	-2.506670366896	-2.111361618052
H	3.540297408580	-3.524254877001	-3.727773218670
C	0.311495008693	-1.752682733907	4.348805735201
C	3.265896261463	-3.540439651215	-2.673894196996
C	-0.369394662793	-1.922607656331	2.029989612823
H	-1.156700455100	-1.900212165613	-2.361997831675
C	2.552720180730	-3.547039000112	-0.014162139906
C	-0.595599285278	-2.209925492172	3.371022264823
C	-1.530794021883	-2.247272069541	-1.399132098735
H	2.255585064700	-3.522148638925	1.033998189213
C	-1.240379930913	-2.339456131589	0.922324270524
C	3.675844603378	-4.604337066933	-1.875352783137
C	3.309936318572	-4.601072627093	-0.527230919839
H	-1.472444426273	-2.786477361114	3.664748514397
H	4.267227760468	-5.418428768294	-2.293774913903
C	-2.707183995627	-2.992224762901	-1.305071498104
C	-2.413757953108	-3.089328862331	1.069524045351
H	-2.750089792208	-3.415056187787	2.052565826216
C	-3.161293178198	-3.422802404323	-0.056174284545
H	-4.077854296560	-4.004337599634	0.037294573749
S	-0.045110588053	-2.160454470936	6.030017673580
C	1.330152594001	-1.398231032576	6.964461200132
H	1.326870740601	-0.307636790623	6.850704017068
H	2.293717502627	-1.829592150673	6.669731097709
S	2.067502170440	0.320166894313	-6.511757577465
C	1.263091088853	1.919274520163	-6.889577828183
H	0.181603783862	1.865734758743	-6.714719074542
H	1.722517939088	2.734169170964	-6.318626004560
H	1.126596709572	-1.652816039914	8.011709872790
H	1.448822851735	2.077844818472	-7.958644912204
Cl	5.965094849266	2.258000870521	-0.164910866794
Cl	-2.170671613993	3.757400706221	1.505808894070
Cl	-3.599162793746	-3.375669475479	-2.762331255335
Cl	3.791175315778	-5.908195454157	0.534262802013

7.3 Quintet

7.3.1 $[Fe(terpy)_2]^{2+}$(H0)

See the Supplementary Information of Ref. 2.

7.3.2 $[Fe(3\text{-Cl-terpy})_2]^{2+}$

Fe	0.913642037435	-0.934096691109	-0.256823615291
N	1.250367417532	-0.516854973671	-2.294761711184
N	2.024024922948	-2.651071762969	-1.029709850701
N	-0.136418578591	0.944644497299	-0.602555724165
N	0.607503838535	-1.242778311932	1.802693147367
N	-0.862752564208	-2.185043948143	-0.110755564737
N	2.604313886355	0.056300500069	0.693055975116
H	-1.738285542246	4.366111306536	-1.470407190185
H	5.377656992808	1.901730573284	0.137666763036
H	5.433586620221	1.832478665433	2.659581333393
H	-0.535363715776	3.064662031403	-3.217655388089
C	-1.294617577146	3.401112349358	-1.224742089888
C	4.613808726683	1.373514563962	0.707588234915
H	-1.908001426139	3.417908646730	0.862214959083
C	-0.620615515016	2.671711918849	-2.205323074480
C	4.641050690430	1.330448242219	2.103536063492
C	-1.389026719147	2.881976863852	0.068426381731
H	0.182753211063	1.659049952604	-4.657532580693
H	3.504726576209	0.743879687171	-1.046799537463
C	3.573833825995	0.725739488062	0.042042568789
C	0.665178167993	0.786253504553	-4.220737480177
C	-0.053440814633	1.437589397154	-1.867971117936
C	3.638921870879	0.638199130820	2.784786568096
H	3.645531759097	0.599154458756	3.873656213680
C	-0.791448775736	1.651149550316	0.336983801465
C	1.286734848520	-0.158216023337	-5.036046862626
C	0.646886015443	0.565893481041	-2.838964178089
H	-0.829747588666	1.212199900290	1.335056955160
C	2.630770892562	0.000544232906	2.052078646621
C	1.898883504195	-1.278615803043	-4.469838889840
H	2.357497949449	-2.025272942899	-5.110335833050
H	2.237092593615	-0.748673676885	4.729930650165
C	1.894076814027	-1.424912751620	-3.075195746818
C	1.545500318619	-0.793810280759	2.670659651840
C	1.486029210452	-1.114189177687	4.031554911840
C	2.501461702015	-2.540674520773	-2.304863767367
C	0.457866095326	-1.944903389454	4.474778081856
C	3.504482382289	-3.437371892655	-2.728943435590
C	-0.422349523858	-2.022892188089	2.229245182884
H	-1.101377025177	-2.295151889691	-2.167896726511

C	2.457749103618	-3.619485047196	-0.207811813920
C	-0.499721096795	-2.414381174797	3.573793173017
C	-1.574660114788	-2.495575998511	-1.205799740355
H	2.013297655601	-3.648898404834	0.787458839645
C	-1.356697271619	-2.412218262720	1.142682867850
C	3.955283486474	-4.447446483699	-1.874316750756
C	3.418577752899	-4.550179396759	-0.595059546426
H	-1.282959563699	-3.081603633970	3.918644802895
H	4.729673797870	-5.135629781546	-2.213078626553
C	-2.847585372313	-3.053950437330	-1.133238890664
H	3.743450823145	-5.330666449257	0.091902606813
C	-2.659488209602	-2.942020647302	1.265128938061
H	-3.395287926261	-3.306238645282	-2.040147998580
C	-3.398640385602	-3.271853872675	0.125824743485
H	-4.401427859960	-3.683750637407	0.234164391315
H	0.406983250743	-2.241423735800	5.522461346927
H	1.283860473979	-0.032266400632	-6.119175742173
Cl	4.298161062032	-3.336147259477	-4.295623184963
Cl	-3.473909350076	-3.180078553755	2.806755657734

7.3.3 [Fe(4-Cl-terpy)$_2$]$^{2+}$

Fe	1.004713090893	-0.855211934859	-0.229023885041
N	1.300523339161	-0.456863493384	-2.270219248969
N	2.239391499780	-2.514315715583	-0.945762201419
N	-0.096550202953	1.005940936157	-0.610539578531
N	0.692753408484	-1.237302408928	1.812194422742
N	-0.897907082233	-1.946452013605	-0.140328624559
N	2.726064032569	0.049804730740	0.776915501812
H	5.542373048098	1.857759832058	0.320111971575
H	-0.644438897422	3.017579382901	-3.292518914505
C	-1.367728894332	3.376731516642	-1.289264493961
C	4.769417894595	1.312330267433	0.860941973823
H	-1.939762266103	3.461944759656	0.811406907658
C	-0.682570136144	2.656950032751	-2.266291526546
C	4.815603584915	1.161736840040	2.249151404655
C	-1.413169140439	2.918688361098	0.028908505305
H	0.474288127288	1.812104810436	-4.642797292441
H	3.637390232622	0.820734666123	-0.921858513676
C	3.707572466867	0.737217993695	0.163889376730
C	0.929416532288	0.934707248290	-4.186421204611
C	-0.046659452870	1.468640845919	-1.889371052982
C	3.798437205224	0.455863861293	2.892599685216
H	3.816535845990	0.334339239484	3.975139467774
C	-0.762199752764	1.721513349401	0.314464413141
C	1.725918580132	0.080606366358	-4.951770258861
C	0.738213591817	0.640313829466	-2.831447908985
H	-0.780971102540	1.315900768673	1.327031441787
C	2.757801486186	-0.088905743611	2.130984463822
C	2.307529134071	-1.044376412721	-4.363369512871
H	2.930219560247	-1.711500428469	-4.957550672831
H	2.179391020267	-0.741881436579	4.811680922444
C	2.069403906952	-1.294981360376	-3.006441461214
C	1.612990466271	-0.822161549041	2.715777155500
C	1.439012680699	-1.068968927601	4.083058080040
C	2.590334789491	-2.462795110399	-2.260393993724
H	3.652013945588	-3.415624067122	-3.894009079016
C	0.288384066901	-1.739584266134	4.504326055284
C	3.370861226456	-3.468511964228	-2.843172510292
C	-0.422286435867	-1.895030377886	2.211887070462
H	-1.270861613356	-2.004249449966	-2.183624767958
C	2.637663146731	-3.561029238037	-0.199573798613
C	-0.658245543087	-2.156539799574	3.566581519509
C	-1.635491283768	-2.315137650812	-1.203997902428
H	2.330946169837	-3.554752449093	0.847257354638
C	-1.312299629096	-2.307954496540	1.104536203779
C	3.784712751827	-4.547552325865	-2.061335928897
C	3.408260992545	-4.601575781093	-0.717126062634
H	-1.557476619972	-2.676813897225	3.892442355233
C	-2.814007616268	-3.048780492260	-1.096819010187
H	3.708890472263	-5.427403919164	-0.073168454290
C	-2.489253124709	-3.038087397772	1.303917795093
H	-3.377894522010	-3.321902176049	-1.986761112531
H	-2.816634670360	-3.319085496057	2.302762896897
C	-3.236898253429	-3.402754403207	0.186051798368
H	0.129254925414	-1.937280127782	5.564549138153
H	1.894676616370	0.292564761844	-6.007636070183
Cl	-4.717427700656	-4.313739270335	0.396752605952
Cl	-2.177160372682	4.867777514430	-1.723588192449
H	4.393984770867	-5.338785230764	-2.499844818340
H	5.634207703351	1.591313897230	2.827625566151

7.3.4 [Fe(5-Cl-terpy)$_2$]$^{2+}$ (H1)

Fe	0.938521994235	-0.848980737705	-0.239450868708
N	1.198089812037	-0.503026883662	-2.296452155520
N	2.057943443942	-2.595697742671	-0.978745587570
N	-0.087514996995	1.044780922206	-0.617412445215
N	0.679930817102	-1.278400181864	1.800386723816
N	-0.867151699617	-2.101410607278	-0.141591262627
N	2.614413903966	0.145351052841	0.753536558710
H	-1.797472537043	4.390575900802	-1.569189437105
H	5.411527357583	1.971614106720	0.257187882144
H	5.489915937430	1.783621912791	2.772043266368
H	-0.566327333613	3.078485105067	-3.287540889965
C	-1.321347383285	3.447750136942	-1.299208617922
C	4.644783583258	1.428990975840	0.809447385550
H	-1.927982692976	3.501658689083	0.789145216981
C	-0.629354295266	2.713172961851	-2.263248396934
C	4.683304415036	1.321850803969	2.201552119116
C	-1.396332925789	2.960316327194	0.007512832264
H	0.503121424540	1.828177166249	-4.647788537078
H	3.514682963010	0.891724226071	-0.962026841637
C	3.589883876375	0.826389783682	0.124652862875
C	0.889671884704	0.912229582204	-4.204270767355
C	-0.021314934377	1.507358037916	-1.895929109243
C	3.676936083110	0.613564623507	2.859998506863
H	3.695338520026	0.517929381116	3.945178785100
C	-0.761973473857	1.754777192125	0.305332558323
C	1.575222073983	-0.014783512773	-4.993482456898
C	0.709168984677	0.635419829497	-2.844737813182
H	-0.785580422774	1.341430013633	1.314796448090
C	2.650884480993	0.028636364168	2.109152641648
C	2.069669277375	-1.187559772380	-4.421720759837
H	2.601653129918	-1.912920946370	-5.035239874538
H	2.091544988592	-0.599371593200	4.800142953487
C	1.869411588584	-1.405267409744	-3.052035436881
C	1.552002697890	-0.767044843151	2.702877883425
C	1.396242253969	-1.013101345861	4.071429278956
C	2.364659992906	-2.578898626341	-2.305584920399
H	3.359034192007	-3.593911743208	-3.948204037493

C	0.327363654214	-1.808575467109	4.493902019558
C	3.119847712194	-3.606802751797	-2.885834276299
C	-0.362063779364	-2.046622988989	2.200904559977
H	-1.287604257739	-2.101141305847	-2.181649521238
C	2.493610065396	-3.605683348067	-0.209387613350
C	-0.561889619320	-2.336122873707	3.557421893963
C	-1.622794540866	-2.436329165055	-1.199693726726
H	2.220761299473	-3.585797968643	0.846030466136
C	-1.239353792483	-2.502932821005	1.105542076687
C	3.576290459287	-4.658434106434	-2.095767769274
C	3.258490074080	-4.647704687594	-0.736191442074
H	-1.395040455965	-2.957091059879	3.882586926460
H	4.166420273757	-5.465898710602	-2.528593934549
C	-2.787976863785	-3.190902763154	-1.055256259215
C	-2.392382813799	-3.274167781996	1.302607446184
H	-2.682012195642	-3.600556949407	2.299992722168
C	-3.182418676581	-3.625791550797	0.211728287624
H	-4.084422153849	-4.222052484985	0.345711173357
Cl	3.797715631458	-5.936467717067	0.320997456949
Cl	-3.732806117507	-3.592130748140	-2.474666790127
H	1.723817699476	0.178326917235	-6.056069467392
H	0.189147675908	-2.017308426229	5.554982683571

7.3.5 [Fe(5,5''-diCl-terpy)$_2$]$^{2+}$ (**H2**)

Fe	0.925500577801	-0.899261406976	-0.251712503292
N	1.223366484792	-0.502264704832	-2.288137527311
N	2.070663329700	-2.622374014082	-0.996896329211
N	-0.081699950439	1.021205561050	-0.602029845108
N	0.657129228169	-1.263845737983	1.793753732249
N	-0.894420718584	-2.125213038485	-0.135035227607
N	2.595418810919	0.141688882853	0.727251754208
H	-1.821155809255	4.361918373337	-1.537417162653
H	5.487812951892	1.784546836752	2.727822408834
H	-0.551671675778	3.087478260081	-3.248861720901
C	-1.335664633615	3.422015372136	-1.277537931865
C	4.627644012189	1.396656518215	0.774805720806
C	-0.620897228526	2.703154175207	-2.232153235812
C	4.675897365203	1.321022453810	2.167905947930
C	-1.414010044379	2.904801650746	0.016775692133
H	0.557004943123	1.866892778192	-4.611812142988
H	3.506152350412	0.859976395615	-1.003659460465
C	3.571326024668	0.800780342616	0.083401684114
C	0.937616881850	0.943700021890	-4.178314284342
C	-0.004035693023	1.497442908057	-1.875127437846
C	3.658917807234	0.634044103862	2.825829710898
H	3.679221158317	0.560660749031	3.912526880126
C	-0.771151874936	1.704347485163	0.324618292133
C	1.633366129041	0.030909885207	-4.972760318706
C	0.739801625249	0.644578880728	-2.824561687617
H	-0.804782490980	1.289816537445	1.332720532852
C	2.627132910814	0.043939505557	2.084765859363
C	2.122860347067	-1.151103839847	-4.412896253319
H	2.664011921274	-1.865389509745	-5.031319762907
H	2.063379100806	-0.530498003565	4.784504817791
C	1.906334552395	-1.390617125836	-3.050630284161
C	1.528046014830	-0.736497758811	2.688280114763
C	1.369880623717	-0.957971642843	4.062092180188
C	2.394817221099	-2.578020984185	-2.318224458310
H	3.415907081753	-3.560259411919	-3.963992343528
C	0.301944960400	-1.745475079502	4.496561053930
C	3.159516910981	-3.593554613118	-2.906229177467
C	-0.385773724059	-2.024230905683	2.207753867208
H	-1.314511633318	-2.155838592694	-2.174625157475
C	2.494943030090	-3.644556288462	-0.238582756352
C	-0.586811353766	-2.289941908764	3.567554530310
C	-1.649135926048	-2.476183306978	-1.187639158245
H	2.208481591696	-3.642217407230	0.813317392842
C	-1.264451174379	-2.501362158716	1.119757931493
C	3.603331673712	-4.660631735899	-2.129097637924
C	3.265976767778	-4.677973291551	-0.774727046425
H	-1.420503347576	-2.904524483233	3.903097800278
H	4.199926464224	-5.459360955881	-2.569288442089
C	-2.814504316382	-3.228849346529	-1.029299331524
C	-2.418066668045	-3.266821073786	1.331090858195
H	-2.709310424861	-3.573612894204	2.334116841412
C	-3.207388303180	-3.638855859352	0.245941487940
H	-4.109614166589	-4.232015205290	0.391858407499
Cl	3.788099915226	-5.986702915133	0.264009859610
Cl	-3.759312383034	-3.654994779795	-2.439677147294
H	1.795385670145	0.240875564336	-6.030034250132
H	0.161918225383	-1.935443489099	5.560892511389
Cl	-2.299413558376	3.749608979734	1.268694211456
Cl	5.871900435180	2.241491248390	-0.121718059074

7.3.6 [Fe(6-Cl-terpy)$_2$]$^{2+}$

Fe	0.868852207659	-1.110960486384	-0.297552559505
N	1.361109500465	-0.482540263993	-2.280532504196
N	2.138274593632	-2.744882619148	-1.165678181789
N	-0.197233794388	0.782456923824	-0.594704572372
N	0.525307252145	-1.161629160933	1.811851224707
N	-1.028611359893	-2.269422116626	-0.010039881877
N	2.600429453922	-0.105975533849	0.607678536595
H	-1.868627983939	4.218972353034	-1.319754480137
H	5.508177338056	1.482919512899	-0.065221916838
H	5.478414299167	1.759341949414	2.445817433209
H	-0.407787808224	3.144119656756	-3.024397838956
C	-1.403846384751	3.254636618240	-1.114560090853
C	4.693562466340	1.085849949738	0.540019381176
H	-2.247566368006	3.030584705032	0.878130270033
C	-0.584620876705	2.651765831109	-2.068948641656
C	4.674175444404	1.235211556754	1.928456934746
C	-1.614526901434	2.601819471328	0.101716399060
H	0.771010522800	2.076673898133	-4.410357376653
H	3.622139850011	0.284202462060	-1.158202344590
C	3.639005629807	0.412559773191	-0.075546863805
C	1.166262629611	1.134587595782	-4.035255871631
C	0.004171904421	1.413928140411	-1.783949343206
C	3.604785315853	0.702451576832	2.647755301999
H	3.568969776909	0.808222942488	3.731466377703
C	-0.991057747330	1.373944240219	0.318598829607
C	1.983454554626	0.346560935874	-4.842955696284
C	0.866920908709	0.689221471981	-2.739166033675
H	-1.129694894587	0.842732929390	1.260777848161
C	2.583172474500	0.033161701808	1.962210599671
C	2.483010617924	-0.865608536775	-4.359847629823
H	3.118154174666	-1.484479702053	-4.991097806437
H	1.959662195245	-0.144755663058	4.700265415463
C	2.148510698467	-1.257664243936	-3.058658349240
C	1.424045658024	-0.582530328699	2.638898412871
C	1.237656566821	-0.606721445025	4.029375217731

C	2.603911968151	-2.520231556977	-2.430692483667
H	3.809338114914	-3.235327056249	-4.080195749078
C	0.107900917641	-1.238862846373	4.544280747980
C	3.445360070966	-3.429751779801	-3.073628046552
C	-0.570458018947	-1.782721241826	2.302634255619
C	2.513009352286	-3.869973774120	-0.556051641299
C	-0.810634822454	-1.839503746536	3.679679078557
C	-1.788141091302	-2.799653528702	-0.969561838555
C	-1.459121045124	-2.384826341630	1.281253843999
C	3.818389248574	-4.600509361073	-2.409765212504
C	3.347185523517	-4.836721923918	-1.121532612953
H	-1.691359915236	-2.339422126957	4.078365850457
H	4.473869835740	-5.324668828168	-2.894087229659
C	-2.992778885343	-3.469497137947	-0.745694897831
H	3.610464290339	-5.735149922442	-0.566625741981
C	-2.653822969473	-3.033521021021	1.599165444588
H	-3.560864725126	-3.881731789887	-1.577152059707
H	-2.985632143763	-3.113962540942	2.632549212415
C	-3.426164061261	-3.581883277315	0.572647858189
H	-4.362069742469	-4.092171144051	0.799605528186
H	-0.057084129123	-1.271152248707	5.621698335497
H	2.230384843041	0.672257041170	-5.853250556083
Cl	1.903856314361	-4.139484378314	1.071650426950
Cl	-1.215666844841	-2.633399564032	-2.624116711777

7.3.7 $[Fe(4'\text{-}Cl\text{-}terpy)_2]^{2+}$

Fe	1.011569359380	-0.808578486924	-0.217886257243
N	1.302057438114	-0.379366110246	-2.252144762900
N	2.135409961679	-2.522485902642	-1.002207344395
N	0.028454124262	1.126966026155	-0.534836112051
N	0.740073696461	-1.216404068999	1.821953271363
N	-0.813082948058	-2.021137338931	-0.121634456691
N	2.685842403734	0.192427622265	0.786921848231
H	-1.660893587720	4.511581027201	-1.393454069548
H	5.517541468544	1.969553068408	0.303054067734
H	5.592425931346	1.767984547698	2.817183504623
H	-0.433945268815	3.243434532341	-3.146112049131
C	-1.191507109505	3.558205880589	-1.150482486993
C	4.740851394044	1.438067247421	0.852362881010
H	-1.801794615197	3.553675685273	0.937372141983
C	-0.501594252914	2.847752731230	-2.133353417525
C	4.777755076031	1.323943319281	2.244224244031
C	-1.271926224662	3.032157549741	0.141110916419
H	0.621008268366	2.026484352185	-4.539753170075
H	3.600365355083	0.936233945161	-0.921978460714
C	3.674550530355	0.860313642784	0.164027076879
C	0.999563490949	1.097636733327	-4.118758692178
C	0.097381139959	1.627779103166	-1.798994522995
C	3.758196101227	0.631053427715	2.897861564415
H	3.774729720639	0.529764946136	3.982625641628
C	-0.643685667831	1.815854107327	0.405757018592
C	1.678472193535	0.179570483995	-4.921243698610
C	0.822342785941	0.776979283890	-2.768909841470
H	-0.669401579239	1.376143810585	1.404010878012
C	2.722855537755	0.068803404852	2.142268109578
C	2.170765527942	-1.016404193753	-4.396008908888
H	2.696230731889	-1.724711451250	-5.033055981521
H	2.148068913110	-0.546672481520	4.831518997925
C	1.966471540103	-1.262815669037	-3.034699623975
C	1.612398572554	-0.713986585594	2.728167185971

C	1.455322471524	-0.952697616109	4.097311587092
C	2.455476105508	-2.466170646971	-2.324272919918
H	3.460992370262	-3.420832451152	-3.993170109914
C	0.374805147608	-1.738690824306	4.500801790649
C	3.211783683944	-3.473627621410	-2.934175243613
C	-0.309890065599	-1.973759389291	2.220661046291
H	-1.228263741327	-2.009775987881	-2.154777027791
C	2.564506462680	-3.565061561402	-0.267919208854
C	-0.523749165709	-2.265573227447	3.571528684414
C	-1.575570786344	-2.353421022852	-1.179537468588
H	2.278786240577	-3.566840809753	0.784830078490
C	-1.193203721452	-2.424276029668	1.121827994396
C	3.650559460514	-4.552740729878	-2.165969539469
C	3.327102294858	-4.600381103472	-0.808009232784
H	-1.363208409203	-2.875913317882	3.897947482137
H	4.241177848322	-5.346406464933	-2.624958009518
C	-2.745083095469	-3.102798700621	-1.056280304739
H	3.650959532836	-5.424680476161	-0.173417538176
C	-2.349507289929	-3.187795358779	1.318362770612
H	-3.328797211609	-3.347774604674	-1.942874854483
H	-2.640775340548	-3.511476441190	2.316372341794
C	-3.132687736140	-3.530866751964	0.215764348417
H	-4.037041388990	-4.124724257366	0.351237469103
Cl	1.916582671863	0.533893101824	-6.619073129881
Cl	0.141261902765	-2.068013546488	6.204447852845

7.3.8 $[Fe(3'\text{-}Cl\text{-}terpy)_2]^{2+}$

Fe	1.000690246443	-0.771852804615	-0.212248358235
N	1.250107165313	-0.414787930988	-2.286306220005
N	2.148001420724	-2.481141694273	-0.918046950562
N	0.025832636873	1.124085103941	-0.583413815331
N	0.737559786233	-1.234972254182	1.838515841495
N	-0.822145353589	-1.960030840517	-0.157427745127
N	2.639779380713	0.211119531106	0.805028559935
H	-2.383543846460	3.995597993448	-1.552161006449
H	5.649915195449	1.601760767717	0.156064869121
H	5.961161356756	1.063104025524	2.603155718937
H	-1.027998586417	2.866728342791	-3.288330979298
C	-1.698138368478	3.195396986854	-1.270672702192
C	4.860604729001	1.129718435715	0.740275154331
H	-2.155892011521	3.264792599227	0.855288404193
C	-0.929566858505	2.564131810072	-2.250540727406
C	5.023230171331	0.838647960146	2.094310337256
C	-1.583733322904	2.790416726550	0.058985245977
Cl	0.830191077217	2.624634697894	-4.896645576229
H	3.480612644155	0.988388969158	-0.924800391228
C	3.652305115286	0.789505115941	0.134111308240
C	1.182447138805	1.083528967049	-4.134777115187
C	-0.052407059110	1.534924029585	-1.882730252301
C	3.973766791643	0.248063052203	2.801653536925
H	4.102333403383	0.003590138052	3.851748379385
C	-0.713203622145	1.742668915741	0.354920328987
C	1.924256411274	0.172342847452	-4.892886212987
C	0.799249648201	0.754443580649	-2.816539803053
H	-0.595859225591	1.383692614264	1.378587742593
C	2.773777606185	-0.042957984332	2.139195722148
C	2.326306833751	-1.032773555872	-4.330608535417
H	2.907755661750	-1.737414395758	-4.922552728880
Cl	2.166721045894	-0.018608519868	5.402329074253
C	1.997455712441	-1.291265061667	-2.995509399439

C	1.589463241519	-0.696000375286	2.752194447033
C	1.258766332145	-0.800787957643	4.120183399636
C	2.453095989358	-2.480153033784	-2.243996896528
H	3.409141792046	-3.525192733525	-3.887966402868
C	0.125859301305	-1.522462790447	4.509547121068
C	3.175863623859	-3.529965037703	-2.824247005750
C	-0.375658539301	-1.914148461800	2.198194198504
H	-1.192940447297	-1.947282275357	-2.199299862556
C	2.559622332402	-3.506506925610	-0.149299472903
C	-0.694106656774	-2.099637278443	3.547855640347
C	-1.550778280755	-2.308188499822	-1.234367454320
H	2.285421621107	-3.461915940411	0.905496905650
C	-1.214470215580	-2.382791062284	1.074933778609
C	3.598249600404	-4.589664829559	-2.021008355748
C	3.290689256150	-4.579078935058	-0.658359035031
H	-1.577929527923	-2.655339757067	3.854923895042
H	4.161896556511	-5.415257079110	-2.457037980258
C	-2.698915997680	-3.093398920614	-1.140882951296
H	3.603277545115	-5.385993314090	0.003520920064
C	-2.350263601072	-3.184464054651	1.241092638710
H	-3.256461260098	-3.348888477344	-2.041157145373
H	-2.649196434403	-3.529228970452	2.229483455735
C	-3.099680112067	-3.541933586641	0.119973787270
H	-3.986997452764	-4.165733799577	0.232143538747
H	-0.120675787100	-1.609876899091	5.567465414449
H	2.204457196793	0.423335826360	-5.915131282681

7.3.9 [Fe(4'-SMe-terpy)$_2$]$^{2+}$(**S0**)

See the Supplementary Information of Ref. 2.

7.3.10 [Fe(5-Cl-4'-SMe-terpy)$_2$]$^{2+}$ (**S1**)

Fe	1.017513066382	-0.789210894805	-0.213202534115
N	1.311621227800	-0.413455201290	-2.284111279348
N	2.174578005868	-2.490376743577	-0.968682828018
N	-0.014186031530	1.092820393234	-0.624051173180
N	0.713168720258	-1.246816809303	1.838427772379
N	-0.825951988678	-1.963571008153	-0.134749617257
N	2.700717062854	0.116856649772	0.851523492391
H	-1.696605431190	4.466756823134	-1.543529331582
H	5.515001126297	1.935585366126	0.425158551387
H	5.580102596654	1.674719387631	2.934427396028
H	-0.479099956754	3.157558117474	-3.274342366938
C	-1.226145428584	3.518856771634	-1.281987893211
C	4.740190797749	1.385406423983	0.958581521502
H	-1.820680166203	3.560570414755	0.809464207120
C	-0.542032980098	2.784565254975	-2.252737471105
C	4.772068172184	1.238210846466	2.346519906778
C	-1.297009852262	3.021585322226	0.020701163215
H	0.588821370781	1.917445740578	-4.616689337552
H	3.624113305046	0.897898923041	-0.835933544488
C	3.686236464729	0.807576671611	0.249928842858
C	0.999691264861	1.004924126500	-4.191895564905
C	0.058017691449	1.573333686784	-1.894476133163
C	3.752450159045	0.522398928091	2.977500999076
H	3.761165145632	0.398854689073	4.059921916702
C	-0.678639949040	1.803876508755	0.305076790434
C	1.730089538337	0.102035234455	-4.987426739988
C	0.809660737252	0.711558324078	-2.841357654990
H	-0.713286829072	1.378440560895	1.309460488917
C	2.726618560387	-0.028187868503	2.203309196893
C	2.245970405473	-1.067062282172	-4.390975719757
H	2.813819573559	-1.778845534300	-4.989146052628
H	2.139759900196	-0.703741812524	4.849519744910
C	2.018600098945	-1.292912352876	-3.036254679137
C	1.592464785684	-0.804694391509	2.765461345977
C	1.418690131158	-1.070630481923	4.123305716140
C	2.509454254880	-2.471178805882	-2.288319629681
H	3.526620460013	-3.479459055097	-3.920230068793
C	0.298340109387	-1.820013686134	4.531796565643
C	3.265047914470	-3.500151109395	-2.863177883128
C	-0.371925567118	-1.968277835109	2.215021379900
H	-1.190241550548	-1.937894746380	-2.185835413367
C	2.578807001766	-3.517173656582	-0.202955901058
C	-0.609185836678	-2.273791016986	3.552863479218
C	-1.556068403344	-2.280406275423	-1.217393620837
H	2.288729814884	-3.502998330010	0.848052249891
C	-1.245227925189	-2.375463135837	1.093892465852
C	3.690748218760	-4.567414312720	-2.075586960181
C	3.337937786687	-4.565665856480	-0.725072369653
H	-1.482787112611	-2.855006529517	3.846010172777
H	4.280470425973	-5.377784532328	-2.503130605749
C	-2.735492696671	-3.019279312201	-1.115156139907
C	-2.418983212530	-3.122217356566	1.251014348184
H	-2.746143735325	-3.448148266747	2.237096168224
C	-3.182653508834	-3.452895654106	0.134270723932
H	-4.100299853829	-4.031103143718	0.235454458923
S	-0.058718467420	-2.240234888654	6.205379981074
C	1.308976221253	-1.484399472437	7.155860535021
H	1.303724158579	-0.392926782197	7.052447078686
H	2.274887675238	-1.911417607452	6.863154946423
S	2.052977805763	0.328024539745	-6.704792037511
C	1.250842544397	1.924506144309	-7.097651489309
H	0.169764664594	1.874245950744	-6.920449701030
H	1.713156318573	2.743858835466	-6.535809477657
H	1.098117807115	-1.749868575269	8.198896825544
H	1.435710346565	2.070789238334	-8.168618163942
Cl	3.833786025077	-5.873566462817	0.331894383927
Cl	-3.640161979042	-3.394852086887	-2.569079432761

7.3.11 [Fe(5,5"-diCl-4'-SMe-terpy)$_2$]$^{2+}$ (**S2**)

Fe	0.987258194577	-0.864797753636	-0.233946384330
N	1.333918390705	-0.424502817536	-2.279684602609
N	2.186085417999	-2.533753823448	-1.003751909604
N	-0.021725024737	1.037086733290	-0.606570733355
N	0.682073705587	-1.248800444121	1.829108785450
N	-0.875951566796	-2.015597191425	-0.115790356703
N	2.666762119463	0.085023064173	0.797745075319
H	-1.730012841792	4.417105953985	-1.484720501735
H	5.572631743815	1.673786287000	2.835953490294
H	-0.465766823941	3.160835580971	-3.214984863938
C	-1.252185181277	3.469254481140	-1.240824200701
C	4.707755179077	1.329055534856	0.876029501432
C	-0.540871558057	2.758854593392	-2.205442685092
C	4.757422138785	1.228030782213	2.266818319354
C	-1.331641043640	2.929068808596	0.043337701440
H	0.642262121650	1.960863629198	-4.568390559072
H	3.595325801462	0.822844238972	-0.915782818204
C	3.650589708527	0.750134932126	0.171105449845
C	1.050152272902	1.040277551047	-4.158420724066
C	0.066033144339	1.544449793128	-1.866813145659

C	3.730869030399	0.538100838420	2.908302055279
H	3.746217131487	0.447207261031	3.993724595409
C	-0.705638403212	1.714251376466	0.329452016666
C	1.799240119098	0.161901078200	-4.961482228846
C	0.836615444890	0.712532367194	-2.817859041369
H	-0.754064740276	1.279473912964	1.328584562269
C	2.696544183327	-0.026823454854	2.154084883302
C	2.311474505933	-1.020567905390	-4.383583192211
H	2.894857705810	-1.713824763502	-4.988443697231
H	2.122726236726	-0.627591865181	4.819615074641
C	2.060203318714	-1.280659674300	-3.041297082859
C	1.566700824462	-0.782381427674	2.739640720980
C	1.398354910698	-1.012877387210	4.106424050187
C	2.542012659992	-2.478981986849	-2.315369170362
H	3.588937244311	-3.444837458853	-3.953752290688
C	0.280798733761	-1.748951000529	4.539167653521
C	3.304730028600	-3.493926976423	-2.903639023862
C	-0.401955314330	-1.959144518421	2.231163035856
H	-1.249214855893	-2.033014610090	-2.164953963470
C	2.569486229196	-3.580649638072	-0.257001123004
C	-0.633454608820	-2.228463916623	3.575134819331
C	-1.606033357543	-2.360274268256	-1.187876176296
H	2.265687761538	-3.589077895978	0.790147981828
C	-1.282838740426	-2.398662108902	1.124159423444
C	3.708049821172	-4.585651199678	-2.137180062171
C	3.329089026013	-4.621982255718	-0.794976067358
H	-1.506694498341	-2.799516114548	3.888341109744
H	4.302045471003	-5.386724905295	-2.576301388990
C	-2.779208532143	-3.107884228000	-1.062481659922
C	-2.450444853441	-3.147632099319	1.305958640857
H	-2.771960270729	-3.451152518096	2.300875899087
C	-3.215526617327	-3.511660786497	0.199703022387
H	-4.128634688468	-4.093451769853	0.320347847635
S	-0.071470365013	-2.125680707860	6.222134578121
C	1.299265269150	-1.346439029153	7.148475599935
H	1.291756472066	-0.257827538896	7.018703982509
H	2.264101846452	-1.779582278463	6.861013503233
S	2.153728225734	0.429409588583	-6.664986478735
C	1.352265513252	2.031375003931	-7.035284275800
H	0.268297831109	1.972245906849	-6.879563844882
H	1.801029617581	2.838702606993	-6.445683080751
H	1.093518082291	-1.587643408098	8.198434157250
H	1.556439593082	2.203321112886	-8.098801341526
Cl	3.795177578011	-5.961437760977	0.232997848144
Cl	-3.687168093357	-3.524330751118	-2.500899122213
Cl	-2.209045325787	3.759190571183	1.312245729578
Cl	5.959990950600	2.179689650048	-0.006173316715

8 Optimized B3LYP*/ TZVP level geometries of the studied complexes

8.1 Singlet state

8.1.1 $[Fe(terpy)_2]^{2+}$(**H0**)

See the Supplementary Information of Ref. 2.

8.1.2 $[Fe(3\text{-}Cl\text{-}terpy)_2]^{2+}$

Fe	1.004476206157	-0.847488160043	-0.226592082029
N	1.258638074351	-0.482111002689	-2.079988327389
N	2.093753240811	-2.448588077927	-0.738637814744
N	-0.013370818634	0.884292942429	-0.340483786513
N	0.726674899518	-1.200735375198	1.625848114019
N	-0.685567835648	-1.920396020874	-0.334546963031
N	2.627892457616	0.087213667167	0.503228682493
H	-1.832291935689	4.231298348056	-1.019340548561
H	5.421458027724	1.835455957344	-0.156037398577
H	5.605718371898	1.732989033250	2.345815816014
H	-0.628179564929	3.063345779496	-2.849920865939
C	-1.322521065584	3.294286039124	-0.827653952273
C	4.673238060510	1.331386301078	0.443755646358
H	-1.838818026445	3.199539302678	1.271217773081
C	-0.649633207630	2.640567184369	-1.853044656216
C	4.771308330259	1.272584702400	1.829030752780
C	-1.329027114216	2.726489114954	0.440762460700
H	0.490716627564	1.889995428277	-4.340104438920
H	3.485959084468	0.755657195168	-1.257842695705
C	3.588304988949	0.727093120024	-0.179765387144
C	0.917102537109	0.985018306992	-3.926192578638
C	-0.003599099105	1.437495872517	-1.585502339837
C	3.782319666468	0.609839080266	2.546416532008
H	3.838492915843	0.545522676708	3.626280425538
C	-0.663102190470	1.524445109393	0.642081441829
C	1.669466886147	0.129589501561	-4.719545208721
C	0.734793802283	0.653635450509	-2.585605197778
H	-0.650691812477	1.060290820301	1.620497900145
C	2.719340770921	0.027390969604	1.861511156233
C	2.199061899513	-1.043290774585	-4.186366597762
H	2.781313836859	-1.696729905451	-4.814798670033
H	2.128131299517	-0.449959932076	4.592636724405
C	1.961446837143	-1.351588815608	-2.846247511615
C	1.612214818170	-0.693035159449	2.508743465244
C	1.424190295483	-0.858712668427	3.878894029182
C	2.396671250267	-2.536446129676	-2.072395359980
C	0.300349114663	-1.551279347032	4.309337332371
C	3.049923215294	-3.687696831292	-2.537154131947
C	-0.368934095228	-1.893362959764	2.023104695773
H	-0.861602551459	-1.871028908525	-2.390374862997
C	2.471698409644	-3.409955337221	0.109964700698
C	-0.604042650790	-2.072966420752	3.387331171655
C	-1.301627915752	-2.244701940163	-1.475775609283
H	2.209741891896	-3.271944354612	1.150627299512
C	-1.176219343505	-2.352698274116	0.869574486481
C	3.439022823126	-4.687295054923	-1.650659805851
C	3.158295026540	-4.541635159140	-0.304402637156
H	-1.472603491424	-2.602702552605	3.740283501084
H	3.949550482639	-5.567008677180	-2.023225285207
C	-2.449152661653	-3.021846932335	-1.509693740566
H	3.452380304761	-5.295686111500	0.414981653753
C	-2.330210530490	-3.150372292899	0.875120088337
H	-2.916741779794	-3.264014041866	-2.455941333307
C	-2.969277580574	-3.484323512560	-0.315199893161
H	-3.859387632188	-4.101306170834	-0.294133009352
H	0.118835668856	-1.688981882562	5.369116567904
H	1.850564198474	0.372661963775	-5.760054305924
Cl	3.398724223911	-3.983038232126	-4.231212757788
Cl	-3.048485641665	-3.802386851425	2.337295336347

8.1.3 $[Fe(4\text{-}Cl\text{-}terpy)_2]^{2+}$

Fe	1.023828857674	-0.843377772752	-0.221348164865
N	1.287289062194	-0.481695479796	-2.077968425702
N	2.117486118538	-2.462062789014	-0.718378415578
N	0.010569974983	0.898973128309	-0.372773882558
N	0.734470847717	-1.198906679492	1.632170147267
N	-0.695771333482	-1.888963186017	-0.348760733181
N	2.648696665809	0.071266648846	0.546254109898
H	5.452229431834	1.817487857701	-0.076613668485
H	-0.712785123374	2.971699231965	-2.949552799564
C	-1.372269834719	3.238204104188	-0.917974643026
C	4.700511184483	1.306766208418	0.513151777174
H	-1.856876444648	3.221816724466	1.192885320754
C	-0.700262062892	2.582840990637	-1.939718967556
C	4.795231813118	1.224454298249	1.897417970255
C	-1.345729546293	2.731949508330	0.374232608107
H	0.462174856566	1.787608231010	-4.419913773785
H	3.513778587436	0.765054100552	-1.200538386662
C	3.614200085127	0.717774056142	-0.122836962154
C	0.905343435373	0.905946400403	-3.974123064586
C	-0.012546908881	1.413645110612	-1.634580683206
C	3.801381910253	0.554427591229	2.601616578060
H	3.853429243578	0.471811353692	3.680608455526
C	-0.640139545074	1.557904269297	0.596146379005
C	1.658469783593	0.027396977983	-4.749165609309
C	0.737083154182	0.623611724809	-2.621316527519
H	-0.601502839994	1.132954561819	1.591529283219
C	2.737395581728	-0.011206857464	1.905236725920
C	2.212888130245	-1.113646821643	-4.175715444586
H	2.790485554327	-1.803216671088	-4.778354311785
H	2.122062821908	-0.579483747927	4.631069251978
C	2.002820416369	-1.351816728346	-2.819358280248
C	1.622552543238	-0.734847205667	2.535524893782
C	1.419225375818	-0.952913051813	3.896865254661
C	2.474408683961	-2.501952587436	-2.034645929426
H	3.482317094036	-3.569179172954	-3.607542642499
C	0.286937943070	-1.657241837827	4.299562231734
C	3.212512551643	-3.558249710581	-2.558775320566
C	-0.366679873519	-1.881172849060	2.007574641657
H	-0.963772629995	-1.888921732431	-2.401356757227
C	2.505265353893	-3.465354731781	0.081718025536
C	-0.619045989415	-2.130317164074	3.353598730401
C	-1.378163705346	-2.213448875567	-1.455505845986
H	2.209958401096	-3.400266207187	1.121541864473
C	-1.189605152835	-2.285039197085	0.858097426598
C	3.602089806798	-4.596483363611	-1.721320732739

C	3.244428563665	-4.548359177241	-0.378815129484
H	-1.499222286406	-2.678447688517	3.665804137920
C	-2.564691385113	-2.931184102668	-1.430116575627
H	3.530150840672	-5.332688477205	0.311544567014
C	-2.372289436113	-3.007244634173	0.968483029374
H	-3.076119017723	-3.169852836512	-2.353671165882
H	-2.748618375556	-3.311829809360	1.936202768521
C	-3.057479058804	-3.327568079661	-0.194926686412
H	0.108387912391	-1.838443973449	5.353222152846
H	1.812106399489	0.232060079150	-5.802420108247
Cl	-4.547891538794	-4.237614458860	-0.100511755384
Cl	-2.256469495055	4.707275662930	-1.265951794870
H	4.176743406405	-5.427691075858	-2.114157459403
H	5.630274190821	1.672928913378	2.423574316429

8.1.4 [Fe(5-Cl-terpy)$_2$]$^{2+}$ (**H1**)

Fe	0.988511882673	-0.849815791917	-0.228904202325
N	1.257794550177	-0.484721399882	-2.086036670530
N	2.085638106022	-2.466126570044	-0.726393415727
N	-0.034759021715	0.886867216857	-0.387210446802
N	0.712618474086	-1.188707191549	1.631867843536
N	-0.725911444536	-1.914232691837	-0.331132121769
N	2.615380329006	0.078618588526	0.521625219145
H	-1.914872921078	4.192367862175	-1.128563854757
H	5.605957855518	1.701130463427	2.367781316556
H	-0.668118468735	3.025732697705	-2.926565043297
C	-1.387340715373	3.268883594227	-0.923843728603
C	4.664854551525	1.317858690390	0.466244057554
C	-0.688758443334	2.611065553018	-1.926333173338
C	4.768322330218	1.247246835608	1.850448556885
C	-1.387273410204	2.705006757999	0.343942745907
H	0.499076371774	1.824854310139	-4.410472373650
H	3.470048797844	0.759798467018	-1.235761984985
C	3.575914535880	0.721458169424	-0.158319192910
C	0.924569027151	0.931937019340	-3.969806487754
C	-0.023881063654	1.422630584519	-1.641151982318
C	3.779875816135	0.581469963191	2.565922051577
H	3.838237211249	0.507691647562	3.645208544193
C	-0.702942185530	1.519310682945	0.582726418269
C	1.683488737019	0.056787382560	-4.742530955667
C	0.729210657414	0.634255199147	-2.623253621008
H	-0.696747421448	1.075542771255	1.570070062912
C	2.712579966829	0.008486801797	1.880763856640
C	2.220245380344	-1.095259748593	-4.173728568667
H	2.806769005244	-1.779676754427	-4.773565501975
H	2.113151303489	-0.526847635851	4.615760915007
C	1.983313915254	-1.348650765092	-2.824772314045
C	1.603530185193	-0.710888331385	2.525045948760
C	1.407693110495	-0.911425583380	3.889970711229
C	2.446080841255	-2.503677849292	-2.041980923556
H	3.454646573305	-3.569884580448	-3.614811146480
C	0.278324631286	-1.612328337195	4.307287630432
C	3.182351065710	-3.560886230397	-2.566667740486
C	-0.385499055310	-1.868300699978	2.021265644580
H	-0.987466935440	-1.951369371630	-2.387959457529
C	2.467987988544	-3.472595380413	0.072347740068
C	-0.630886501946	-2.099580077259	3.372348033649
C	-1.394410199710	-2.263647574769	-1.435025382949
H	2.169534618762	-3.409052144389	1.111365074755
C	-1.211432909499	-2.293874026407	0.885327220148
C	3.566622753060	-4.602233922009	-1.730628147286
C	3.205330192114	-4.556556024854	-0.389007713994
H	-1.509013760998	-2.645049058365	3.694759151906
H	4.139710082793	-5.434201228118	-2.124119335150
C	-2.570422316010	-3.001605841052	-1.370096222247
C	-2.389510206056	-3.024965269005	1.000650487908
H	-2.764922304783	-3.313648256843	1.974242321002
C	-3.087214771211	-3.389132270877	-0.141891894857
H	-4.005887055592	-3.958754705861	-0.073831141635
Cl	-3.385194119186	-3.429075864565	-2.858278688746
Cl	-2.247526340271	3.465672111248	1.665071134900
H	5.411995383647	1.824978383398	-0.132347893834
H	3.486540554315	-5.343725812858	0.299916537369
H	1.858906791380	0.274243116405	-5.789794224802
H	0.104854844911	-1.779452079339	5.364147708797

8.1.5 [Fe(5,5"-diCl-terpy)$_2$]$^{2+}$ (**H2**)

Fe	0.992305302535	-0.846407569168	-0.226647918601
N	1.271827902244	-0.478491876052	-2.083960284366
N	2.078517935169	-2.473637705778	-0.731213249978
N	-0.037768369895	0.886606651834	-0.391350030272
N	0.724825333156	-1.182943910705	1.637679753564
N	-0.718186071277	-1.915682009506	-0.319324485186
N	2.618061389313	0.093317762801	0.522229934662
H	-1.928606722832	4.182140526579	-1.147131690022
H	5.610113100779	1.730475280669	2.358953221320
H	-0.644408216739	3.033447198554	-2.930106682964
C	-1.398071375831	3.261377014261	-0.937978300076
C	4.640874374004	1.348795414965	0.470096782534
C	-0.678877344778	2.613620692965	-1.932409966261
C	4.771097184041	1.272392331621	1.849229390684
C	-1.416202548058	2.690626754272	0.326511444526
H	0.551117169224	1.849345252547	-4.401517164132
H	3.452844488872	0.802028248622	-1.241478691333
C	3.558782176418	0.749823958902	-0.165322139530
C	0.968785115010	0.952563210040	-3.961465725629
C	-0.012177599491	1.427780356814	-1.642793132660
C	3.790905094533	0.593474977743	2.558784847464
H	3.863424212802	0.516531880179	3.636679349212
C	-0.725874552895	1.509081539154	0.570850762265
C	1.736402008043	0.081946493028	-4.730640966549
C	0.754006813632	0.645647640225	-2.619764089583
H	-0.730774030181	1.061252750834	1.556240568981
C	2.721400708688	0.016999883571	1.880083961703
C	2.262427325662	-1.075677216837	-4.163627685206
H	2.855566514397	-1.757244309490	-4.760257478032
H	2.135211993225	-0.511801869360	4.615676270747
C	2.004877469056	-1.338002389057	-2.820407875326
C	1.618095523143	-0.703682792509	2.527466354863
C	1.427757986823	-0.899678299194	3.893585465460
C	2.446178092101	-2.503302877521	-2.044433993348
H	3.444228687540	-3.582782023224	-3.617707589091
C	0.300054688916	-1.599688338566	4.316110895063
C	3.158297311603	-3.574735735989	-2.573514507715
C	-0.370292272740	-1.863470297913	2.032395476264
H	-0.982688627180	-1.961179491006	-2.375508876893
C	2.416365218596	-3.496288621855	0.061250491819
C	-0.611084662396	-2.090866471302	3.385069460912

C	-1.385004666168	-2.274678139107	-1.420984537619
H	2.116799206598	-3.445561833994	1.099817459869
C	-1.197247210037	-2.295976106633	0.899714005045
C	3.503129590337	-4.642290494805	-1.757527960528
C	3.118342910990	-4.594180247370	-0.424964343349
H	-1.487059037776	-2.636982241533	3.711809149925
H	4.052926607681	-5.487782046936	-2.153108642189
C	-2.553299727299	-3.024955718609	-1.351169965228
C	-2.367927614666	-3.037397013523	1.019900947361
H	-2.738382101692	-3.326512661768	1.995264688625
C	-3.063547830584	-3.412776169617	-0.120371252028
H	-3.975726049039	-3.992213265108	-0.048248890042
Cl	-3.364472055815	-3.468257943929	-2.835508949146
Cl	-2.307448105219	3.435630823115	1.635173958009
H	1.926984457907	0.307084782620	-5.773514359389
H	0.129451742264	-1.762997110118	5.373979386345
Cl	3.506300007502	-5.905548470940	0.665932777600
Cl	5.827683465783	2.198839181102	-0.493854325547

8.1.6 [Fe(6-Cl-terpy)$_2$]$^{2+}$

Fe	0.967284540890	-0.917755862404	-0.245988862879
N	1.360290744507	-0.463269603512	-2.072922741382
N	2.116449320056	-2.610822137953	-0.828933812158
N	-0.057450775711	0.813686534557	-0.385906216038
N	0.643550774923	-1.131034850718	1.636394653815
N	-0.836414777550	-2.037999041673	-0.286638592774
N	2.605367071207	0.015624327307	0.479168664413
H	-1.823290034262	4.193566092674	-1.063516626519
H	5.472578039739	1.631319041885	-0.212393316174
H	5.570122872143	1.727582258515	2.296630576711
H	-0.436054862189	3.123689379211	-2.824710831916
C	-1.331321156687	3.246862644503	-0.873238159175
C	4.690939271541	1.197675116173	0.399589046923
H	-2.058950964513	3.032500050872	1.153212812236
C	-0.557004556929	2.648484456231	-1.859023453297
C	4.741691257289	1.248402592524	1.787329925937
C	-1.463986102895	2.608057798419	0.353732870990
H	0.831214611675	2.046894496455	-4.249045344008
H	3.551439211124	0.528138815988	-1.291947664184
C	3.609686152716	0.576271779199	-0.212631830834
C	1.214106640282	1.115788592217	-3.851215625003
C	0.065305832432	1.432841612665	-1.593951862367
C	3.708721943621	0.672039299081	2.515467005184
H	3.722448243971	0.695695271611	3.598248642495
C	-0.811323751675	1.399176908674	0.556591031483
C	2.040615447401	0.306867154641	-4.624396163471
C	0.894474100406	0.704788678124	-2.559485216056
H	-0.895876634189	0.887246516068	1.505908656098
C	2.655068650735	0.061860587667	1.840712019560
C	2.530988357408	-0.884759010855	-4.101395628863
H	3.183704709738	-1.510834466642	-4.695621425879
H	1.993034128640	-0.212253675046	4.578241068414
C	2.161195245646	-1.253752967839	-2.809029989374
C	1.516737211304	-0.581826024002	2.505052407889
C	1.294197462635	-0.652170370795	3.878311717948
C	2.561332658056	-2.488075207756	-2.121389664894
H	3.652798091688	-3.336685799132	-3.766002774854
C	0.150487068010	-1.297594521935	4.337178662931
C	3.320914603403	-3.469316842849	-2.745162158940
C	-0.467324086603	-1.754454211584	2.067276448321
C	2.452530138037	-3.735488367027	-0.192252536808
C	-0.744605174725	-1.853229880912	3.429624131289
C	-1.568119911083	-2.525736022799	-1.291173032366
C	-1.293206321121	-2.297483866558	0.981794889185
C	3.646856581287	-4.626351926373	-2.050775127634
C	3.205948725802	-4.766716705329	-0.746727603685
H	-1.636125285331	-2.354051055373	3.783604408866
H	4.235581924206	-5.406626378831	-2.519184525871
C	-2.735436368884	-3.267609672464	-1.133156492743
H	3.434033148791	-5.646253762095	-0.159318844630
C	-2.447905606317	-3.030463383221	1.223695711686
H	-3.267998838013	-3.627847450473	-2.003047371578
H	-2.773430580300	-3.220435204214	2.237771658875
C	-3.178789737295	-3.525206015209	0.152134286930
H	-4.080964611716	-4.102487201959	0.317108895665
H	-0.047373530254	-1.363815187801	5.400913257783
H	2.312993623471	0.610310691912	-5.628317917153
Cl	1.927012432642	-3.964701780944	1.463781116760
Cl	-1.053706169180	-2.228447240895	-2.939448154879

8.1.7 [Fe(4'-Cl-terpy)$_2$]$^{2+}$

Fe	0.000373544202	-0.003303933042	-0.000104127523
N	0.001810735475	0.001432835896	-1.906723723939
N	1.373096263444	-1.442654574968	-0.332439895373
N	-1.371759630424	1.437824422706	-0.327189134348
N	-0.001037265213	-0.007957309958	1.906520987722
N	-1.440003150724	-1.376752063701	0.325142952988
N	1.440255119199	1.368327902200	0.334072286685
C	-3.211764622627	3.385461946234	-1.100599778512
C	3.131184000900	2.968706917756	-0.228970607932
C	-2.518651912270	2.655848324450	-2.059240161796
C	3.385049003410	3.207450299684	1.116615380339
C	-2.970778874753	3.127372644229	0.243780457241
C	2.153804138318	2.044043763448	-0.577419337907
C	-0.833274625649	0.886734485246	-3.951809386537
C	-1.605495112915	1.689938957839	-1.647822748017
C	2.652526030576	2.512647880512	2.071793474595
C	-2.045739507454	2.148718184063	0.587640089671
C	0.003657403823	0.008096052855	-4.631822373895
C	-0.809454905334	0.856311635012	-2.561119290820
C	1.688150119210	1.599894570859	1.655864490305
C	0.839755213492	-0.873792738091	-3.955018616951
C	0.813995834568	-0.850216069038	-2.564241941644
C	0.851647490667	0.802269666745	2.565133719710
C	0.877481815156	0.823813090677	3.955947571254
C	1.608586262114	-1.688472566044	-1.653929429746
C	-0.003677747800	-0.013985270014	4.631617023913
C	2.522194140448	-2.652563836143	-2.068681377688
C	-0.854909340360	-0.821144598396	2.559819980904
C	2.046004956861	-2.157727958759	0.579925214758
C	-0.883381520948	-0.848861117561	3.950474633457
C	-2.151749848856	-2.048631951790	-0.590567642580
C	-1.689840838625	-1.614613030084	1.645476348297
C	3.213907713713	-3.386822725506	-1.112585659539
C	2.971293774574	-3.134947323791	0.232682348720
C	-3.129201513662	-2.975391687834	-0.247905667463
C	-2.654396349120	-2.529753645315	2.055722375180
C	-3.385059184472	-3.220488932323	1.096157280634

H	-3.926152545064	4.143225991645	-1.402147225629
H	3.677892280009	3.484915429696	-1.008519476458
H	4.141702244554	3.921535313989	1.421666788795
H	-2.687364647085	2.838863134103	-3.113621174246
H	-3.485575569923	3.671676325029	1.025932626477
H	-1.478755765015	1.567592744119	-4.490801693851
H	1.936114950422	1.837849500257	-1.618117314449
H	2.832044840427	2.679860127374	3.127016828724
H	-1.837503900460	1.928085096541	1.627302399034
H	1.485912424822	-1.552063397407	-4.496432580085
H	1.556798978285	1.468155269771	4.498230649509
H	2.692474416050	-2.830473832319	-3.123692200710
H	-1.932480560381	-1.837575508996	-1.629947787929
H	1.836650012673	-1.941727573391	1.620343152207
H	-1.563766393651	-1.495555400081	4.488592527920
H	3.928590918251	-4.143284238139	-1.416690616917
H	3.485125064263	-3.682919836804	1.012912462733
H	-3.674358169730	-3.488365780749	-1.030662896343
H	-2.835470997692	-2.701896897557	3.109888644337
H	-4.141790979491	-3.936382624352	1.396768849597
Cl	-0.005599180366	-0.017711601623	6.381430440353
Cl	0.004714100158	0.012305610838	-6.381637357232

8.1.8 $[Fe(3'\text{-}Cl\text{-}terpy)_2]^{2+}$

Fe	0.999506522270	-0.847585705483	-0.227051572704
N	1.270660146713	-0.499090475528	-2.089613947987
N	2.099274360204	-2.469075468092	-0.674458047891
N	0.015045345894	0.883016928509	-0.391000605168
N	0.712318572844	-1.209405873944	1.630288701020
N	-0.714843050589	-1.887972389965	-0.388545372234
N	2.595185758802	0.074705073239	0.540346304582
H	-1.761430681921	4.256628921788	-1.005388338532
H	5.291606437112	1.949072633088	-0.181295858106
H	5.530958698295	1.842364737927	2.319800218474
H	-0.628957175910	3.095116733522	-2.856282261190
C	-1.265286030224	3.309908026480	-0.826025740570
C	4.583343043671	1.408217703592	0.434748350698
H	-1.735729101823	3.213490657171	1.283465761315
C	-0.627343744348	2.653223757943	-1.873217445388
C	4.711026776057	1.345515890873	1.813789150819
C	-1.254103928780	2.739124354580	0.437122931151
Cl	0.278585280729	2.185543600654	-4.905668669225
H	3.375239424435	0.789979769023	-1.234363853992
C	3.509266469678	0.758786348269	-0.160177029289
C	0.919244014841	0.816830132355	-4.021433604744
C	0.009055245279	1.433903976033	-1.643343605461
C	3.770573679379	0.634247019586	2.552488918456
H	3.867193484030	0.581960234251	3.624820220203
C	-0.600287687897	1.526446839953	0.609410403274
C	1.654349764919	-0.102445767273	-4.767368221140
C	0.728966342558	0.615484301606	-2.644556862554
H	-0.568042650961	1.055341466212	1.583664640644
C	2.712109474783	-0.000361094411	1.901754753427
C	2.192844285072	-1.221807802001	-4.158228311846
H	2.761498184407	-1.934153853242	-4.741568784534
Cl	2.483048432572	-0.656312631198	5.190509879489
C	1.977678251212	-1.403592325193	-2.795940272631
C	1.619441145354	-0.770371014562	2.540969851928
C	1.392798640693	-1.089682858233	3.890298211818
C	2.453971667545	-2.532391685539	-1.987701612173
H	3.478246174742	-3.617952933678	-3.539670283759
C	0.250425809997	-1.798971013449	4.258768776724
C	3.209259728125	-3.587207492720	-2.491318465552
C	-0.391955363848	-1.905846903778	1.965866460296
H	-0.965891251703	-1.875008972456	-2.441766937265
C	2.506146199550	-3.446721980591	0.147679013445
C	-0.654663336682	-2.211355215759	3.297424673932
C	-1.391562129904	-2.202843832572	-1.502036729913
H	2.211956939785	-3.365043954993	1.186363071141
C	-1.216187555694	-2.286462599295	0.812784155882
C	3.620865795568	-4.597752990547	-1.631490523606
C	3.265811478200	-4.524965832605	-0.289408088833
H	-1.537574685420	-2.765485002928	3.588192992953
H	4.211830217476	-5.425554900450	-2.006645731417
C	-2.584016324336	-2.914607751020	-1.476483975134
H	3.569711114532	-5.286244245369	0.418588886931
C	-2.408650668908	-2.997579801322	0.909039623505
H	-3.088256262453	-3.144943533756	-2.407080347307
H	-2.790627420259	-3.299352942869	1.876173545460
C	-3.102631016270	-3.316973721044	-0.251444797104
H	-4.032528540149	-3.871122528869	-0.196605014492
H	0.081118823527	-2.033099523196	5.302706002527
H	1.799708877230	0.065364511276	-5.827656588359

8.1.9 $[Fe(4'\text{-}SMe\text{-}terpy)_2]^{2+}$(**S0**)

See the Supplementary Information of Ref. 2.

8.1.10 $[Fe(5\text{-}Cl\text{-}4'\text{-}SMe\text{-}terpy)_2]^{2+}$ (**S1**)

Fe	0.977419512884	-0.849926974532	-0.230210637755
N	1.258350998616	-0.477872902823	-2.082812229268
N	2.064859289231	-2.471719395824	-0.737060553854
N	-0.057189200630	0.883253914489	-0.398505341949
N	0.718704979464	-1.179486637487	1.633026386532
N	-0.735835600292	-1.918492311196	-0.306161960308
N	2.613470252752	0.079197629122	0.508887629630
H	-1.953960125685	4.174040470920	-1.165644248429
H	5.619032351168	1.701058730441	2.333992287649
H	-0.610551142678	3.059632335192	-2.924581045594
C	-1.422251966390	3.254750489082	-0.953116199554
C	4.667820248391	1.311069449420	0.438417239460
C	-0.670108205997	2.625113494023	-1.934494355735
C	4.778050279500	1.247119047242	1.822250208194
C	-1.475716164131	2.664103378052	0.301349888188
H	0.609110090849	1.910010557344	-4.349257454063
H	3.463533331746	0.749608155887	-1.254897906535
C	3.573533257960	0.715766981371	-0.177832402071
C	1.014162457799	0.994321999991	-3.941701772829
C	-0.005719369698	1.438673375933	-1.642869556319
C	3.791266344892	0.586813912778	2.544565132984
H	3.855907302082	0.518254958095	3.623722040388
C	-0.778652090360	1.487714991022	0.551262710220
C	1.781043674244	0.127199888066	-4.729255815928
C	0.772858977671	0.659589190506	-2.614507491648
H	-0.806294772461	1.026601363133	1.530166219916
C	2.719699175423	0.013140432242	1.867544678639
C	2.272154958331	-1.058940067738	-4.155516662986
H	2.868974083626	-1.744145211782	-4.745612438781
H	2.171712944080	-0.511431035421	4.581616908961
C	1.991286855397	-1.334259895248	-2.826903187727

C	1.613988098358	-0.706383218272	2.519944371984
C	1.445471807065	-0.906240917473	3.885371300383
C	2.431124292817	-2.506621451251	-2.051360671550
H	3.431708007731	-3.581096433186	-3.624461560570
C	0.321055903147	-1.610569042070	4.333555634852
C	3.153522082513	-3.572471563860	-2.577829836774
C	-0.374574700237	-1.857937948966	2.043679314360
H	-1.006463978427	-1.969020133101	-2.360756489271
C	2.427312096055	-3.488791881151	0.057830231064
C	-0.603894145456	-2.091214914428	3.389397274313
C	-1.408577127265	-2.278048948348	-1.404551532979
H	2.124173032368	-3.425672940796	1.095548266243
C	-1.212136347724	-2.292447132557	0.916173109607
C	3.517106951373	-4.624590802129	-1.745708148796
C	3.149697903102	-4.581545619852	-0.405666143595
H	-1.479220460813	-2.642061149642	3.712813095400
H	4.079090960175	-5.463435410576	-2.140702085656
C	-2.580999283569	-3.020376333246	-1.328359270118
C	-2.387318026634	-3.026206234561	1.042299166351
H	-2.757393853222	-3.310583361100	2.019237278832
C	-3.089414440964	-3.400987373952	-0.094407615411
H	-4.005399800045	-3.973864079533	-0.017637441482
S	-0.025864998951	-1.945088992795	6.033869088389
C	1.455966082063	-1.338476496197	6.915993074044
H	1.547954245596	-0.254893803070	6.841106461681
H	2.351737977797	-1.839608974633	6.549937579117
S	2.181822911561	0.429211156125	-6.424891841462
C	1.574587359216	2.129355032187	-6.727722794878
H	0.486739755971	2.177909655779	-6.675913164628
H	2.037140342342	2.831884264728	-6.035094417045
H	1.287400283569	-1.615045116275	7.957432128068
H	1.898392039616	2.359338574005	-7.743238370766
Cl	-3.401095839921	-3.463925984078	-2.809031617676
Cl	-2.422409553324	3.378883443099	1.589044534788
H	5.414111241216	1.812139886106	-0.166229888419
H	3.414942625113	-5.377244677227	0.279938792169

8.1.11 [Fe(5,5"-diCl-4'-SMe-terpy)$_2$]$^{2+}$ (S2)

Fe	0.982968646504	-0.862358424561	-0.230419894790
N	1.264935882762	-0.479172694784	-2.083198759667
N	2.060622075339	-2.491578380292	-0.755976180210
N	-0.057752164851	0.868320534576	-0.393234476551
N	0.736494829664	-1.196428977477	1.635962845425
N	-0.726318005865	-1.935721650437	-0.296657779539
N	2.618310496718	0.077003591178	0.507100096024
H	-1.969717796250	4.152256475275	-1.150107689226
H	5.628546977355	1.708180766318	2.322973230733
H	-0.611117474075	3.056402669776	-2.909039405398
C	-1.433534938439	3.234973989358	-0.940216649029
C	4.644908208175	1.328101689489	0.440713811084
C	-0.672811458265	2.615939358542	-1.921747133498
C	4.785223607024	1.252068277503	1.818771099421
C	-1.490315841805	2.636377270434	0.310155328615
H	0.634232788420	1.931552114431	-4.330386946000
H	3.443161629201	0.783173116070	-1.260704938269
C	3.556716401744	0.731186555137	-0.185488217667
C	1.036228234175	1.011827630332	-3.928996592073
C	-0.003399629012	1.431827305396	-1.633949580084
C	3.808167028755	0.575456256559	2.534262219211
H	3.889219943120	0.498945408949	3.611432821676
C	-0.787146903772	1.462472110886	0.556599806001
C	1.811479343500	0.153927056248	-4.719313520141
C	0.783147574397	0.663249851216	-2.607519053096
H	-0.816857615796	0.995639089376	1.532667650517
C	2.732868893833	0.000810336794	1.863991255700
C	2.298684306795	-1.037758077266	-4.153453785496
H	2.901455222995	-1.716335481218	-4.745185575787
H	2.189686554037	-0.501833900320	4.579057588399
C	2.004915075124	-1.326503498185	-2.830937099882
C	1.632090290546	-0.718259761484	2.519695705399
C	1.463850733849	-0.903742225802	3.886572755415
C	2.430348750466	-2.510349443234	-2.069180458574
H	3.420144180456	-3.584703186678	-3.651806572285
C	0.337616320252	-1.600992707143	4.341292129218
C	3.131331525626	-3.583971417091	-2.608336718550
C	-0.355463710281	-1.873217213515	2.052436797800
H	-1.003502533657	-1.987943139666	-2.350322523038
C	2.383908657379	-3.527928970754	0.025237956822
C	-0.584956832968	-2.093683424999	3.400258319329
C	-1.398814186270	-2.301824107313	-1.392840545369
H	2.082185938263	-3.484534944086	1.063568123136
C	-1.193897916827	-2.314412090713	0.927937599828
C	3.461128073812	-4.665311550832	-1.803940099216
C	3.073718723049	-4.628554282037	-0.471822712875
H	-1.461292536446	-2.639853172597	3.728535661172
H	4.001713152630	-5.512815892978	-2.207936460707
C	-2.562230276689	-3.058317304368	-1.312490333014
C	-2.359745051463	-3.061685922202	1.057978551977
H	-2.722720096291	-3.350902030241	2.036172231503
C	-3.061113969598	-3.444795454646	-0.076569379375
H	-3.969314480462	-4.029453939001	0.003702850707
S	-0.014325543621	-1.907939580244	6.044271503120
C	1.433652929436	-1.225413762046	6.927024310037
H	1.491760320828	-0.142603547567	6.815040872491
H	2.351219724314	-1.709921867568	6.593992401538
S	2.228298052229	0.471716763771	-6.406515890678
C	1.611409129992	2.168888278035	-6.704881406655
H	0.522811700797	2.208480304209	-6.663440231517
H	2.062325941591	2.870742012986	-6.003975724775
H	1.256077928965	-1.471062869792	7.974770692032
H	1.943369722922	2.406598634096	-7.715959168095
Cl	-3.381004775170	-3.512314932120	-2.789583167330
Cl	-2.448615275436	3.336292848746	1.596334471557
Cl	5.825342382520	2.174438058943	-0.533759255852
Cl	3.442964313752	-5.958266859374	0.604026048423

8.2 Triplet state

8.2.1 [Fe(terpy)$_2$]$^{2+}$(H0)

Fe	0.979806122341	-0.848193874062	-0.222367350591
N	1.274316638514	-0.475034178247	-2.101464987578
N	2.159823199643	-2.566158569170	-0.810541594362
N	-0.127240846773	0.999384288417	-0.465372508758
N	0.711304238303	-1.193889876615	1.661637979093
N	-0.858778419941	-1.976154856289	-0.264870797546
N	2.706051806448	0.122317855895	0.620388539563
H	-1.984795906137	4.235791847994	-1.454338232978
H	5.550014955973	1.840167460421	0.111108824503
H	5.648142915229	1.631505174419	2.610579854519
H	-0.620463050319	3.020404292707	-3.123965770756
C	-1.463906747944	3.327440600605	-1.173227317135
C	4.772876427135	1.326495256045	0.664439461563
H	-2.136086197211	3.331330913803	0.884617883512

C	-0.699701645772	2.644480885326	-2.111600416441
C	4.822307523622	1.208263185716	2.049669394734
C	-1.550527892730	2.830441058619	0.123232650423
H	0.580615791418	1.857686566148	-4.431255049257
H	3.620273345073	0.836370385109	-1.090624083910
C	3.695019145902	0.767079024736	-0.010703571837
C	0.996256929105	0.960874226289	-3.990806110234
C	-0.044945238442	1.473933401568	-1.731554301500
C	3.800798477366	0.537230983013	2.712107587465
H	3.828981163130	0.431448225442	3.789521391727
C	-0.861447379665	1.664770045786	0.433478764187
C	1.774239152762	0.102114023719	-4.757966222888
C	0.760418921429	0.650826570071	-2.653082697786
H	-0.898907900895	1.247330441894	1.433915989709
C	2.747325115171	0.004000095915	1.970021289284
C	2.293221174172	-1.051942618275	-4.183734626628
H	2.897883295649	-1.728100235341	-4.774089228302
H	2.134854975094	-0.551144316539	4.646705590835
C	2.022123516001	-1.326005779558	-2.845101746055
C	1.608812057537	-0.724326340290	2.562451792358
C	1.422920471849	-0.934335754801	3.927037664588
C	2.499349676217	-2.519587266406	-2.121245620609
H	3.504051161547	-3.495439716433	-3.765572215302
C	0.304726500979	-1.640857671701	4.356588844290
C	3.239129786721	-3.540474076405	-2.716876330169
C	-0.380888746237	-1.883265265825	2.070218252469
H	-1.234664426514	-2.024959265707	-2.294676726423
C	2.552133655778	-3.611034421614	-0.072095635709
C	-0.605178834629	-2.123726589264	3.423936362322
C	-1.599158291349	-2.333081464964	-1.321066474696
H	2.261318715710	-3.602177204696	0.972607548759
C	-1.265663363481	-2.330535119408	0.978394791333
C	3.636515964179	-4.625382424210	-1.944953865437
C	3.290644996693	-4.664199156846	-0.597836238175
H	-1.475392605341	-2.678406030660	3.750174552804
H	4.210667360687	-5.429163234816	-2.392520142231
C	-2.776953149708	-3.059241574811	-1.196135256229
H	3.585534051195	-5.489651649427	0.038875038858
C	-2.438029796408	-3.058117227162	1.179328502944
H	-3.342616135536	-3.328481111356	-2.079929702860
H	-2.756293369616	-3.335098580947	2.176287856585
C	-3.199361680226	-3.427537653127	0.077119482140
H	-4.112681113094	-3.995566855266	0.214048927589
H	0.142612296888	-1.816182249077	5.413947087182
H	1.977840238012	0.332635960035	-5.797232925438

8.2.2 [Fe(3-Cl-terpy)$_2$]$^{2+}$

Fe	0.996883396824	-0.865840663939	-0.217553113254
N	1.243676671271	-0.488368740596	-2.104480720631
N	2.104869481808	-2.594838841563	-0.875374571953
N	-0.083735058965	0.993828183040	-0.385487354385
N	0.684091033341	-1.214347596057	1.653969707904
N	-0.830842725339	-1.981978299831	-0.281773242401
N	2.721794200891	0.053900512688	0.610858654627
H	-1.632495906240	4.432070547330	-1.196334276746
H	5.600771061945	1.715112634076	0.115857242315
H	5.621800501271	1.609403234040	2.623288686123
H	-0.478209040297	3.137509919575	-2.960604355980
C	-1.200505909998	3.464993405072	-0.965507953852
C	4.800873169976	1.233986035929	0.665828981790
H	-1.778805108290	3.473249162755	1.120673478104
C	-0.556011976893	2.736344915131	-1.958086744961
C	4.807493792223	1.173131237029	2.055557842127
C	-1.282326047275	2.939436247866	0.319542780039
H	0.218829599131	1.758636081428	-4.400178030198
H	3.691345107216	0.686734610239	-1.101625884209
C	3.736524503969	0.660458488361	-0.018423072919
C	0.696800039751	0.878312924121	-3.990929449446
C	-0.011573041993	1.492666713287	-1.641084721760
C	3.757604931522	0.543912935122	2.714995216391
H	3.752521291998	0.486170880782	3.796268752883
C	-0.705780435633	1.700092565840	0.565295973181
C	1.328810263985	-0.029062579555	-4.824970313534
C	0.664396838248	0.621960033306	-2.621706600025
H	-0.744755455307	1.257343794581	1.554743960546
C	2.720278429280	-0.008181647381	1.964906280163
C	1.929052829647	-1.162011291351	-4.286569154947
H	2.404135778221	-1.871096845524	-4.943184853039
H	2.004213548536	-0.364911669136	4.638392134090
C	1.895609837281	-1.373818891085	-2.908098128686
C	1.553072076932	-0.692492362163	2.553919339032
C	1.312575461119	-0.789326841303	3.922391300052
C	2.480938403412	-2.529395373390	-2.180559919241
C	0.155884041485	-1.420817388736	4.352133134104
C	3.358266904131	-3.513045004620	-2.671178252282
C	-0.437818047384	-1.869881827621	2.060973744669
H	-1.058812628266	-1.965491950280	-2.330841530017
C	2.524918678118	-3.577399443099	-0.077658786354
C	-0.725283674216	-1.962861198643	3.423389108310
C	-1.468836540171	-2.333835500558	-1.398092326014
H	2.179431130126	-3.550427090118	0.949227975251
C	-1.262465938592	-2.389922151914	0.941532954634
C	3.786350877387	-4.546597979709	-1.842360900978
C	3.363050497298	-4.588750861267	-0.525317733800
H	-1.625923132256	-2.441611866323	3.767689982065
H	4.458107634358	-5.301424951718	-2.233668160297
C	-2.601864473627	-3.133562307030	-1.384278509248
H	3.683511595081	-5.378647018531	0.142737300511
C	-2.388284329673	-3.229456643929	1.002817677167
H	-3.101914580496	-3.400678890915	-2.306989260424
C	-3.059508400417	-3.592300048578	-0.162018367078
H	-3.927990820452	-4.237276368170	-0.100269461115
H	-0.070145812463	-1.489115003011	5.410054403800
H	1.351317546378	0.136169061967	-5.896089006537
Cl	4.017321080654	-3.530040029320	-4.301057150096
Cl	-3.027883150571	-3.917027956603	2.488112296526

8.2.3 [Fe(4-Cl-terpy)$_2$]$^{2+}$

Fe	1.027066978359	-0.839938866195	-0.207090062122
N	1.318076836611	-0.457354584562	-2.087295148156
N	2.186716039088	-2.565638178597	-0.808025895576
N	-0.084734024233	1.017924860874	-0.447783572926
N	0.750653185409	-1.197110812889	1.671166415571
N	-0.823935748857	-1.932851326743	-0.265716227284
N	2.749006602186	0.115761997942	0.641573423035
H	5.610008005866	1.807340939277	0.143559652967
H	-0.724769178185	2.932375102693	-3.158183455841
C	-1.544159603564	3.238801388375	-1.195473479576
C	4.829563569869	1.295045748110	0.693396169701
H	-2.196146642938	3.284586779192	0.870308896899
C	-0.759812628416	2.584704400298	-2.134466051048
C	4.884208007084	1.156692602684	2.076331780555
C	-1.593503114100	2.791276719254	0.118605017323

H	0.632539867386	1.886537103368	-4.410227663285
H	3.664008500100	0.843745008855	-1.063361209301
C	3.742381731647	0.757994007089	0.015008071936
C	1.045565181823	0.987680009593	-3.971247585784
C	-0.037915819651	1.465901097687	-1.723794109719
C	3.857913412564	0.488294199757	2.734280548481
H	3.889839672796	0.365823953033	3.809801544407
C	-0.839234762070	1.672093201083	0.441679545798
C	1.836462628972	0.138351862772	-4.736171656900
C	0.796579634236	0.664314147718	-2.639802903423
H	-0.849357235267	1.285045667578	1.454703209031
C	2.795451987907	-0.021751651757	1.989884892261
C	2.351877304196	-1.018663971236	-4.165799798455
H	2.961517742708	-1.690103194033	-4.756384898610
H	2.171803505527	-0.590643044038	4.663928006793
C	2.071473021876	-1.302968926575	-2.830857117373
C	1.650936849160	-0.743860312680	2.576737950185
C	1.458055045351	-0.961359844670	3.939509844487
C	2.535497434263	-2.507305981823	-2.115789909903
H	3.535914987214	-3.477592388182	-3.765808195559
C	0.330748631205	-1.657369982896	4.361247189967
C	3.263729362387	-3.531323160592	-2.719461734702
C	-0.351243170288	-1.874279407876	2.073045992814
H	-1.206365024914	-1.958073980948	-2.297793355202
C	2.557407386462	-3.625691139726	-0.080378487952
C	-0.583693819642	-2.122021367328	3.423115028442
C	-1.570983330398	-2.265647801914	-1.324560845382
H	2.259839524096	-3.626141164812	0.962426973252
C	-1.237633509259	-2.295252073156	0.972250258247
C	3.638186676862	-4.632684926572	-1.959022385730
C	3.282240224320	-4.683822125529	-0.615270968829
H	-1.461833838586	-2.666833312761	3.744891628579
C	-2.761367134137	-2.969996445436	-1.216951216689
H	3.558292093188	-5.522680154276	0.012310063613
C	-2.419475416830	-3.005066065109	1.170873924336
H	-3.334211625262	-3.220497271384	-2.100422997352
H	-2.745177229786	-3.290952759999	2.161911942876
C	-3.176596563202	-3.337254918168	0.056553065851
H	0.163454157378	-1.837735118356	5.416841337261
H	2.050954950566	0.378150060803	-5.770999772395
Cl	-4.668314295340	-4.228006426052	0.261561606374
Cl	-2.485844337363	4.633318980603	-1.677900359461
H	4.201795914785	-5.439952349222	-2.413574790200
H	5.717794398839	1.561843197453	2.639034873692

8.2.4 $[Fe(5\text{-}Cl\text{-}terpy)_2]^{2+}$ (H1)

Fe	0.991682988403	-0.860296667672	-0.226083712229
N	1.275887412731	-0.476204789452	-2.105307124709
N	2.192289108586	-2.549680644889	-0.809047724527
N	-0.107437223574	1.004591319879	-0.459860640627
N	0.728174013924	-1.218661356976	1.658286296132
N	-0.854950824711	-1.960874923543	-0.273985955381
N	2.707589459345	0.137429860514	0.636132196957
H	-2.003059195029	4.222891992201	-1.440855625035
H	5.693727018470	1.552852664073	2.632234765249
H	-0.656694959753	3.007907529705	-3.118989944145
C	-1.469297087726	3.321076417832	-1.166161176901
C	4.766835511508	1.321006406091	0.699096658994
C	-0.713218995941	2.633502011033	-2.104731695835
C	4.853238129924	1.155925170177	2.075247495879
C	-1.524023823589	2.820534674267	0.128449876314

H	0.555909788341	1.848298610956	-4.436494066838
H	3.597532583811	0.913965209661	-1.068019224318
C	3.681307981780	0.797934406308	0.006555688422
C	0.977815022051	0.954274650871	-3.996237956964
C	-0.044258979536	1.468671972248	-1.730314856763
C	3.835326002011	0.469165537668	2.721268848376
H	3.886501317248	0.324809228046	3.793006100285
C	-0.828159443516	1.661312775584	0.450476155441
C	1.753615231157	0.096247160705	-4.766253896207
C	0.753943023607	0.646450882257	-2.655885150810
H	-0.854465833072	1.256718044170	1.455823862017
C	2.764820222996	-0.029306822459	1.979130608372
C	2.283609512241	-1.053050061576	-4.192369985830
H	2.888066333452	-1.727158180774	-4.785265821054
H	2.152711861101	-0.601739058232	4.648942440613
C	2.024195828977	-1.324616516086	-2.851026299868
C	1.627612723585	-0.761459467225	2.563121772210
C	1.440321392822	-0.978399855279	3.926310866846
C	2.515505490878	-2.510988090295	-2.124984841924
H	3.504809044708	-3.493820176166	-3.773750702637
C	0.317064143693	-1.679823089415	4.349979747152
C	3.253178089227	-3.532064352278	-2.721506728063
C	-0.370448569151	-1.900082128985	2.061659267201
H	-1.237223423109	-1.984741033074	-2.303688750784
C	2.598901902284	-3.586985811390	-0.067442105027
C	-0.596712831812	-2.148192935981	3.413666015117
C	-1.601232310626	-2.299706128705	-1.332188571308
H	2.321170731547	-3.571919285847	0.980645925675
C	-1.261363416952	-2.326668401617	0.966817254993
C	3.665780246504	-4.609156366559	-1.946155894028
C	3.336677450234	-4.639800921225	-0.594912881526
H	-1.472428987727	-2.696117600093	3.736299873915
H	4.238704522038	-5.413183350243	-2.394729963167
C	-2.785258448443	-3.016417199578	-1.211457011291
H	3.643940315764	-5.458730454247	0.044258602598
C	-2.440267967431	-3.043784629913	1.162779978656
H	-3.355596819031	-3.270544155095	-2.096646742820
H	-2.758772837884	-3.327719043505	2.157636010177
C	-3.208539597572	-3.393228137379	0.058714475693
H	-4.127557216311	-3.952659490095	0.192233311945
Cl	6.012778421489	2.182015713634	-0.178526098757
Cl	-2.465075238421	3.627373138478	1.365633131945
H	0.152449192296	-1.859880624467	5.406061943476
H	1.948073442183	0.324264653955	-5.807801381279

8.2.5 $[Fe(5,5”\text{-}diCl\text{-}terpy)_2]^{2+}$ (H2)

Fe	0.998177804882	-0.847552885050	-0.214087773401
N	1.298589677185	-0.458096837169	-2.094087854529
N	2.156050135185	-2.580934234811	-0.829366758923
N	-0.130981475015	0.995016470506	-0.458989148298
N	0.731220654347	-1.197852134204	1.671116803812
N	-0.836306742458	-1.984478121475	-0.252263454890
N	2.716441666030	0.127474133777	0.627131046352
H	-1.980928555953	4.241042730777	-1.434016813623
H	5.669561811408	1.630462382167	2.608139812812
H	-0.578912015632	3.058937524253	-3.090234123661
C	-1.460018255154	3.331250711507	-1.161013658450
C	4.766592076647	1.328101907226	0.673516926313
C	-0.673820756278	2.661709829823	-2.087693260257
C	4.838792285732	1.208463966992	2.055061081700
C	-1.562998697011	2.802076943211	0.118917465086

H	0.625143605352	1.900354928001	-4.404521491791
H	3.619261715493	0.857212178822	-1.091575228792
C	3.691940738480	0.775653655941	-0.013260251797
C	1.037238947608	0.999188699497	-3.969802126265
C	-0.024057694410	1.485608067446	-1.716601816304
C	3.819261567532	0.534726091021	2.712288303487
H	3.858632090926	0.427578339646	3.788882328404
C	-0.880941102350	1.634199987901	0.440149809878
C	1.820486522015	0.148285668722	-4.740053176044
C	0.791018341518	0.674297989154	-2.637328262489
H	-0.942633538739	1.208928697410	1.435243674513
C	2.761355502382	0.003257365222	1.976075450106
C	2.331497061505	-1.013833976865	-4.175788199909
H	2.938070751720	-1.684934474279	-4.769888542735
H	2.150354295796	-0.537936574478	4.654757449532
C	2.047873537070	-1.302683580510	-2.842863419637
C	1.626795467484	-0.723992995347	2.570760230932
C	1.440428629765	-0.926589347860	3.936085098319
C	2.504694650103	-2.511941790923	-2.136026776840
H	3.505406566090	-3.481400367741	-3.789199359812
C	0.322370520418	-1.632066201012	4.367529336061
C	3.225820900391	-3.538028393258	-2.745241756244
C	-0.359361673612	-1.887108077672	2.082100854681
H	-1.207828131471	-2.037481211628	-2.289854043890
C	2.506653021315	-3.646141617836	-0.105875825049
C	-0.585519628838	-2.121526751353	3.436660221801
C	-1.561832711752	-2.344545074356	-1.312836748182
H	2.212376570742	-3.662011819720	0.936868485728
C	-1.238697390507	-2.340544151272	0.991911635969
C	3.588088310410	-4.652734357828	-2.003250238031
C	3.217591115221	-4.701901053026	-0.665631077148
H	-1.455482987283	-2.675175465393	3.765134832481
H	4.144176596119	-5.463140857215	-2.459469318036
C	-2.730361120297	-3.084012847020	-1.172485835359
C	-2.405088564145	-3.078357681184	1.189124297333
H	-2.727274310594	-3.359838592155	2.183276366405
C	-3.164328367642	-3.459300115494	0.092318520815
H	-4.073366269020	-4.033783767824	0.223104131697
Cl	6.015451052638	2.168119622990	-0.218301185650
Cl	-2.548456701676	3.582843831050	1.337224691899
H	0.158621708047	-1.800799851952	5.425573130260
H	2.032665010265	0.390080125378	-5.774896548186
Cl	-3.638430553649	-3.529304029936	-2.600076177430
Cl	3.635335211670	-6.080874024593	0.328494391272

8.2.6 [Fe(6-Cl-terpy)$_2$]$^{2+}$

Fe	0.953657547939	-0.877890333340	-0.239884128488
N	1.361640691550	-0.441418487680	-2.093208772942
N	2.110699769160	-2.706319964419	-0.942435258684
N	-0.192379489245	0.898622498493	-0.478927696540
N	0.622494191766	-1.109970037428	1.664538398368
N	-0.945534664096	-2.126795507832	-0.206414873803
N	2.706135645112	0.020172192032	0.559810707382
H	-1.825433351026	4.296195461782	-1.301605181233
H	5.613128658154	1.607840033962	-0.013327987183
H	5.582724339597	1.717364964966	2.495019632898
H	-0.337348911408	3.196218477658	-2.946331615948
C	-1.369985899284	3.340592888080	-1.068521022591
C	4.797546823949	1.183411765301	0.559645173814
H	-2.256042703583	3.163774756869	0.898745061957
C	-0.537096497107	2.722730695146	-1.993587161676

C	4.776399488881	1.240617705317	1.948886193562
C	-1.610198397731	2.718293142730	0.151909449109
H	0.842113363931	2.066038780928	-4.283995065556
H	3.721327836062	0.502125633609	-1.176664369347
C	3.740197468319	0.564845220632	-0.094838364369
C	1.223453454022	1.135457212880	-3.884895389693
C	0.033972722059	1.492389262491	-1.676302654389
C	3.706487789966	0.678488465217	2.635612308193
H	3.677865517250	0.715494297146	3.717325552712
C	-0.996735704384	1.498894393099	0.406658837945
C	2.046006218447	0.327975248923	-4.660216274154
C	0.897321832657	0.728662009496	-2.593667814601
H	-1.155086752701	0.985970365280	1.348274185750
C	2.680539581400	0.070315805549	1.914295836389
C	2.516826942325	-0.868915397806	-4.138677380799
H	3.156215316549	-1.503546264220	-4.737412815498
H	2.002237059669	-0.206770867566	4.604364722893
C	2.149423883140	-1.241170127762	-2.845544409363
C	1.507481129565	-0.569388242858	2.537257912558
C	1.295898465387	-0.644744324638	3.911418849063
C	2.560679890594	-2.506591821722	-2.211688038602
H	3.700123265864	-3.275394318008	-3.874811618948
C	0.161829144742	-1.292470628893	4.385756618161
C	3.349877682186	-3.448233827814	-2.866654182159
C	-0.475343321051	-1.754112745598	2.117647299041
C	2.450287130754	-3.841196755412	-0.343381887197
C	-0.730051465980	-1.857124194777	3.485146302274
C	-1.697143803444	-2.638708107710	-1.172796726575
C	-1.340403874701	-2.340353808531	1.078830231245
C	3.686868403770	-4.625531247264	-2.211140334303
C	3.233445559101	-4.836105440031	-0.918286039113
H	-1.609001109366	-2.372919288634	3.847361085835
H	4.299198252793	-5.372592518054	-2.703238440239
C	-2.850239903328	-3.387357797559	-0.964623990129
H	3.473072914848	-5.736511309716	-0.368138169651
C	-2.482645063632	-3.076859930109	1.382406684645
H	-3.411469965670	-3.779057300591	-1.802404967979
H	-2.781548879092	-3.243869573295	2.408394256995
C	-3.240670128596	-3.608809097146	0.347131580892
H	-4.130055257322	-4.190166536776	0.561304380269
H	-0.024031322500	-1.361165669858	5.451481085593
H	2.320849134072	0.630982034097	-5.663532211730
Cl	1.872221704588	-4.113817453817	1.294591550348
Cl	-1.205909354921	-2.350271384820	-2.836323054410

8.2.7 [Fe(4'-Cl-terpy)$_2$]$^{2+}$

Fe	1.040414352792	-0.789040598895	-0.202603558096
N	1.299037140192	-0.395588532404	-2.080078233029
N	2.223273949936	-2.491214750418	-0.827195235180
N	-0.051920016227	1.071334130146	-0.400255469712
N	0.773485129749	-1.169106255738	1.672348421129
N	-0.802296100487	-1.905384231886	-0.265516960222
N	2.771845835129	0.159369937882	0.659809254058
H	-1.915137509557	4.328204018332	-1.307312340785
H	5.629071796478	1.863626436936	0.185285466262
H	5.734716889841	1.590559247393	2.677615153410
H	-0.641605686025	3.099154190593	-3.035522841454
C	-1.392168364496	3.414413625359	-1.049143061498
C	4.849228104431	1.343038057607	0.728122874318
H	-1.969896060389	3.426863706883	1.036475118692
C	-0.677541102063	2.723207779156	-2.020644052230

C	4.903037140242	1.189607221309	2.109229199137
C	-1.425272046258	2.919147239383	0.249761861281
H	0.461078591776	1.905111527345	-4.399442880233
H	3.686143618413	0.908567754851	-1.035516244235
C	3.764006426547	0.810814864062	0.041861238519
C	0.907362335488	1.020536411733	-3.966548785894
C	-0.018370620355	1.547457907555	-1.667881574630
C	3.877262518301	0.511737124064	2.758561715956
H	3.908984981505	0.378142467871	3.832801418902
C	-0.737717069141	1.744505878132	0.529836101810
C	1.653438609078	0.149607361439	-4.748919256861
C	0.748754392483	0.719315876186	-2.617188578417
H	-0.737492930189	1.327104222649	1.530941520581
C	2.818010278116	0.008360503971	2.005942923751
C	2.224871679328	-0.990319763857	-4.201819881119
H	2.802833054047	-1.664334547755	-4.819173150246
H	2.197263175852	-0.579478366890	4.672146082580
C	2.022384923238	-1.243819180667	-2.849122488990
C	1.672533966391	-0.721958224662	2.581498199786
C	1.487256387280	-0.944000962616	3.942440276055
C	2.543229319367	-2.429180614212	-2.142189443616
H	3.551610971436	-3.366093315218	-3.807352172224
C	0.356776820946	-1.642838674721	4.344233507862
C	3.298993161582	-3.425818562410	-2.756301603438
C	-0.326762168723	-1.851503367302	2.068902545228
H	-1.173279486738	-1.923081209590	-2.296945226021
C	2.646343521905	-3.533944429876	-0.103564040442
C	-0.563280330704	-2.110650323475	3.415349763868
C	-1.543862594264	-2.238159768352	-1.328019858341
H	2.369674251224	-3.540618320215	0.944894674765
C	-1.217675019128	-2.272482779127	0.971145133731
C	3.729190156237	-4.508873240821	-1.998877165409
C	3.399849300404	-4.566837841111	-0.648879822470
H	-1.439614820209	-2.657394023776	3.735795905127
H	4.316179124162	-5.295201352777	-2.460239166078
C	-2.731629505120	-2.950463400410	-1.215737794674
H	3.719144573800	-5.391880321735	-0.023609724836
C	-2.399730525269	-2.985644730693	1.160151393426
H	-3.297610061274	-3.200312804983	-2.104994626286
H	-2.725830532150	-3.272204653227	2.151851766698
C	-3.163363763988	-3.329258092045	0.050617315899
H	-4.084987435228	-3.885923090219	0.177155621253
Cl	0.085086959866	-1.934344733432	6.047202752022
Cl	1.872426290421	0.488795544678	-6.450086689443

8.2.8 [Fe(3'-Cl-terpy)$_2$]$^{2+}$

Fe	0.990710384523	-0.860925872020	-0.215689486289
N	1.279494079092	-0.502513112256	-2.106865996272
N	2.170951881981	-2.594278433082	-0.768735388301
N	-0.076860227520	0.971685269058	-0.458573734393
N	0.687960811146	-1.217754329241	1.661034714095
N	-0.812958665566	-2.012554214854	-0.344912756674
N	2.664551518440	0.090035840205	0.641807458697
H	-1.295014189124	4.564102241421	-1.103038352796
H	5.329449198910	2.049578411638	0.043584934380
H	5.309072558299	2.125673486809	2.555737893356
H	-0.146033416273	3.312787291620	-2.877111686708
C	-0.965023366723	3.548275381722	-0.917297609179
C	4.588220274792	1.511750598844	0.622369105061
H	-1.681239974628	3.464151433320	1.124165922613
C	-0.322137104870	2.837380003844	-1.926462149849

C	4.574427941402	1.545057386207	2.009073098006
C	-1.175667958038	2.949020433052	0.316473073592
Cl	0.170069126349	2.122554380229	-4.952282026903
H	3.557097458801	0.735657499972	-1.104141892954
C	3.602135447551	0.774992055050	-0.021367779880
C	0.875241655729	0.791955089668	-4.056006479091
C	0.092548545276	1.525937409191	-1.686834912092
C	3.605241995275	0.829178038212	2.707055800577
H	3.597830939071	0.867413541503	3.783765225476
C	-0.698569333644	1.658370452686	0.504189402907
C	1.564623541774	-0.145786452046	-4.818797387595
C	0.756791937324	0.627047922268	-2.666078675379
H	-0.813701798240	1.158525814683	1.459604812834
C	2.658996221404	0.081637451926	2.002170254525
C	2.115338490111	-1.255265749389	-4.209387893793
H	2.653394096291	-1.981538436547	-4.803384551594
Cl	2.421771407190	-0.636896708403	5.249472482278
C	1.947869031498	-1.424224518918	-2.837751784151
C	1.567679828564	-0.739867079104	2.590158955562
C	1.349053825391	-1.079744855931	3.936276498435
C	2.462170621394	-2.585890252504	-2.089439303225
H	3.435186607972	-3.596395544886	-3.735220585539
C	0.237378599996	-1.835242469108	4.300802298264
C	3.205482437523	-3.609409576457	-2.677475573770
C	-0.391623877956	-1.959288448788	2.001680342421
H	-1.142529076895	-2.029943752769	-2.384239719859
C	2.615346531764	-3.601115167773	-0.008301776456
C	-0.643262908456	-2.275063992481	3.333235374435
C	-1.524848507813	-2.357288024348	-1.423963956486
H	2.363016282451	-3.561580541409	1.045784897879
C	-1.243329891259	-2.392172377221	0.880693004237
C	3.657195032183	-4.655993693201	-1.883142829390
C	3.362234092972	-4.653870693218	-0.523569562330
H	-1.503970281132	-2.864842620362	3.618243201382
H	4.236802023756	-5.459946312887	-2.323115926213
C	-2.697612515359	-3.096856285909	-1.338465505023
H	3.702349635973	-5.447582868659	0.130532332550
C	-2.413260649081	-3.133700994798	1.043241931519
H	-3.239735590974	-3.356871628805	-2.239522392345
H	-2.753222406673	-3.430998920990	2.026833857439
C	-3.145248539557	-3.491555690173	-0.082167214264
H	-4.055746793457	-4.070351048198	0.024393302061
H	0.076524489029	-2.085667270603	5.342143694598
H	1.656692522039	-0.004865495790	-5.888951980385

8.2.9 [Fe(4'-SMe-terpy)$_2$]$^{2+}$(**S0**)

Fe	0.977046580686	-0.844553813036	-0.222714871987
N	1.275202916071	-0.464810573201	-2.098759183739
N	2.141727825769	-2.570286539967	-0.829081946032
N	-0.139442410888	1.000236364019	-0.471817359360
N	0.716460323286	-1.183173439805	1.663116658844
N	-0.863542500276	-1.979429604523	-0.243251519460
N	2.713238088897	0.119077846559	0.616649000385
H	-2.022231195384	4.217446958349	-1.477135556135
H	5.565808020134	1.820405729894	0.098363439096
H	5.659381165559	1.630860458831	2.599726954883
H	-0.613716477639	3.029824976102	-3.126728835500
C	-1.494125414717	3.315008876661	-1.190683996215
C	4.785610174607	1.314489761018	0.654421691938
H	-2.205969049309	3.296078147950	0.853163440239
C	-0.704072190745	2.647140215459	-2.118106037922

C	4.833168763392	1.206527668975	2.040286247475
C	-1.598511628525	2.808342366535	0.100475296162
H	0.630752365770	1.918059858698	-4.380024624007
H	3.631746019696	0.817254911088	-1.098038741555
C	3.705567442271	0.755188586737	-0.017670137613
C	1.042020042518	1.006604862109	-3.971094940878
C	-0.040996896383	1.483621647364	-1.733182057071
C	3.808334539171	0.543738430821	2.705533228253
H	3.836838384682	0.446875506441	3.783638705256
C	-0.898993667724	1.650390627473	0.416905814173
C	1.825527749477	0.157023823669	-4.755974227825
C	0.783693082935	0.669107887223	-2.647399708158
H	-0.949388660056	1.225962924226	1.413765041492
C	2.754339726650	0.008380236965	1.966689248221
C	2.315696729721	-1.022837489459	-4.176505807835
H	2.923843630171	-1.701643101142	-4.761600534012
H	2.169645490478	-0.520511010568	4.620707027726
C	2.024435863514	-1.309430616718	-2.852356618174
C	1.612986326787	-0.716100309794	2.562002308120
C	1.442958081971	-0.917187702698	3.926846777649
C	2.488209853061	-2.516958966595	-2.137659112681
H	3.493985267047	-3.491842379584	-3.782718618044
C	0.325431432450	-1.622150216703	4.382545014517
C	3.221332455871	-3.540863975148	-2.736147094307
C	-0.375432545773	-1.866875683941	2.089011379953
H	-1.238700666423	-2.048915864454	-2.272039284450
C	2.518479763223	-3.624883052426	-0.096353279249
C	-0.593942215699	-2.101389593048	3.436992797467
C	-1.601155608379	-2.352132041724	-1.296090376335
H	2.221420410426	-3.619781799612	0.946591482900
C	-1.265591919968	-2.326819674849	1.003439364924
C	3.602955416779	-4.635844655883	-1.970084027444
C	3.248579536534	-4.681959986774	-0.625490418548
H	-1.465452302537	-2.657004962540	3.760686654455
H	4.171940992297	-5.441934821529	-2.420141227350
C	-2.771927844729	-3.088189463484	-1.164620873029
H	3.530654877434	-5.515538623216	0.006576323879
C	-2.432051465758	-3.062030170246	1.211047763724
H	-3.334837158379	-3.371432546690	-2.045850191745
H	-2.748441631623	-3.334386160633	2.209959957555
C	-3.190533669768	-3.448040369657	0.112316677161
H	-4.098574267879	-4.023159873235	0.254743403835
S	-0.015546189511	-1.956886931720	6.083888263569
C	1.467467338858	-1.344670372958	6.960701063462
H	1.556989435662	-0.261114368997	6.882276306964
H	2.363510789578	-1.845334140237	6.594511708076
S	2.243387264243	0.468207901811	-6.445134599677
C	1.593853793264	2.150512147707	-6.759220455325
H	0.504503184652	2.168908221137	-6.718919831918
H	2.029707117319	2.867678804391	-6.064288645242
H	1.302362690947	-1.618043863427	8.003530703727
H	1.921402014215	2.386476842008	-7.772108737260

8.2.10 [Fe(5-Cl-4'-SMe-terpy)$_2$]$^{2+}$ (S1)

Fe	0.986371936952	-0.862248163194	-0.226017263970
N	1.275750515930	-0.468940242271	-2.102308827333
N	2.171069261502	-2.561700687681	-0.831204858398
N	-0.120072242636	1.002122048301	-0.464270646850
N	0.727436462187	-1.210179642288	1.659811822626
N	-0.858406645596	-1.974435986610	-0.253491371875
N	2.710386252200	0.127826341574	0.631714446946
H	-1.975835881510	4.245278547411	-1.440386534201
H	5.683853726858	1.574228897785	2.625732786495
H	-0.588097954942	3.054813079784	-3.100119871172
C	-1.454024329778	3.336212736837	-1.166810128760
C	4.772600680741	1.307119313645	0.689176823614
C	-0.675834506973	2.661025488471	-2.095803810865
C	4.847955385140	1.167876389037	2.068703258718
C	-1.547966859505	2.811933050172	0.115811921032
H	0.600220238762	1.906163296486	-4.384921595884
H	3.613854113973	0.873697325739	-1.078689432764
C	3.690171471050	0.775543292914	-0.001840613509
C	1.019246925091	0.997584614762	-3.976793729385
C	-0.024609620352	1.485773471385	-1.724896825002
C	3.823976534962	0.494044015883	2.718186913643
H	3.865654652140	0.372488281188	3.792913153251
C	-0.862851174703	1.646038031156	0.437266182771
C	1.792128358295	0.144346427017	-4.768667897422
C	0.780645395216	0.664983684106	-2.648033135844
H	-0.916067373237	1.225129067498	1.434775041858
C	2.759901097799	-0.018211986214	1.977166505500
C	2.293456555354	-1.031529497346	-4.189618230389
H	2.896742511053	-1.710740319996	-4.779238432162
H	2.170163686212	-0.553135843375	4.624940245838
C	2.019035673914	-1.313922947801	-2.861278870589
C	1.619594766034	-0.745792085282	2.564130018516
C	1.442524263961	-0.943370903787	3.928176331551
C	2.498008023463	-2.515210240842	-2.145804770357
H	3.485069032000	-3.495143284372	-3.798556456013
C	0.316402274736	-1.638047624115	4.378371832282
C	3.228089985468	-3.538577326862	-2.747761917565
C	-0.370367244680	-1.887388717171	2.080352015864
H	-1.236073601283	-2.020256447154	-2.282945287590
C	2.564369684659	-3.609401495360	-0.096853503194
C	-0.599198935350	-2.115566368406	3.427546995162
C	-1.596337975941	-2.335830249772	-1.310181949692
H	2.282507391928	-3.599491286839	0.950234377234
C	-1.258933872107	-2.337993813121	0.989665159722
C	3.627349937182	-4.626223663976	-1.979873347923
C	3.292955921485	-4.665421908004	-0.630142488109
H	-1.478692154061	-2.660547649878	3.747545096751
H	4.194406253134	-5.431950444119	-2.432950403496
C	-2.765698910604	-3.075379488191	-1.186037240833
H	3.589394343387	-5.492890813898	0.003283448311
C	-2.423415625591	-3.077382255737	1.189301344476
H	-3.329146423631	-3.348649640295	-2.070027244489
H	-2.737760457126	-3.362808745329	2.185171298313
C	-3.182600249510	-3.451066743757	0.086615036904
H	-4.089563474616	-4.029268373532	0.223055610258
S	-0.041524544888	-1.956147577524	6.077923129631
C	1.403815849165	-1.278483177509	6.969140934569
H	1.462174603670	-0.195115127796	6.862782591927
H	2.322552936297	-1.760883964660	6.636390537727
S	2.185553985114	0.446204195009	-6.464413087089
C	1.536256007478	2.128710872260	-6.778006148948
H	0.447818508866	2.150642484305	-6.720197188707
H	1.985473976146	2.848040578191	-6.094024065372
H	1.223327643560	-1.529455895425	8.015099674448
H	1.848664202424	2.357238740343	-7.797402240381
Cl	6.028532502692	2.147363450241	-0.194128254186

Cl	-2.525672479559	3.598270767990	1.337837644379

8.2.11 [Fe(5,5”-diCl-4’-SMe-terpy)$_2$]$^{2+}$ (S2)

Fe	0.993707650353	-0.865454922769	-0.218979346204
N	1.290719916606	-0.461887523266	-2.096235386724
N	2.136620910796	-2.603950999549	-0.866266095499
N	-0.145432216146	0.975018545302	-0.459856860920
N	0.735565563119	-1.208525191301	1.668421558812
N	-0.840841698167	-2.011935751353	-0.233225038795
N	2.724249759057	0.097784892106	0.617050565709
H	-1.984771964743	4.232961039888	-1.416099128938
H	5.681777277150	1.605509992521	2.588736629832
H	-0.556562688818	3.077520052137	-3.065570423126
C	-1.467178484523	3.319936343973	-1.147849955173
C	4.784169191371	1.282288849452	0.654490689798
C	-0.666019695587	2.664377374312	-2.071317210247
C	4.850491583456	1.181943812839	2.037696134657
C	-1.590559966735	2.770734147321	0.121568471100
H	0.639679025973	1.945565951028	-4.350890781114
H	3.637503169069	0.798584176400	-1.107700076221
C	3.706797270245	0.729986370251	-0.028286730495
C	1.053913446661	1.030993901203	-3.952038129623
C	-0.021330619908	1.483661822305	-1.708318248003
C	3.823444905715	0.524676478510	2.699539516666
H	3.858129905892	0.434396372082	3.777599783728
C	-0.910492671806	1.599812845320	0.436566202259
C	1.834626077804	0.189561092668	-4.749342249832
C	0.800705323910	0.679225580605	-2.631209005561
H	-0.986591764384	1.159846893381	1.424201755115
C	2.765541149763	-0.010696612572	1.967240465742
C	2.324147360886	-0.997688291617	-4.184095986341
H	2.927699523074	-1.671553132300	-4.779466873167
H	2.170048754390	-0.499637949170	4.625557827848
C	2.035098178148	-1.298657253039	-2.863082847791
C	1.625162635075	-0.728011911560	2.566731591157
C	1.446427589486	-0.904870349641	3.933126523294
C	2.488603875308	-2.520825382146	-2.171131750332
H	3.495785824455	-3.473795444330	-3.830896260371
C	0.322667160105	-1.598687463296	4.392795298590
C	3.211303696785	-3.540725312592	-2.788830542263
C	-0.356220504928	-1.889140936093	2.098159809231
H	-1.213450769509	-2.085676162517	-2.269726407786
C	2.483389514650	-3.677900577991	-0.153573242174
C	-0.586197599854	-2.099799291177	3.447774007871
C	-1.563437490662	-2.389364624964	-1.290127905921
H	2.185129749919	-3.704556517971	0.887887619866
C	-1.237098133354	-2.362465901228	1.014403039166
C	3.570285763237	-4.664016328599	-2.057945247401
C	3.195443711071	-4.727788189156	-0.722231638798
H	-1.461968546562	-2.646779306066	3.774502802247
H	4.127906533786	-5.469613220436	-2.520825556209
C	-2.722426531420	-3.141833172650	-1.142476640266
C	-2.393707125152	-3.113740912343	1.218780757883
H	-2.711120642883	-3.394037358060	2.214888702608
C	-3.149632684731	-3.512730127303	0.125903918475
H	-4.050693153545	-4.098479447638	0.262134094237
S	-0.040805697455	-1.890622573149	6.094529095286
C	1.378774678539	-1.160660970795	6.985082476239
H	1.413579290659	-0.079123000158	6.852968963538
H	2.312219288571	-1.630223493308	6.675461605650
S	2.246904962847	0.515498072465	-6.434507296321
C	1.586342782771	2.195419053535	-6.736675915499
H	0.497051418775	2.206402019403	-6.692308268197
H	2.021069415804	2.911430024931	-6.039914289743
H	1.190724384230	-1.390790330680	8.034565785430
H	1.908711962242	2.437958910476	-7.749663620492
Cl	6.043861172862	2.098082605855	-0.244366553055
Cl	-2.600249004554	3.530179884117	1.333297309034
Cl	-3.627343387926	-3.610220953491	-2.565202039638
Cl	3.609406088737	-6.118374158109	0.257954587172

8.3 Quintet state

8.3.1 [Fe(terpy)$_2$]$^{2+}$(H0)

See the Supplementary Information of Ref. 2.

8.3.2 [Fe(3-Cl-terpy)$_2$]$^{2+}$

Fe	1.025513485388	-0.888627331974	-0.236420667967
N	1.223720566257	-0.537042914781	-2.346911640004
N	2.161453680870	-2.597125764079	-1.064360867165
N	-0.164707062657	0.928175949047	-0.628866775709
N	0.670323360333	-1.210275233149	1.861007966992
N	-0.834741086741	-2.085596063197	-0.079705525662
N	2.743672098906	0.019669500288	0.789512446625
H	-2.326369850320	3.963824088351	-1.637789696908
H	5.574209242735	1.770243317506	0.315825613369
H	5.619162975848	1.597645775417	2.820286563337
H	-1.022711548445	2.737759755693	-3.342958756396
C	-1.717326869990	3.114675160120	-1.348711658016
C	4.793023766442	1.253047055345	0.859779045717
H	-2.210917492083	3.207549813545	0.756841759571
C	-0.983623082059	2.424653026973	-2.307559203900
C	4.813879477289	1.153529248767	2.245831878597
C	-1.657281119544	2.699677784158	-0.023377348877
H	0.133391015675	1.633040393665	-4.676893400262
H	3.682496304589	0.725200429433	-0.912800992331
C	3.736576198033	0.669656106457	0.169687578071
C	0.642530695253	0.782923557727	-4.243374282219
C	-0.212908219636	1.330602892893	-1.917939199847
C	3.786003788417	0.475917574622	2.894398141387
H	3.790298637223	0.390553516970	3.973840412205
C	-0.866007973106	1.601079198245	0.290855449942
C	1.365764407477	-0.083010657765	-5.047589452477
C	0.576928339727	0.515558173597	-2.875505523327
H	-0.793172903835	1.243047404301	1.312544264550
C	2.758649391498	-0.082816916007	2.137374774844
C	2.011538857067	-1.183290948574	-4.491454445640
H	2.546076411110	-1.862398670118	-5.133591263138
H	2.270631787381	-0.824480329289	4.796801787927
C	1.945331002814	-1.384463978862	-3.112102937351
C	1.618720678751	-0.826382884630	2.732665717545
C	1.515442081756	-1.137266241315	4.087702042732
C	2.575708017534	-2.508066480192	-2.353438056587
C	0.424910713155	-1.885381783698	4.509712782742
C	3.511564988779	-3.447124488455	-2.819883621026
C	-0.416723990784	-1.900459861758	2.261184814230
H	-1.039653854358	-2.276104547030	-2.123988205953

C	2.592923236562	-3.570746595435	-0.262226129653
C	-0.546934679252	-2.282791151390	3.598374771375
C	-1.534409142800	-2.410423169789	-1.168235391873
H	2.203878511479	-3.575589463774	0.750006462198
C	-1.362757995004	-2.233917453795	1.159966886345
C	3.963505448418	-4.465106543186	-1.982472662531
C	3.495710256548	-4.538048705838	-0.683212171404
H	-1.376389092406	-2.885058081630	3.931879244534
H	4.683898057868	-5.183762513422	-2.354909270263
C	-2.833568109365	-2.892622451306	-1.102236457825
H	3.826622092982	-5.317872532434	-0.008404938013
C	-2.695871804546	-2.657596961880	1.277669550993
H	-3.369088369011	-3.160687706637	-2.004608846558
C	-3.426884411908	-2.999755842640	0.143750151284
H	-4.451174114915	-3.337280020568	0.245188067596
H	0.333609174733	-2.174181236861	5.550684878708
H	1.424462854361	0.088995124480	-6.116605480504
Cl	4.222808611427	-3.427404800141	-4.427977436258
Cl	-3.564319681922	-2.730356626999	2.804980051226

8.3.3 [Fe(4-Cl-terpy)$_2$]$^{2+}$

Fe	1.016011433300	-0.848072341619	-0.218430646993
N	1.350143654339	-0.407760002972	-2.291839127607
N	2.149549805022	-2.582534320362	-1.030794799953
N	-0.167252778164	1.004159613395	-0.662258671707
N	0.729476694031	-1.210552873746	1.879532307073
N	-0.878023253009	-1.985135481204	-0.050348925550
N	2.757461064361	0.070026861310	0.805166452418
H	5.652802078741	1.701391792943	0.296482871120
H	-0.638042628275	2.996816540377	-3.348135681332
C	-1.599205571860	3.233611934476	-1.441233837637
C	4.860990431219	1.211176751278	0.850043178296
H	-2.401737847059	3.202395073233	0.570441657249
C	-0.740526990566	2.616838671043	-2.340791632292
C	4.907183587130	1.088612472984	2.233509785014
C	-1.739362976312	2.741869612991	-0.151210610343
H	0.837057836359	2.049523136960	-4.531158435433
H	3.692408832167	0.760224705638	-0.904867718407
C	3.764723727004	0.685940483388	0.175259903157
C	1.211756837161	1.128312065709	-4.104620246831
C	-0.035624602242	1.494297185486	-1.914174100626
C	3.863936449141	0.446943329163	2.893740922968
H	3.889797012374	0.336985235983	3.970579152484
C	-0.997605066846	1.617946431081	0.187400963224
C	2.053936096112	0.308704712325	-4.848459713737
C	0.870566997131	0.734380208881	-2.811899554517
H	-1.081834393695	1.195373611360	1.182963306777
C	2.796040222993	-0.051704065026	2.151306991568
C	2.531142073356	-0.877104290378	-4.303082515841
H	3.182653565101	-1.517642879694	-4.883004160586
H	2.179673194074	-0.567334176344	4.845436276246
C	2.151218139107	-1.217967852602	-3.004910315979
C	1.636516792988	-0.751592112680	2.759721263490
C	1.459657657492	-0.940894740742	4.129053433978
C	2.564512217085	-2.463964701160	-2.312272127979
H	3.633738767559	-3.363522648575	-3.958255822918
C	0.327389950236	-1.621222129000	4.567213523930
C	3.311419975897	-3.464732790107	-2.930051841054
C	-0.367122585302	-1.870162933471	2.289667735297
H	-1.306109190915	-2.065133754874	-2.073250495769
C	2.471408142247	-3.684032491177	-0.342794570115
C	-0.598243132474	-2.096500514955	3.646713873317
C	-1.647679823125	-2.349675009532	-1.083737733457
H	2.118729662645	-3.736162195267	0.681750268258
C	-1.268027608749	-2.314675794472	1.201015276242
C	3.640765648884	-4.607773809611	-2.209157848293
C	3.217601914817	-4.721709677756	-0.890472107660
H	-1.475519958500	-2.630410083593	3.987715720969
C	-2.833182594356	-3.055886572874	-0.936191000918
H	3.454292787877	-5.592976062292	-0.291542717095
C	-2.444061083416	-3.022944756514	1.441736253557
H	-3.420895818240	-3.331747431492	-1.802214446393
H	-2.749572957366	-3.282935603989	2.446065885956
C	-3.222604402746	-3.390005363381	0.354027359946
H	0.166943889191	-1.782401786509	5.627337024474
H	2.341910410281	0.597352473493	-5.852738610371
Cl	-4.706789391898	-4.282173504322	0.614866409755
Cl	-2.518846412514	4.634376472112	-1.948372079619
H	4.220099164883	-5.396820745197	-2.675754542623
H	5.745609355326	1.483783121876	2.796194842872

8.3.4 [Fe(5-Cl-terpy)$_2$]$^{2+}$ (**H1**)

Fe	0.937690448456	-0.869646525133	-0.233641290148
N	1.268089200770	-0.453539563719	-2.311871799638
N	2.085280059739	-2.622184075350	-1.048454324938
N	-0.266380138748	0.949344290616	-0.692799205637
N	0.706887556543	-1.250820189173	1.867578035108
N	-0.925337649121	-2.061115986628	-0.027256322855
N	2.692187562183	0.062994970132	0.752195434373
H	-2.213860954049	4.107536570989	-1.761806276239
H	5.541013154807	1.756960329677	0.187728083720
H	5.679306802594	1.556068269321	2.686560288771
H	-0.681428149497	2.985904869728	-3.349569925704
C	-1.667995732726	3.221853382665	-1.456743415891
C	4.769988729259	1.251550959069	0.756751694632
H	-2.484622879416	3.151506285825	0.546059997175
C	-0.806651031658	2.592449154988	-2.348928614089
C	4.841480663576	1.137924803588	2.139805858970
C	-1.820970874826	2.696473080673	-0.179262810158
H	0.709486808395	1.972920893906	-4.572519294846
H	3.583734441041	0.764158391265	-0.976472895973
C	3.675395706740	0.697361690504	0.102629029373
C	1.098655677743	1.060509237765	-4.140482567811
C	-0.120131762098	1.451729159349	-1.939696869668
C	3.824550787358	0.475504806520	2.820141202175
H	3.870777740651	0.372304391205	3.896978128414
C	-1.098630090805	1.558029910693	0.159624258206
C	1.934599328124	0.237637698707	-4.886676575298
C	0.777094861709	0.682197539631	-2.837129853961
H	-1.191685417675	1.118106198749	1.147191664856
C	2.755628335233	-0.050672711990	2.098099246164
C	2.426273090992	-0.939300060299	-4.334339713090
H	3.073053043458	-1.582923077914	-4.916061437240
H	2.197665658525	-0.566253856312	4.803983071303
C	2.064194673734	-1.266535371021	-3.028185000947
C	1.620988352709	-0.770848916338	2.729331686252
C	1.472227470681	-0.956710585892	4.102307288588
C	2.491468542974	-2.502639784807	-2.331825860393
H	3.563971098649	-3.406168362082	-3.977095433343

C	0.360329070640	-1.655527728343	4.563667146022
C	3.242545218385	-3.502004489007	-2.948387114321
C	-0.372173125760	-1.926567139058	2.299860340076
H	-1.376112419105	-2.145424344271	-2.049173045412
C	2.410149925596	-3.714310957822	-0.354230808597
C	-0.573308826165	-2.151304798369	3.662526669309
C	-1.698845801839	-2.426237827815	-1.052660516277
H	2.067032670983	-3.775882457782	0.672279820754
C	-1.289701637502	-2.386149628698	1.233821717778
C	3.584021543529	-4.642755513910	-2.234973915590
C	3.158199061042	-4.742822140275	-0.918417572385
H	-1.435531639774	-2.698515184411	4.020709482479
H	4.164749928691	-5.431916190384	-2.697645267298
C	-2.877314341434	-3.137067794753	-0.858784079415
C	-2.461163628429	-3.100426113887	1.485838137891
H	-2.751109079012	-3.360470884014	2.495557984799
C	-3.270433176094	-3.485530355162	0.426435699354
H	-4.184013324137	-4.040583025622	0.601511226409
Cl	3.546606342717	-6.153602928315	0.043928509816
Cl	-3.848337059053	-3.580220988481	-2.246873176047
H	2.205975859542	0.515468415773	-5.898650021767
H	0.222345581158	-1.814575354301	5.627323902207

8.3.5 [Fe(5,5''-diCl-terpy)$_2$]$^{2+}$ (H2)

Fe	0.915199222151	-0.887013030549	-0.241406893907
N	1.276236142470	-0.453405818218	-2.312371731941
N	2.097676360628	-2.611914363940	-1.037203741523
N	-0.262554333968	0.958773243080	-0.706406641206
N	0.703072955487	-1.269794137096	1.862954166132
N	-0.949602908767	-2.062776330701	-0.023629226302
N	2.660134190597	0.092430680051	0.750456535030
H	-2.135767502769	4.168006432170	-1.764681648801
H	5.667695034808	1.561390629050	2.677122971955
H	-0.621229857184	3.023743244404	-3.347905804529
C	-1.608424073622	3.269622710081	-1.467400324408
C	4.706499380870	1.303855746258	0.764528548634
C	-0.758326622497	2.620983363540	-2.352795245893
C	4.822541734001	1.149993601353	2.138102885285
C	-1.767404696247	2.730035403394	-0.199081219982
H	0.672160180329	1.932707505836	-4.605884808000
H	3.508381898810	0.867052624409	-0.975686190833
C	3.613013575037	0.759960994190	0.098251386527
C	1.073820500909	1.030733408556	-4.163720687656
C	-0.100241759323	1.460714867623	-1.950760549251
C	3.824223393822	0.454270099412	2.807140533109
H	3.897987845320	0.319153271415	3.878596195904
C	-1.078926487493	1.573063191717	0.151176016069
C	1.898859673895	0.197205065959	-4.909955292405
C	0.777622358504	0.674627724769	-2.848502753545
H	-1.191798546346	1.138009245362	1.138126081686
C	2.745773248325	-0.060441750739	2.090231907985
C	2.401950836261	-0.969184262886	-4.346078717327
H	3.039632091105	-1.621340411632	-4.928157569847
H	2.222113214207	-0.612789151395	4.791758402573
C	2.061763381381	-1.276644601017	-3.029381413835
C	1.624938403718	-0.797173164517	2.720703135326
C	1.491618234080	-0.998955939026	4.093051062551
C	2.499578521541	-2.502659671046	-2.322902955095
H	3.574063125192	-3.414693593253	-3.961523206006
C	0.386750458928	-1.705863433064	4.557424548507

C	3.254933514554	-3.503159528954	-2.931468536055
C	-0.370090300718	-1.953092615219	2.298893608919
H	-1.421648607428	-2.125600484804	-2.041586195291
C	2.430344194528	-3.695515967646	-0.333290587268
C	-0.555708395731	-2.193541415449	3.660577729800
C	-1.731731295433	-2.419536058849	-1.044919118297
H	2.091518755775	-3.748983362733	0.695010743610
C	-1.298519866942	-2.403927782028	1.237431902129
C	3.604349762016	-4.635373055938	-2.208294176073
C	3.181872743802	-4.725649717266	-0.889967777562
H	-1.413065261571	-2.746015263268	4.022050957832
H	4.188809145865	-5.425147436498	-2.665099515182
C	-2.904720026945	-3.138960329487	-0.846564547036
C	-2.463647151459	-3.125375839949	1.494071680679
H	-2.741763544687	-3.397695442994	2.503819964885
C	-3.282433565746	-3.502757669944	0.438668837064
H	-4.191467309626	-4.063910476329	0.617825180429
Cl	3.579474523321	-6.124288302416	0.085005240230
Cl	-3.886914614321	-3.571195862180	-2.229120204911
H	2.150845539169	0.457390873410	-5.931583144923
H	0.260370447924	-1.876971922612	5.620576290772
Cl	-2.836504145260	3.478271382359	0.967659993316
Cl	5.919010284753	2.176437885246	-0.146185082050

8.3.6 [Fe(6-Cl-terpy)$_2$]$^{2+}$

Fe	0.835478333408	-1.126675102958	-0.313745799705
N	1.342303291474	-0.450668804994	-2.298776855830
N	2.059684739194	-2.791390651923	-1.272030702922
N	-0.270217770696	0.795792517711	-0.623075181207
N	0.518881072035	-1.146104567474	1.819749810047
N	-1.047226986656	-2.364348582743	0.052268791433
N	2.632936554661	-0.134340587239	0.604471056942
H	-1.743157893916	4.311156794326	-1.255159845737
H	5.633106201494	1.251141465523	-0.026351446225
H	5.494630732533	1.706526099977	2.441153178282
H	-0.172974497842	3.301885712441	-2.880008099090
C	-1.334624285797	3.323425569273	-1.075137045913
C	4.775019391476	0.941209795982	0.558027741836
H	-2.374883635846	3.002361698453	0.795215585596
C	-0.452971001248	2.756276448810	-1.988228834462
C	4.694264506279	1.190741828564	1.922334483864
C	-1.685361886680	2.604121657352	0.060553796362
H	1.120125681572	2.321296021282	-4.191931094923
H	3.752520229439	0.068266409198	-1.120856820152
C	3.720178809958	0.277471958070	-0.057848981108
C	1.419726143732	1.330547156799	-3.877712151246
C	0.058183464656	1.484065764767	-1.738487391939
C	3.569478434399	0.764576908633	2.619774775094
H	3.494129157092	0.945849337449	3.684247069301
C	-1.127764435187	1.344138273829	0.244959976407
C	2.268757418640	0.575405942811	-4.676322683704
C	0.967948895041	0.784181880868	-2.675743318640
H	-1.380778656979	0.756870314921	1.120755429964
C	2.554157381481	0.100509024052	1.932748276747
C	2.644307741616	-0.701630228758	-4.276819606138
H	3.309803759384	-1.289040063500	-4.895366322580
H	1.695367487996	0.369655472162	4.581901243850
C	2.151489350753	-1.196378206715	-3.069901472228
C	1.341208468234	-0.425003775275	2.601687300332
C	1.045626683197	-0.220927741974	3.949705298329

C	2.488445030232	-2.536755419031	-2.535474448099
H	3.532080132766	-3.268762882096	-4.274904641292
C	-0.107613673535	-0.788197475107	4.475453090257
C	3.204334527146	-3.478834211673	-3.266007699736
C	-0.587892228931	-1.719047392920	2.322285177459
C	2.363551981983	-3.965580145858	-0.738490228855
C	-0.935519443886	-1.553941450261	3.662686430011
C	-1.754859239623	-3.026911033097	-0.850686487945
C	-1.393321610863	-2.503880177437	1.357760107832
C	3.497079221761	-4.708165593882	-2.683774594246
C	3.078443247229	-4.965906511087	-1.387733406484
H	-1.834396823959	-1.998630886934	4.068326552051
H	4.051926473048	-5.458433433017	-3.235433130285
C	-2.806473241883	-3.887005510161	-0.553838144008
H	3.294407322580	-5.903212094242	-0.892132011282
C	-2.431327943006	-3.341232090988	1.751819530413
H	-3.337296398137	-4.403972795277	-1.342212871734
H	-2.685936339638	-3.456314365581	2.797170134516
C	-3.139591505946	-4.045466996872	0.783162501775
H	-3.946830339205	-4.710066149585	1.069488713062
H	-0.364967141556	-0.629589870148	5.516567000104
H	2.642437297394	0.983065925914	-5.608407606590
Cl	1.832663009210	-4.253265835971	0.913133204048
Cl	-1.323161192075	-2.773973344388	-2.537048331607

8.3.7 $[Fe(4'\text{-}Cl\text{-}terpy)_2]^{2+}$

Fe	1.026493879677	-0.804851520698	-0.209626299736
N	1.328432725628	-0.352176879838	-2.284546092677
N	2.195746401389	-2.508968154402	-1.045728965977
N	-0.124667364745	1.064973832684	-0.607175673454
N	0.755350776899	-1.216499057137	1.878325691106
N	-0.853060169309	-1.964593589661	-0.059119923441
N	2.761743192786	0.115225625232	0.830434797365
H	-1.829971658208	4.412315430658	-1.505970980009
H	5.629737043015	1.801407671597	0.349590294689
H	5.740347763746	1.521228328202	2.841704317902
H	-0.484388731413	3.202042180912	-3.193799116982
C	-1.355027793439	3.472130843333	-1.250027173913
C	4.848527958423	1.284973805142	0.894326609914
H	-2.070561164272	3.423221228835	0.791524541510
C	-0.600981718474	2.790232687794	-2.199306548375
C	4.904677204174	1.128090999402	2.273668638142
C	-1.488996796002	2.929844084826	0.022228313464
H	0.507001438303	1.967536983101	-4.582345005961
H	3.675247358844	0.862325472212	-0.866130894206
C	3.756250605313	0.760937560013	0.211168447090
C	0.953170405520	1.083921297548	-4.147947715528
C	-0.002384214285	1.582423312848	-1.850165394592
C	3.874036906488	0.455186950390	2.923136814034
H	3.908212673056	0.318791756557	3.996775683178
C	-0.854842433774	1.723702439179	0.298963343315
C	1.712135168910	0.220069436836	-4.927156453721
C	0.785328384627	0.762000588250	-2.802809717932
H	-0.935536535006	1.269741326451	1.281269766999
C	2.810149777095	-0.038149905187	2.172480093187
C	2.280575687535	-0.927179481515	-4.390089814672
H	2.863775919092	-1.591786539187	-5.012289179460
H	2.201750682863	-0.602135220628	4.856505249309
C	2.056789679774	-1.190327153473	-3.041306170632
C	1.657625116202	-0.763229272446	2.765665263875
C	1.488987923522	-0.969532192189	4.131244056710
C	2.567025393433	-2.394468132289	-2.340693858375
H	3.655714617005	-3.252007734385	-3.997198105232
C	0.357425591798	-1.664836216432	4.542899541962
C	3.363145558283	-3.354776685423	-2.960091126672
C	-0.337906523291	-1.888362178136	2.276439274927
H	-1.271344807506	-2.006246277434	-2.082381983112
C	2.606085599520	-3.573684405286	-0.347241003139
C	-0.571867159788	-2.139517338872	3.627954179703
C	-1.617428311430	-2.314267532694	-1.101240588543
H	2.284543954212	-3.628296943270	0.687336199210
C	-1.242980969885	-2.320373793640	1.186045825677
C	3.785843156401	-4.458626973792	-2.227153227893
C	3.404123257617	-4.571885165843	-0.895941467402
H	-1.444690447714	-2.682487180507	3.963111892869
H	4.406592933346	-5.215599259701	-2.693366949976
C	-2.798289434992	-3.033106078743	-0.959465930145
H	3.713005623441	-5.413195881932	-0.287292693993
C	-2.417358391065	-3.038684317226	1.406686269989
H	-3.379421674086	-3.294810933588	-1.835501601733
H	-2.721631161951	-3.316004599046	2.407811932689
C	-3.202626373375	-3.401452382367	0.318655874359
H	-4.117214992186	-3.963191431822	0.470775100975
Cl	1.947683574108	0.575498872842	-6.621442116300
Cl	0.098983144155	-1.943113956057	6.249388109636

8.3.8 $[Fe(3'\text{-}Cl\text{-}terpy)_2]^{2+}$

Fe	1.006013737911	-0.742299235302	-0.203302011233
N	1.240965956959	-0.427112590910	-2.309252559842
N	2.212828372342	-2.456999646276	-0.895320697220
N	0.016322663880	1.177264403453	-0.631630934118
N	0.743051037234	-1.257113505851	1.860670738500
N	-0.860883666863	-1.905031809147	-0.148866809936
N	2.660869397016	0.226059733048	0.860881456620
H	-2.593006174837	3.805166295321	-1.698586178065
H	5.654483177555	1.631392120829	0.252336063599
H	6.025941259141	0.874206597773	2.618439053040
H	-1.255261775931	2.628315244951	-3.394917952851
C	-1.849650823917	3.078542435176	-1.389502708378
C	4.880646166466	1.119837424118	0.811707680007
H	-2.206932478795	3.298562017840	0.733524652945
C	-1.089430115588	2.417403140235	-2.349989989207
C	5.076973700773	0.706451568834	2.121294661691
C	-1.647250205117	2.796358733520	-0.045922459304
Cl	0.652174748516	2.497676231970	-4.992346791653
H	3.461164863921	1.135053717969	-0.811122091363
C	3.654985748796	0.846959589338	0.216656179301
C	1.103529379698	1.009961887093	-4.181762615326
C	-0.138352867213	1.479804803251	-1.945097832015
C	4.044351351607	0.066256460363	2.801061625079
H	4.203870943190	-0.274273795447	3.811813737464
C	-0.707092742211	1.827579287975	0.286113225083
C	1.913776048016	0.135563365276	-4.901379148292
C	0.736108957671	0.695776944035	-2.863224430123
H	-0.525923362560	1.560480562690	1.322046416078
C	2.824964382350	-0.141272151581	2.156539481468
C	2.382895418940	-1.020915097369	-4.306127853072
H	3.020002888153	-1.691331633506	-4.867292534134
Cl	2.322478510093	-0.316304920423	5.427876394643
C	2.038547072151	-1.273571688419	-2.979272482048

C	1.636565219746	-0.804245277610	2.765522540433
C	1.341734467891	-0.968970730768	4.128690077716
C	2.531059805033	-2.440756570607	-2.209979039998
H	3.536768651308	-3.445127485943	-3.837328673727
C	0.183849165191	-1.644436574519	4.508170954963
C	3.290432666142	-3.459592374641	-2.783546833729
C	-0.387395618695	-1.891883552250	2.205424120714
H	-1.238857344105	-1.892521325836	-2.179282453073
C	2.649751995637	-3.465063429577	-0.130103370703
C	-0.687228531357	-2.122966936620	3.546906235325
C	-1.609355397356	-2.218748750048	-1.213835767819
H	2.370144538661	-3.433475950910	0.917237919953
C	-1.267525257652	-2.290512978513	1.082027402512
C	3.736833440897	-4.505535880359	-1.983975311849
C	3.415895556013	-4.510336603974	-0.631249628794
H	-1.584480134171	-2.645353735426	3.850320417551
H	4.329106314863	-5.304535998189	-2.416088365178
C	-2.798299914388	-2.929288022561	-1.109822292085
H	3.747887225622	-5.302824226590	0.028463893291
C	-2.450264855568	-3.005974214456	1.262111726635
H	-3.367999874663	-3.163447555881	-2.000892989727
H	-2.771331964315	-3.311922366589	2.249200265516
C	-3.222359495973	-3.329117023773	0.152075085730
H	-4.143097973836	-3.888094339246	0.275125631260
H	-0.040449330011	-1.776713939094	5.560008416549
H	2.194759079739	0.377923357153	-5.918995684803

8.3.9 [Fe(4'-SMe-terpy)$_2$]$^{2+}$(**S0**)

See the Supplementary Information of Ref. 2.

8.3.10 [Fe(5-Cl-4'-SMe-terpy)$_2$]$^{2+}$ (**S1**)

Fe	0.906738725356	-0.910998862271	-0.245629193990
N	1.287561298510	-0.443903531446	-2.297436370681
N	2.064523599213	-2.645526704079	-1.087696168579
N	-0.291587579635	0.918510918079	-0.701750656156
N	0.700163023355	-1.240880751906	1.860175504627
N	-0.955626134554	-2.100600972405	0.015853863524
N	2.696514830878	0.017863968794	0.709965226597
H	-2.294523857254	4.034755107731	-1.791905723812
H	5.555899843686	1.680279826660	0.100373318013
H	5.680501260762	1.572342757352	2.606690216357
H	-0.669279897466	2.989311168683	-3.336483058939
C	-1.732034878248	3.162084772685	-1.479739765788
C	4.780241915644	1.198965547078	0.683830846453
H	-2.622508568360	3.029633639122	0.487823040203
C	-0.817769455733	2.575595676541	-2.347697545825
C	4.845315309740	1.135988759405	2.070315791261
C	-1.917084622077	2.608708164031	-0.218423042510
H	0.843991476782	2.089795683738	-4.455023652087
H	3.600260251204	0.656163521795	-1.035860476884
C	3.685947338359	0.625974045934	0.045190662578
C	1.221908827221	1.154786230870	-4.067461625832
C	-0.113342533362	1.448759649257	-1.932013051362
C	3.822855254023	0.500692367419	2.766889151760
H	3.862779314990	0.439935937969	3.846947666587
C	-1.172971030135	1.486892970030	0.128813199003
C	2.098441975162	0.366626384601	-4.821285682993
C	0.834250084993	0.713295324216	-2.807522741732
H	-1.288945032134	1.027369291898	1.104901605957
C	2.757275785915	-0.050457761915	2.058369766832
C	2.539090612044	-0.853556228016	-4.283975566100
H	3.210042575122	-1.484179735131	-4.853944232896
H	2.245654984248	-0.498552263333	4.746570055318
C	2.111851492482	-1.229079488775	-3.020100839502
C	1.620415042642	-0.751848667943	2.708417186668
C	1.495495632203	-0.905101813099	4.084394714979
C	2.506005160962	-2.494867106536	-2.355932175015
H	3.611794281724	-3.365696460594	-3.997665272355
C	0.382720896794	-1.584312106242	4.593676593702
C	3.262833352931	-3.485000158136	-2.980437571645
C	-0.377993749334	-1.899935959677	2.331911054636
H	-1.419382513631	-2.237751359072	-2.000085664343
C	2.359042344771	-3.760706645279	-0.416016732622
C	-0.567688425893	-2.090259865746	3.691811756439
C	-1.739492806485	-2.486130074219	-0.994058833008
H	1.987763937532	-3.847111731828	0.598988358563
C	-1.312792762783	-2.385498069000	1.288098306407
C	3.572676492825	-4.649067110329	-2.290801329642
C	3.111622363873	-4.780356496297	-0.988881105174
H	-1.433593073528	-2.624943626711	4.061839568874
H	4.156488195267	-5.432307169636	-2.759606825872
C	-2.924441556187	-3.177691395659	-0.771909049182
C	-2.487483612930	-3.083304143886	1.568212666304
H	-2.772355441869	-3.315968243798	2.586142136515
C	-3.309119065669	-3.488379195202	0.525397502402
H	-4.226011920744	-4.030427126235	0.722649666147
S	0.084185457018	-1.854995016608	6.310096205850
C	1.559707854911	-1.161545337347	7.137811110765
H	1.613118424582	-0.080382927648	7.010055444592
H	2.465639911610	-1.650349317561	6.780575717989
S	2.697739487114	0.801786442596	-6.421324801385
C	2.014257618067	2.472829479641	-6.719659106178
H	0.926559773711	2.447387812106	-6.785078429131
H	2.351433161094	3.170831876324	-5.953819453405
H	1.416305981478	-1.395034679729	8.193564038830
H	2.427832252969	2.768080943264	-7.684245946653
Cl	3.461430840926	-6.221994168478	-0.057051479127
Cl	-3.913148586681	-3.646378676045	-2.139240524322

8.3.11 [Fe(5,5''-diCl-4'-SMe-terpy)$_2$]$^{2+}$ (**S2**)

Fe	0.906685661774	-0.930892303424	-0.250228658416
N	1.314914512962	-0.437035560354	-2.288374919403
N	2.103951313949	-2.632529163390	-1.075223453479
N	-0.246470535276	0.941758704767	-0.687135953142
N	0.684646293555	-1.251552127856	1.852414474771
N	-0.966850492017	-2.095633887250	-0.003126519826
N	2.675260860485	0.040184125400	0.726359136637
H	-2.145084224841	4.141845482811	-1.728676694173
H	5.657625888312	1.568467481411	2.651234671693
H	-0.575924398362	3.053294887917	-3.294711917610
C	-1.609830448103	3.246942006502	-1.435223294120
C	4.734768962971	1.231465957876	0.731025651907
C	-0.727871380229	2.629443307616	-2.310987552503
C	4.821123050908	1.139658347366	2.112409263402
C	-1.790752124849	2.679103348049	-0.182340349625
H	0.805863016881	2.056440862622	-4.478889636629
H	3.565956536134	0.734785576653	-1.011665653286
C	3.648356450251	0.671043748098	0.067498592294
C	1.194966514148	1.128742700917	-4.085676511975
C	-0.061033676088	1.472282709809	-1.915525610441

C	3.802237235536	0.482906062527	2.788603075155
H	3.850754991053	0.400601422916	3.866583876485
C	-1.092441264022	1.526486466122	0.162134435563
C	2.044703065816	0.325016982695	-4.853366459312
C	0.849896594562	0.713225977942	-2.805122682771
H	-1.221116611383	1.070719592021	1.137692090740
C	2.736297687951	-0.058130539993	2.072159619896
C	2.505715667128	-0.882835280424	-4.304672088903
H	3.158518216311	-1.524045903216	-4.883587297095
H	2.219680303254	-0.529965676227	4.750665351036
C	2.119434766822	-1.234358282374	-3.021458648313
C	1.601297209590	-0.768585714396	2.708817473135
C	1.471437484974	-0.931030120040	4.082996570322
C	2.536082557899	-2.487742260350	-2.347448265482
H	3.650736907469	-3.356273801184	-3.983773210737
C	0.356578366447	-1.613163405099	4.583362362055
C	3.306092622081	-3.471670990115	-2.964660425285
C	-0.394897155284	-1.915297803629	2.316214306071
H	-1.423869945692	-2.214746264461	-2.022004011863
C	2.418564811552	-3.735924861209	-0.393224291174
C	-0.589657686254	-2.114483721142	3.673309657811
C	-1.743990004921	-2.476938074629	-1.019582169715
H	2.054639210049	-3.816965802488	0.624832891926
C	-1.324964777217	-2.395864256502	1.265295396617
C	3.637075980268	-4.623760724895	-2.264734592807
C	3.183676642283	-4.749942283409	-0.959581186432
H	-1.456488044202	-2.652426381645	4.036182828440
H	4.232265395512	-5.401465044440	-2.728383687747
C	-2.922945831706	-3.182377476455	-0.807730007996
C	-2.495461468634	-3.103370944271	1.535196515150
H	-2.783084703432	-3.345465808433	2.550077351568
C	-3.309573495164	-3.506460771078	0.485448766127
H	-4.222845850684	-4.057336489684	0.674848459019
S	0.050589224288	-1.891962801614	6.295340774712
C	1.513133228410	-1.184718731656	7.134139791991
H	1.555864727425	-0.102978802235	7.007213265026
H	2.426601123194	-1.664098836537	6.783442935684
S	2.581630287779	0.724311092469	-6.482761034500
C	1.874463252297	2.382873286752	-6.796115887106
H	0.785502816806	2.346762292602	-6.829004364683
H	2.228198691659	3.100018411836	-6.055972105008
H	1.364177802992	-1.420449505821	8.188587938088
H	2.256755361760	2.660934019074	-7.778542787365
Cl	3.560366171700	-6.175155177620	-0.014595758326
Cl	-3.901324712016	-3.648370256709	-2.182261011256
Cl	-2.900721917239	3.386779517248	0.971351650514
Cl	5.977083210417	2.047222974237	-0.192851235331

8.4 Quintet-singlet MECP

8.4.1 [Fe(terpy)$_2$]$^{2+}$(**H0**)

See the Supplementary Information of Ref. 2.

8.4.2 [Fe(3-Cl-terpy)$_2$]$^{2+}$

Fe	0.933679809993	-0.886092169820	-0.240576486645
N	1.182731163011	-0.501306536734	-2.240790711453
N	2.145599643037	-2.535544903030	-0.934552305446
N	-0.200555039037	0.936537154665	-0.521350996652
N	0.696413259153	-1.239383421083	1.763127453746
N	-0.887660127157	-2.023634397228	-0.126073896664
N	2.673061904306	0.061561036011	0.608125864164
H	-2.245162727532	4.066653984662	-1.486829551132
H	5.530555853852	1.726441654837	0.002745253115
H	5.681675934362	1.564316336903	2.503956595505
H	-0.859627531430	2.907236448746	-3.179645306274
C	-1.668383060736	3.191208630610	-1.210514746655
C	4.757787066378	1.237666841945	0.583879159759
H	-2.287636813836	3.177833057558	0.863582934503
C	-0.890036703242	2.540701586022	-2.161313332522
C	4.836045760340	1.146454272912	1.969137143367
C	-1.696706890034	2.702112539126	0.090426895648
H	0.613119976188	1.993267545534	-4.426544343611
H	3.566727148497	0.722088456772	-1.136110552685
C	3.658873291076	0.678492207649	-0.056286231107
C	1.005894937038	1.068691306927	-4.023887056932
C	-0.161835909196	1.412756239199	-1.789365199472
C	3.816971363065	0.506864995603	2.665265932138
H	3.867942059363	0.421257034629	3.743416303332
C	-0.946002430607	1.572117376588	0.390230100777
C	1.861936545478	0.281876699302	-4.780995719165
C	0.690837851360	0.650852902455	-2.731290031778
H	-0.945445203889	1.157688375279	1.392462773388
C	2.737754208817	-0.022780289865	1.958964742920
C	2.365472239029	-0.907159828405	-4.261242619216
H	3.044322575696	-1.500482605035	-4.851815350598
H	2.120537232636	-0.335512945812	4.676332771908
C	1.981669689799	-1.303451737329	-2.979240565503
C	1.593549597637	-0.700354607367	2.609897421269
C	1.404228176955	-0.767301324346	3.989657338354
C	2.398721251477	-2.547465000276	-2.271236980879
C	0.254650935228	-1.378329604175	4.467725485545
C	2.984681129398	-3.695440074222	-2.829719473888
C	-0.411371618449	-1.874000004826	2.211134386203
H	-1.186450208156	-2.042118509328	-2.165805655396
C	2.496510889645	-3.558723699976	-0.153297194702
C	-0.666247280971	-1.931486617386	3.582691007865
C	-1.550335882534	-2.416208931540	-1.215834781169
H	2.265517020536	-3.465605740751	0.901714787623
C	-1.263192447544	-2.424733406270	1.118398730624
C	3.357815506796	-4.760701900334	-2.014163398351
C	3.123839287524	-4.691740075922	-0.652313515066
H	-1.561594539708	-2.388164901160	3.969015879648
H	3.817936182466	-5.636912557277	-2.455210447219
C	-2.652456290006	-3.256022749836	-1.151158058942
H	3.408611911262	-5.501973098065	0.007482873864
C	-2.357385011314	-3.298989932355	1.229268259060
H	-3.173767806827	-3.554959182001	-2.052033351785
C	-3.053142554271	-3.707147394708	0.093393700982
H	-3.896030716500	-4.380536445537	0.195588954926
H	0.063246770920	-1.419929115894	5.534057950778
H	2.153972608139	0.598725473801	-5.775411738996
Cl	3.248453211586	-3.925294029763	-4.552231063428
Cl	-2.922919439067	-3.980006525080	2.747400761323

8.4.3 [Fe(4-Cl-terpy)$_2$]$^{2+}$

Fe	1.021777000000	-0.841308000000	-0.218258000000
N	1.325658000000	-0.437385000000	-2.201856000000
N	2.214513000000	-2.519769000000	-0.882175000000
N	-0.092751000000	1.002865000000	-0.537209000000
N	0.727871000000	-1.213400000000	1.772001000000
N	-0.845017000000	-1.932414000000	-0.185252000000
N	2.746596000000	0.070001000000	0.712527000000
H	5.612364000000	1.756890000000	0.218691000000
H	-0.742168000000	2.927685000000	-3.237187000000

C	-1.551273000000	3.232443000000	-1.269568000000
C	4.830501000000	1.246330000000	0.768111000000
H	-2.187206000000	3.274962000000	0.801210000000
C	-0.773595000000	2.577619000000	-2.214111000000
C	4.885666000000	1.103064000000	2.150036000000
C	-1.591447000000	2.782659000000	0.043429000000
H	0.627008000000	1.902464000000	-4.516929000000
H	3.666026000000	0.797740000000	-0.989846000000
C	3.742178000000	0.712372000000	0.088808000000
C	1.049715000000	1.011360000000	-4.071145000000
C	-0.050461000000	1.457331000000	-1.812971000000
C	3.855491000000	0.437158000000	2.805009000000
H	3.885775000000	0.312545000000	3.880301000000
C	-0.839847000000	1.659102000000	0.357961000000
C	1.870700000000	0.176872000000	-4.823866000000
C	0.794003000000	0.674056000000	-2.743563000000
H	-0.846993000000	1.268053000000	1.369365000000
C	2.790276000000	-0.067178000000	2.061315000000
C	2.408099000000	-0.971486000000	-4.252662000000
H	3.044278000000	-1.622708000000	-4.838134000000
H	2.169806000000	-0.631770000000	4.754426000000
C	2.105998000000	-1.263945000000	-2.922981000000
C	1.637439000000	-0.775219000000	2.663077000000
C	1.448603000000	-0.985407000000	4.028773000000
C	2.569066000000	-2.463970000000	-2.190123000000
H	3.572656000000	-3.437798000000	-3.833212000000
C	0.304021000000	-1.655227000000	4.450326000000
C	3.295518000000	-3.491568000000	-2.788125000000
C	-0.382231000000	-1.864482000000	2.166433000000
H	-1.215522000000	-1.984223000000	-2.219061000000
C	2.573707000000	-3.585046000000	-0.155214000000
C	-0.625567000000	-2.103446000000	3.517315000000
C	-1.583922000000	-2.284092000000	-1.244720000000
H	2.273446000000	-3.583064000000	0.886730000000
C	-1.264881000000	-2.282424000000	1.054682000000
C	3.659680000000	-4.597010000000	-2.027802000000
C	3.293058000000	-4.648402000000	-0.687548000000
H	-1.514288000000	-2.626900000000	3.845711000000
C	-2.771150000000	-2.993781000000	-1.137227000000
H	3.559042000000	-5.489656000000	-0.058836000000
C	-2.446447000000	-2.992459000000	1.252284000000
H	-3.335821000000	-3.258103000000	-2.021945000000
H	-2.777562000000	-3.266834000000	2.244791000000
C	-3.195283000000	-3.343370000000	0.137821000000
H	0.134799000000	-1.828911000000	5.506984000000
H	2.095454000000	0.425043000000	-5.854780000000
Cl	-4.685972000000	-4.236359000000	0.345292000000
Cl	-2.492788000000	4.629824000000	-1.744211000000
H	4.221657000000	-5.406591000000	-2.480271000000
H	5.721006000000	1.502443000000	2.714297000000

8.4.4 $[Fe(5\text{-}Cl\text{-}terpy)_2]^{2+}$ (H1)

Fe	0.950456595625	-0.876234442071	-0.232312025947
N	1.233972242700	-0.489155087290	-2.225622233331
N	2.100393329007	-2.598432213212	-0.924168780054
N	-0.179749645140	0.948735251843	-0.548881867289
N	0.703773427650	-1.229570460079	1.764518101604
N	-0.870864143494	-2.015488021752	-0.155045049685
N	2.674547533976	0.071240936948	0.658808994193
H	-2.188986876190	4.090893976829	-1.559278591028
H	5.505933972534	1.788156910514	0.081222132019
H	5.640390227270	1.621275728385	2.582479464468
H	-0.771785771605	2.923265066274	-3.222038684453
C	-1.624171128156	3.211508175552	-1.271204276301
C	4.738938063146	1.281791120771	0.654735047092
H	-2.292454580427	3.196737109337	0.786699232758
C	-0.828662214607	2.556697340251	-2.204805262452
C	4.808412543036	1.186774676561	2.039699821342
C	-1.685250321155	2.719618180537	0.027114866048
H	0.607862094480	1.904195071599	-4.501905245706
H	3.563754820942	0.761528979987	-1.075649286697
C	3.652914525753	0.708600968280	0.003958564934
C	1.008048855495	0.997070489652	-4.068286867243
C	-0.117342591341	1.423936183595	-1.817528602843
C	3.797321936331	0.523016081064	2.726103873891
H	3.839425327844	0.434736844918	3.804590197763
C	-0.945083770117	1.586473619898	0.343996556344
C	1.820121176497	0.160030716230	-4.826294867896
C	0.728148767323	0.642036640061	-2.748968992758
H	-0.966775979895	1.173108635331	1.346461994055
C	2.735644047202	-0.025146923463	2.009802859769
C	2.332251104959	-1.007965558121	-4.270870936414
H	2.963580827279	-1.659135770621	-4.861409108883
H	2.156874396052	-0.545732875018	4.720199957878
C	2.011100177752	-1.315030072286	-2.949956845997
C	1.607162864242	-0.747206575053	2.639988076105
C	1.440140302065	-0.937018185184	4.009704141288
C	2.457499003655	-2.527855352488	-2.229416411814
H	3.459357917772	-3.495197109071	-3.880539382387
C	0.323702967461	-1.639874345401	4.456593548701
C	3.176740477870	-3.554590973099	-2.837528389699
C	-0.379745511271	-1.910950312338	2.183042094022
H	-1.265393304975	-2.081931488825	-2.189157828831
C	2.443388337179	-3.668993734189	-0.205590520649
C	-0.597212426500	-2.136740439243	3.542473698133
C	-1.609178421682	-2.383432174693	-1.206688871613
H	2.142199659264	-3.689120703390	0.835073761135
C	-1.263931244685	-2.366860854728	1.094979066955
C	3.533859661051	-4.673947445156	-2.098615578543
C	3.156046921174	-4.725143789189	-0.764507046781
H	-1.463559500357	-2.686491607241	3.887179455323
H	4.089313425147	-5.484422533683	-2.555338285352
C	-2.775051267493	-3.122306342804	-1.052671690599
C	-2.428095294956	-3.106084318189	1.302953717361
H	-2.739137695401	-3.383645015644	2.301785615153
C	-3.198347019799	-3.493931722354	0.216834670209
H	-4.105057298432	-4.069405073249	0.358123394096
Cl	3.563252999423	-6.107679681404	0.229035170532
Cl	-3.695545206710	-3.572347378999	-2.471654221765
H	2.060330212380	0.421467939459	-5.850389678248
H	0.172426732853	-1.800587674352	5.517977958089

8.4.5 $[Fe(5,5\text{"-}diCl\text{-}terpy)_2]^{2+}$ (H2)

Fe	0.939310229078	-0.888061007134	-0.241082517416
N	1.245817444621	-0.484363857604	-2.222873723625
N	2.089225527414	-2.599495034943	-0.926032860958
N	-0.150797638195	0.966962289321	-0.550275234454
N	0.693814008577	-1.240459889162	1.757933198280
N	-0.878787400525	-2.056101859903	-0.161627816666
N	2.641364089946	0.110325251196	0.651566517997
H	-2.117551428139	4.140175340848	-1.547370165971
H	5.617279789802	1.640338059840	2.582137520012
H	-0.686243566022	2.985399610424	-3.199857951433
C	-1.564207436746	3.251760243975	-1.267819867710

C	4.691897946350	1.315296258910	0.661766377999
C	-0.759881172470	2.598919777399	-2.191332588027
C	4.781444817722	1.210611976686	2.042885346324
C	-1.643918880016	2.730259106488	0.016137922741
H	0.632681527467	1.910123639271	-4.505354251858
H	3.521876206507	0.828594014010	-1.083219016243
C	3.609089261495	0.754161188613	-0.005397796737
C	1.021614456805	0.997151567422	-4.073539485973
C	-0.069183807843	1.449211664517	-1.814823345631
C	3.771041173892	0.539875049650	2.717327851651
H	3.823928512230	0.441867501563	3.794196047542
C	-0.919090378248	1.590225843876	0.345368326254
C	1.801758546910	0.139158801979	-4.841392135394
C	0.755539530318	0.653946178860	-2.748275256377
H	-0.958158781913	1.175670084445	1.346044068275
C	2.705016573668	-0.002369407834	2.001188581989
C	2.296025655392	-1.036800863128	-4.286884499694
H	2.901220349191	-1.706331918229	-4.884292118587
H	2.132296738473	-0.516209238085	4.711720713562
C	1.994738144693	-1.328170685951	-2.957686413282
C	1.588429388307	-0.738214070534	2.630080341040
C	1.426169252464	-0.927308200739	4.001873398133
C	2.440115387963	-2.537572064115	-2.234092978595
H	3.442855372358	-3.514252642024	-3.878711955554
C	0.328903630707	-1.656808679059	4.449274698055
C	3.164477667846	-3.565620434287	-2.834130650836
C	-0.373439524995	-1.945449216959	2.177811436782
H	-1.288699655712	-2.109669611904	-2.191907494234
C	2.448523359290	-3.657797662461	-0.195972395844
C	-0.581551406690	-2.178315031344	3.536307574729
C	-1.626488067285	-2.413804613799	-1.208163934093
H	2.153169289820	-3.669378098916	0.846423177263
C	-1.259284281591	-2.410714325361	1.090527295988
C	3.534179657752	-4.673784788948	-2.085315102291
C	3.166239764611	-4.713689889272	-0.747404566601
H	-1.432671058651	-2.749406797818	3.884030395220
H	4.093669361150	-5.484568547534	-2.536634665365
C	-2.793401860087	-3.151643688161	-1.047693180287
C	-2.419231594779	-3.152422939566	1.305772894848
H	-2.721293078007	-3.436491011692	2.305490686051
C	-3.200380786733	-3.532700172943	0.223708916717
H	-4.104979127763	-4.110051805226	0.371446992224
Cl	3.591250026759	-6.081044736897	0.258600515814
Cl	-3.728751883936	-3.589981894790	-2.459434721500
H	2.028675070280	0.389081932894	-5.871343906245
H	0.182378374755	-1.819797136327	5.510972628834
Cl	-2.650833388386	3.485636524215	1.232203240818
Cl	5.925156028121	2.151188949250	-0.255152663663

8.4.6 [Fe(6-Cl-terpy)$_2$]$^{2+}$

Fe	0.884355	-0.992754	-0.274323
N	1.298224	-0.493725	-2.192322
N	2.069468	-2.735895	-1.021652
N	-0.170505	0.842303	-0.496281
N	0.565594	-1.153825	1.716992
N	-0.982252	-2.214217	-0.139677
N	2.591188	-0.007195	0.531116
H	-1.808620	4.259451	-1.250707
H	5.515971	1.521526	-0.115559
H	5.498031	1.740121	2.387213
H	-0.361539	3.165840	-2.937299
C	-1.354013	3.298856	-1.037562
C	4.698985	1.130831	0.479022
H	-2.203370	3.099059	0.943446
C	-0.543069	2.685283	-1.984308
C	4.685094	1.249021	1.864117
C	-1.574345	2.661208	0.177959
H	0.874832	2.098374	-4.295492
H	3.608649	0.395767	-1.223884
C	3.631272	0.498785	-0.145617
C	1.219705	1.143160	-3.921734
C	0.030701	1.449906	-1.692990
C	3.612210	0.725159	2.574935
H	3.586904	0.803947	3.654550
C	-0.959960	1.436972	0.407195
C	2.024260	0.332952	-4.713887
C	0.871203	0.700152	-2.646174
H	-1.103442	0.917537	1.347201
C	2.577926	0.095874	1.884226
C	2.463869	-0.893537	-4.228953
H	3.098050	-1.520863	-4.841236
H	1.849015	0.021520	4.596956
C	2.074427	-1.288415	-2.949586
C	1.414887	-0.521713	2.549953
C	1.175170	-0.491856	3.923437
C	2.467884	-2.563414	-2.315127
H	3.508110	-3.377623	-4.016622
C	0.046699	-1.133259	4.419251
C	3.204404	-3.530734	-2.990127
C	-0.524071	-1.787353	2.185598
C	2.424856	-3.865868	-0.418509
C	-0.813584	-1.793959	3.549636
C	-1.723794	-2.765852	-1.094960
C	-1.353938	-2.440908	1.153530
C	3.549341	-4.705951	-2.333654
C	3.159405	-4.884509	-1.016527
H	-1.692195	-2.294515	3.933893
H	4.120208	-5.473880	-2.843110
C	-2.832196	-3.574273	-0.866990
H	3.411048	-5.778830	-0.462001
C	-2.446423	-3.240379	1.472636
H	-3.384837	-3.993849	-1.696902
H	-2.717521	-3.421852	2.504084
C	-3.189356	-3.817286	0.449652
H	-4.040912	-4.448088	0.677526
H	-0.166087	-1.115811	5.482130
H	2.317499	0.660523	-5.704366
Cl	1.942292	-4.093935	1.253850
Cl	-1.280169	-2.451119	-2.764205

8.4.7 [Fe(4'-Cl-terpy)$_2$]$^{2+}$

Fe	1.036711649600	-0.796409898111	-0.208507505742
N	1.305770139815	-0.376237484266	-2.190180795644
N	2.244724173303	-2.455914756908	-0.907945890332
N	-0.063273849829	1.056805128949	-0.483830062669
N	0.763792839353	-1.202859922754	1.775250959127
N	-0.823373339007	-1.901858756135	-0.178096822777
N	2.770265323153	0.105410794216	0.726327261228
H	-1.947444585707	4.308070045739	-1.377392513530
H	5.624321931856	1.814426862962	0.243823090666
H	5.749798630144	1.520985134933	2.733764679420
H	-0.685688283507	3.078638579517	-3.114958557346
C	-1.416795254494	3.397776398373	-1.121922524740
C	4.848734257859	1.290724326421	0.789356261257

H	-1.968811395528	3.413309266867	0.970577136721
C	-0.709460257147	2.705867057407	-2.098751422417
C	4.913331227927	1.126310108061	2.168130377333
C	-1.431231099225	2.906656140363	0.178243392771
H	0.420755446461	1.902094628918	-4.506961305844
H	3.676245947892	0.863439401111	-0.969194242977
C	3.759116937729	0.761813445129	0.107407909411
C	0.884331428100	1.029600398695	-4.067304182746
C	-0.041401204132	1.533788297872	-1.752749184911
C	3.890564164378	0.444580694627	2.818544712280
H	3.929093238928	0.303161173102	3.891276556360
C	-0.739797684648	1.732608084995	0.451964286687
C	1.655818869095	0.172433326067	-4.843004111379
C	0.733521717630	0.721687546461	-2.717421344989
H	-0.731488709286	1.315179676952	1.452827034135
C	2.824105879604	-0.051742017957	2.072186511253
C	2.254151167430	-0.955772788004	-4.296648438412
H	2.850204177941	-1.613459960906	-4.914378859166
H	2.220162504707	-0.646474381842	4.759984643159
C	2.045916725336	-1.209859255229	-2.943940853173
C	1.675247464705	-0.772231714904	2.667165092534
C	1.499837097783	-0.991603961915	4.031010572389
C	2.570987149929	-2.390487025700	-2.222488116847
H	3.587061226122	-3.321630611124	-3.883415668951
C	0.353950896555	-1.666035728554	4.435209942285
C	3.328074191513	-3.386616587703	-2.834220057152
C	-0.344194520860	-1.858196752265	2.166746730846
H	-1.194792243301	-1.930614511732	-2.209891843328
C	2.659284071335	-3.506107872891	-0.188670491486
C	-0.586042805672	-2.112796316892	3.514228105590
C	-1.562621407903	-2.245875220059	-1.239644876992
H	2.378813512287	-3.516508134132	0.858776397381
C	-1.237421345923	-2.267796109794	1.060048210397
C	3.750710137546	-4.475946858416	-2.080935370694
C	3.410349465961	-4.540447268605	-0.734600406014
H	-1.471382061936	-2.639285281899	3.843705355664
H	4.338468897991	-5.260986079987	-2.543119416492
C	-2.745800146621	-2.965630167827	-1.126322505568
H	3.720974501218	-5.369884827802	-0.110821753609
C	-2.417473921004	-2.983580022725	1.249120810356
H	-3.308212754420	-3.222738560985	-2.015903553980
H	-2.742974337868	-3.266730458164	2.242247497669
C	-3.179175873443	-3.337228334485	0.141010689512
H	-4.098501827840	-3.897303429146	0.269786464295
Cl	1.874485655191	0.515947327006	-6.543772016787
Cl	0.086764512922	-1.955064434923	6.139838365969

8.4.8 [Fe(3'-Cl-terpy)$_2$]$^{2+}$

Fe	1.017653	-0.792487	-0.208276
N	1.296641	-0.404889	-2.218163
N	2.125644	-2.507990	-0.865142
N	-0.022868	1.054641	-0.514910
N	0.740137	-1.177861	1.799879
N	-0.787219	-1.926702	-0.203520
N	2.703879	0.125398	0.712744
H	-2.505226	3.877687	-1.479076
H	5.678954	1.591030	0.028070
H	5.959609	1.221072	2.511938
H	-1.080148	2.830844	-3.210784
C	-1.799260	3.094214	-1.198012
C	4.894043	1.126859	0.625422
H	-2.291542	3.115648	0.922008
C	-0.989749	2.505159	-2.178760
C	5.042545	0.924344	2.000418
C	-1.690023	2.670285	0.129946
Cl	0.745102	2.660512	-4.876632
H	3.551098	0.831807	-1.055833
C	3.708743	0.705260	0.016174
C	1.184967	1.085387	-4.061565
C	-0.090175	1.490906	-1.818551
C	4.001147	0.333415	2.728855
H	4.126345	0.160298	3.793225
C	-0.790602	1.642774	0.430675
C	1.988826	0.222044	-4.811296
C	0.801252	0.757431	-2.749413
H	-0.678364	1.270552	1.449916
C	2.821592	-0.048506	2.073463
C	2.428158	-0.977409	-4.250603
H	3.045216	-1.654194	-4.840069
Cl	2.343256	-0.149933	5.431565
C	2.077578	-1.267128	-2.925727
C	1.640222	-0.682607	2.709672
C	1.328963	-0.834937	4.073226
C	2.498645	-2.471199	-2.184910
H	3.516516	-3.491556	-3.809091
C	0.167693	-1.502063	4.476513
C	3.221301	-3.524186	-2.760633
C	-0.396279	-1.834681	2.164735
H	-1.086799	-1.981698	-2.265621
C	2.467000	-3.577074	-0.106671
C	-0.702359	-2.020718	3.518387
C	-1.486673	-2.299588	-1.302051
H	2.145209	-3.558258	0.935157
C	-1.232680	-2.293449	1.040796
C	3.563475	-4.631535	-1.974962
C	3.183297	-4.659589	-0.627125
H	-1.605057	-2.540935	3.834677
H	4.118653	-5.462313	-2.413681
C	-2.656695	-3.058426	-1.211890
H	3.435160	-5.503715	0.015119
C	-2.402075	-3.049789	1.193460
H	-3.189400	-3.348485	-2.117576
H	-2.755817	-3.344235	2.181075
C	-3.118869	-3.437630	0.054962
H	-4.030177	-4.028361	0.159903
H	-0.060269	-1.610101	5.537276
H	2.279109	0.491588	-5.826486

8.4.9 [Fe(4'-SMe-terpy)$_2$]$^{2+}$(**S0**)

See the Supplementary Information of Ref. 2.

8.4.10 [Fe(5-Cl-4'-SMe-terpy)$_2$]$^{2+}$ (**S1**)

Fe	0.967852905021	-0.883225619840	-0.233673819487
N	1.269927770798	-0.463615221285	-2.207328453754
N	2.103243956909	-2.591736701576	-0.947353267044
N	-0.149985326142	0.945116556680	-0.534452562494
N	0.724898392361	-1.230950028874	1.760760197653
N	-0.848890096799	-2.028958126884	-0.148845829380
N	2.698595759058	0.058436425053	0.656533522054
H	-2.111007547005	4.134027273782	-1.495018022046
H	5.546681856693	1.749494318190	0.080010627868
H	5.663093811493	1.613043126556	2.584599577152
H	-0.665139730224	3.004410568140	-3.158332220771

C	-1.560691486679	3.241233703104	-1.220679584583
C	4.772462760099	1.255296833594	0.654534025546
H	-2.278818001480	3.167350701317	0.818784927116
C	-0.749400601692	2.607194184821	-2.154843060797
C	4.832856584419	1.176977793189	2.040475091540
C	-1.656746451211	2.709282923195	0.059479351572
H	0.709450357693	2.005919965764	-4.408984406469
H	3.605147414686	0.723488698046	-1.077861766802
C	3.686238699521	0.681586608873	0.002835838942
C	1.103144579682	1.079233849144	-4.016588352176
C	-0.057320343964	1.457404199286	-1.786131731572
C	3.812505992022	0.526311035362	2.726252298225
H	3.847748948475	0.450842000895	3.805892961523
C	-0.932007058301	1.561414612692	0.359174123584
C	1.921189987000	0.255490126431	-4.798917553801
C	0.797592672755	0.686404189094	-2.718581457033
H	-0.980418320299	1.119247812046	1.348272787385
C	2.753939494066	-0.023892379700	2.008337385828
C	2.394746519710	-0.947856600584	-4.249404249501
H	3.028488354535	-1.599979053751	-4.837811642837
H	2.172197735797	-0.489076724278	4.695903510129
C	2.047725603493	-1.278732928435	-2.949607594081
C	1.617182007851	-0.738166187986	2.638101378660
C	1.449415024496	-0.902110361499	4.007369316663
C	2.475093838268	-2.510089560864	-2.248286382775
H	3.486334488225	-3.466498546283	-3.900149484600
C	0.327269446237	-1.598148130645	4.474857666049
C	3.192550774992	-3.535382329004	-2.860688024659
C	-0.363680496291	-1.902488671564	2.189829674869
H	-1.235766270937	-2.111321938290	-2.183178944501
C	2.431778552670	-3.673954856429	-0.237695050853
C	-0.591595816346	-2.105878511929	3.542010685664
C	-1.581926993484	-2.407914930945	-1.199894683221
H	2.119207856799	-3.704562811110	0.799428238653
C	-1.245682422839	-2.374340953481	1.101612006691
C	3.534512867581	-4.665597766948	-2.131133247118
C	3.143288318057	-4.727888384409	-0.801119079562
H	-1.464456550077	-2.650163671930	3.881007033952
H	4.088731340823	-5.475308684823	-2.590737418433
C	-2.745377294654	-3.151486563016	-1.044875192815
C	-2.405828323271	-3.118073344085	1.311388256061
H	-2.719115400791	-3.392209070638	2.310595294400
C	-3.171181562393	-3.517328719577	0.224916751488
H	-4.075096759463	-4.097081512045	0.366884963207
S	-0.022968031412	-1.884779300290	6.179936752110
C	1.409767687792	-1.167601718950	7.059874635126
H	1.449314830783	-0.085430729190	6.935271134922
H	2.336574353030	-1.640578302947	6.736166355359
S	2.410255552046	0.628377497752	-6.452130502684
C	1.746001914838	2.307688761515	-6.750083960034
H	0.656036118911	2.306271038286	-6.753919349372
H	2.140874770574	3.013479871068	-6.019955490602
H	1.231794868829	-1.404395103125	8.109641849121
H	2.111229606124	2.573300043619	-7.742464609405
Cl	3.532053895710	-6.124870249351	0.181570915896
Cl	-3.658196245161	-3.616483100937	-2.464993889745

8.4.11 $[Fe(5,5''\text{-diCl-4'-SMe-terpy})_2]^{2+}$ (S2)

Fe	0.953432527646	-0.891519184521	-0.237370435074
N	1.282376734169	-0.457482457447	-2.202584012800
N	2.113449461307	-2.579419160062	-0.937352806630
N	-0.151539937587	0.944702257926	-0.541439135894
N	0.712582125279	-1.233542857357	1.757751094983
N	-0.869827619354	-2.030948878043	-0.144436698395
N	2.673671322062	0.070992336584	0.647145865389
H	-2.109036028196	4.138274503569	-1.506744761924
H	5.665185049763	1.595684952239	2.566770236494
H	-0.640090078335	3.024448301757	-3.153837995702
C	-1.557804133924	3.246094428973	-1.235622254142
C	4.732322224778	1.264962879628	0.650177636503
C	-0.732492024603	2.615336021102	-2.156017138354
C	4.826078816763	1.168523148593	2.030819502165
C	-1.660325267029	2.693629392779	0.032969800606
H	0.730943333421	2.011429587139	-4.408065769159
H	3.559697262990	0.774180019886	-1.090859843953
C	3.644492363154	0.706418797436	-0.012532541448
C	1.120725357759	1.083694162365	-4.014693988534
C	-0.044464350702	1.461853817378	-1.789922163442
C	3.810946711080	0.508115839658	2.709010914942
H	3.862810024699	0.418820094197	3.786530700311
C	-0.941323388935	1.545999807673	0.350575587914
C	1.937684473417	0.257750622262	-4.795589179698
C	0.812372513327	0.692540420634	-2.717060652365
H	-1.006446565173	1.106333997172	1.339060662159
C	2.742559202556	-0.031500579806	1.996984314897
C	2.410734060047	-0.944585597593	-4.242169841851
H	3.044987354640	-1.598159370067	-4.828362519116
H	2.186663474935	-0.508277843094	4.684319730090
C	2.062719891633	-1.273129848097	-2.942629230601
C	1.612923623631	-0.746476197437	2.629893550621
C	1.455881385177	-0.914597665516	4.000258636191
C	2.489470842212	-2.502130153032	-2.237485380660
H	3.508025496101	-3.461968399407	-3.882051388496
C	0.334012430054	-1.605882665296	4.473627969512
C	3.209442118880	-3.528539177534	-2.843822453918
C	-0.373750646960	-1.905487168169	2.192753323655
H	-1.261096805594	-2.111177878584	-2.177660102542
C	2.439120058895	-3.659281977287	-0.222894636973
C	-0.593364402907	-2.110219188202	3.544662827471
C	-1.603718828759	-2.409566347773	-1.193703151672
H	2.125245730484	-3.685775146176	0.813848912096
C	-1.261799631294	-2.376893263262	1.106852698882
C	3.547024452089	-4.657466348924	-2.110004157189
C	3.150185585795	-4.716340714791	-0.781666462419
H	-1.465793821270	-2.651855351791	3.888793027116
H	4.102479159796	-5.468655744293	-2.565441976927
C	-2.765471687143	-3.156911793619	-1.035738877060
C	-2.419280206802	-3.121820208726	1.320368684763
H	-2.729685096526	-3.396175448356	2.320394342290
C	-3.186528950993	-3.522629509441	0.235003072264
H	-4.089023523891	-4.103890334579	0.379871182955
S	-0.006423849723	-1.891236377555	6.178770675788
C	1.430235267339	-1.171174168932	7.049995999640
H	1.468613776116	-0.089518831877	6.920574665164
H	2.355694252985	-1.645104175267	6.723883770049
S	2.425792061881	0.625270008884	-6.448922619520
C	1.749993811279	2.297924126953	-6.757052531013
H	0.660008410074	2.288566513982	-6.762428286825
H	2.139488186559	3.011178560842	-6.031321658867
H	1.256943293908	-1.404093475962	8.101370789143

H	2.114088658357	2.560056159030	-7.750786366888
Cl	3.533206815657	-6.110482034570	0.206110589638
Cl	-3.680396130723	-3.622821457621	-2.452969231212
Cl	-2.697240132007	3.415868860084	1.244610665286
Cl	5.969583335733	2.085398871338	-0.276301937716

References

(1) Elsbernd, H.; Beattie, J. K. The NMR spectra of terpyridine and the bis-terpyridine complexes of cobalt(III) and iron(II). *J. Inorg. Nucl. Chem.* **1972**, *34*, 771 – 774.

(2) Sárosiné Szemes, D.; Keszthelyi, T.; Papp, M.; Varga L.; Vankó, G. Quantum-chemistry-aided Ligand Engineering for Potential Molecular Switches: Changing Barriers to Tune Excited State Lifetimes. *Chem. Commun.* **2020**, *56*, 11831–11834.
